# eIF4A2 drives repression of translation at initiation by Ccr4-Not through purine-rich motifs in the 5′UTR

**DOI:** 10.1186/s13059-019-1857-2

**Published:** 2019-12-02

**Authors:** Ania Wilczynska, Sarah L. Gillen, Tobias Schmidt, Hedda A. Meijer, Rebekah Jukes-Jones, Claudia Langlais, Kari Kopra, Wei-Ting Lu, Jack D. Godfrey, Benjamin R. Hawley, Kelly Hodge, Sara Zanivan, Kelvin Cain, John Le Quesne, Martin Bushell

**Affiliations:** 1 Cancer Research UK Beatson Institute, Garscube Estate, Switchback Road, Glasgow, G61 1BD UK; 2 Institute of Cancer Sciences, University of Glasgow, Glasgow, UK; 3 MRC Toxicology Unit, Lancaster Road, Leicester, LE1 9HN UK; 4 Present Address: Division of Cell and Developmental Biology, School of Life Sciences, University of Dundee, Dundee, DD1 5EH UK; 5 Present Address: Department of Chemistry, University of Turku, Vatselankatu 2, FI-20500 Turku, Finland

## Abstract

**Background:**

Regulation of the mRNA life cycle is central to gene expression control and determination of cell fate. miRNAs represent a critical mRNA regulatory mechanism, but despite decades of research, their mode of action is still not fully understood.

**Results:**

Here, we show that eIF4A2 is a major effector of the repressive miRNA pathway functioning via the Ccr4-Not complex. We demonstrate that while DDX6 interacts with Ccr4-Not, its effects in the mechanism are not as pronounced. Through its interaction with the Ccr4-Not complex, eIF4A2 represses mRNAs at translation initiation. We show evidence that native eIF4A2 has similar RNA selectivity to chemically inhibited eIF4A1. eIF4A2 exerts its repressive effect by binding purine-rich motifs which are enriched in the 5′UTR of target mRNAs directly upstream of the AUG start codon.

**Conclusions:**

Our data support a model whereby purine motifs towards the 3′ end of the 5′UTR are associated with increased ribosome occupancy and possible uORF activation upon eIF4A2 binding.

## Introduction

Two mRNA-binding complexes—eIF4F and Ccr4-Not—play fundamental roles in directing the cytosolic fate of mRNAs at the level of translation as well as mRNA turnover. The eIF4F complex, consisting of the cap binding protein eIF4E, the regulatory scaffold protein eIF4G, and the DEAD-box RNA helicase eIF4A, is recruited to the 5′-cap structure of mRNAs and is required for translation [1]. eIF4A stimulates translation initiation and is thought to be required for unwinding of secondary structure in the 5′UTR to facilitate 40S ribosome scanning [2–6] as well as allowing the loading of the mRNA into the 43S pre-initiation complex (PIC) independently of structure unwinding [7].

The miRNA repression apparatus orchestrates the delivery of the Ccr4-Not complex to target mRNAs, resulting in both translational repression and mRNA decay [8–10]. Translational repression, which is the required first step of miRNA-mediated silencing [11], can be induced by the Ccr4-Not complex independently of its deadenylation and degradation activities [12–15]. The Ccr4-Not complex defines mRNA fate and sculpts the translational landscape of the cell [16] well beyond miRNA-mediated repression by binding to mRNAs via its many partner RNA-binding proteins which recognize a number of regulatory sequence motifs. At the core of the Ccr4-Not complex lies the scaffold protein CNOT1, which dictates the complex’s activity through its interactions with other proteins, such as the deadenylases CNOT7 and CNOT8—proteins required at the onset of mRNA decay [16]. The central region of CNOT1 has been shown to be sufficient for this repressive activity, and structural work has revealed that this region of the protein contains a MIF4G domain [17] similar to that responsible for eIF4G’s interactions with eIF4A [18]. This binding surface in CNOT1 has been shown to interact with the DEAD-box protein DDX6 in the context of miRNA-mediated repression [17]. DDX6 is a well-established translational regulator and central component of cytoplasmic mRNA degradation bodies (P bodies) [19–21], but the mode of its recruitment to mRNAs and mechanism of repression remain unknown.

The second DEAD-box protein implicated in miRNA-mediated translational repression is eIF4A2. The two cytoplasmic paralogs of eIF4A, eIF4A1 and eIF4A2, have previously been reported to have redundant functions in translation initiation through their interaction with eIF4G as part of the eIF4F complex [22]. More recent results suggest that they possess distinct activities [4], and our previous work showed that unlike eIF4A1, eIF4A2 is involved in miRNA-mediated repression and associates with the Ccr4-Not complex component CNOT7 [11, 23]. However, there have been reports contesting our findings regarding the activity of eIF4A2 in miRNA-mediated repression [17, 24, 25]. Indeed, the nature of the divergent functions of the eIF4A paralogs and their respective roles in gene regulation are not yet understood.

The molecular mechanism by which miRNAs inhibit translation has been a matter of debate for many years. Original observations showed that miRNAs influence gene expression at a post-initiation stage of translation [26] and were subsequently confirmed by other groups [27–29]. Later, numerous studies showed that repression was exerted at the initiation phase of protein synthesis [30–33]. Investigations focusing on translation repression at initiation have highlighted the critical role of the eIF4F complex in this process [11, 31, 34–36], a claim that has nevertheless been contested [25]. The volume of contradictory data attests to the fact that despite much research, the precise mechanism of miRNA-mediated translational repression remains unresolved.

The present study addresses the fundamental molecular mechanisms of miRNA-mediated repression. We demonstrate that eIF4A2 forms part of a large repressive complex together with CNOT1. We show that eIF4A2 is predominantly associated with mRNAs repressed at initiation in a manner dependent on CNOT1. Interestingly, messages only bound by DDX6 are not enriched for miRNA target families nor are they repressed at initiation; however, mRNAs bound by eIF4A2 are targeted by a distinct set of miRNA families and are translationally upregulated following CNOT1 knockdown. In terms of RNA binding, eIF4A2 has high specificity for purine-rich RNA, similar to that of chemically inhibited eIF4A1 [37]. We show repression via eIF4A2 is associated with the enrichment of purine-rich motifs towards the end of the 5′UTR. We also show eIF4A2-bound messages have an increased prevalence of translation initiation from upstream translation initiation sites.

## Results

### eIF4A2 forms an endogenous complex with CNOT1

eIF4A1 and eIF4A2 have been reported to have non-redundant divergent roles in translation regulation [11, 38, 39], despite sharing 90% amino acid sequence identity (Additional file 1: Figure S1A) and original claims that the two paralogs have identical activities [22]. To gain a better understanding of the nature of the differences between the two proteins, we turned to previously described dominant negative (D/N) mutations of eIF4A1, which disrupt its RNA binding/unwinding capacity but not its interaction with eIF4G [40], leading to the formation of a functionally inactive eIF4F complex. As eIF4A1 and eIF4A2 share sequence identity within this motif (PTRELA, Additional file 1: Figure S1A), we introduced these mutations into both proteins. Expression of D/N eIF4A1 resulted in inhibition of translation of a luciferase reporter, but interestingly, expression of mutant eIF4A2 did not (Additional file 1: Figure S1B). This shows clearly that the two proteins have distinct functions and suggests that eIF4A2 does not interact strongly with eIF4G, as it would otherwise inhibit the eIF4F complex and lead to translational repression. Therefore, we examined the ability of both proteins to interact with eIF4G. As reported previously [11], while eIF4A1 could strongly interact with endogenous eIF4G, eIF4A2 showed only a weak association (Additional file 1: Figure S1C). This was not dependent on the cell line, position of tag nor the type of tag present (Additional file 1: Figure S1C) [11]. Previous reports had shown that eIF4A2 is not able to rescue translation after eIF4A1 depletion [38], and our observations confirm this. Of note, we observed that unphysiologically high levels of eIF4A2 overexpression, as are often observed in transfection experiments, can result in its association with eIF4G (Additional file 1: Figure S1D). This suggests it is possible to oversaturate the system, which might explain conflicting results obtained by others [17, 25]. For this reason, we attempted to perform as many experiments as possible by investigating endogenous complexes.

**Fig. 1:**
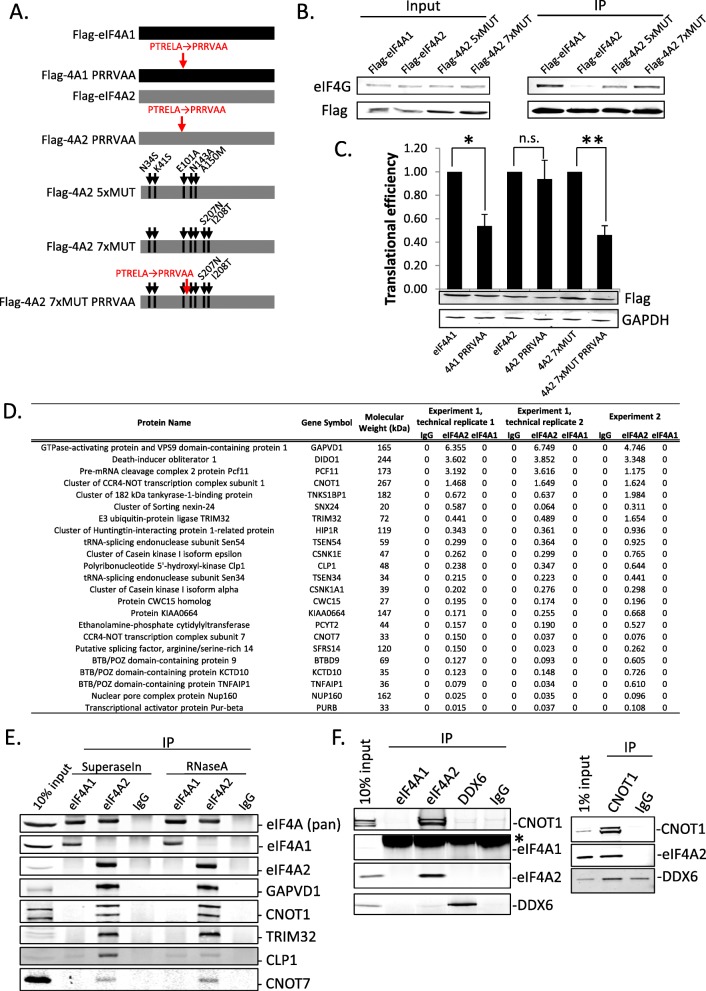
eIF4A2 is not a canonical component of eIF4F, but instead interacts with the Ccr4-Not complex. **a** Schematic of Flag-tagged proteins expressed. **b** Immunoprecipitation of Flag-tagged proteins expressed in HEK293 cells transfected with the indicated constructs. IPs were performed 48 h after transfection, and Western blots were probed with eIF4G antibody to show interaction. Western blot shows a representative experiment of 5. **c** HEK293 cells were transfected with constructs depicted in **a** and a Renilla luciferase reporter plasmid. Cells were harvested after 24 h, luciferase activity was measured and reporter mRNA was quantified by qPCR. Translational efficiency denotes luciferase activity over RNA abundance, graph represents 3 independent experiments, and significance calculated from unnormalized data using Student’s *t* test, *p < 0.05, **p < 0.01. Western blot represents Flag-protein expression levels in one of the replicates . **d** LC-MS/MS analysis of endogenous eIF4A1 and eIF4A2 IPs from HeLa cytoplasmic extract. Table shows quantitation of proteins using emPAI [41] specifically enriched in the eIF4A2 IP. Table shows results from two experiments, one with two technical replicates. **e** Western blot confirmation of selected LC-MS/MS hits with and without RNaseA digestion. IPs were performed for endogenous proteins. The pan-eIF4A antibody recognizes both eIF4A1 and eIF4A2. **f**. RNaseA-treated IPs using indicated antibodies from gel filtration fractions of HeLa lysate enriched in CNOT1 and eIF4A2. The interaction between CNOT1 and DDX6 is not as clear because of high background in the IgG IP (right panel). Asterisk denotes non-specific band from IgG

We next sought to determine the amino acids within eIF4A2 responsible for its altered association with eIF4G. Despite the major sequence divergence between eIF4A1 and eIF4A2 residing in the N-terminus (Additional file 1: Figure S1A), the removal or exchange of this region did not affect the association between eIF4A2 and eIF4G (Additional file 1: Figure S2A). Evolutionary conservation of the amino acids in the N-terminus of the two proteins is rather low; however, a number of other sites which differ between eIF4A1 and eIF4A2 have been conserved following gene divergence (Additional file 1: Figure S2B). Substituting amino acids at 7 specific sites (of a total of 41 non-identical amino acids between the 2 proteins), dispersed along the N-terminal lobe of eIF4A2, into those present in eIF4A1 was sufficient to restore binding to eIF4G (Fig. 1a, b). When the eIF4A1 D/N mutations were added to this variant of eIF4A2, it acquired the ability to repress translation in a dominant negative manner (Fig. 1c). Superimposing these amino acids on the previously solved eIF4A structure [42], we observe that they are all present on the surface of the N-terminal lobe (Additional file 1: Figure S2C), which raised the possibility that they create an interaction site for another binding partner.

We had previously shown that eIF4A2 associates specifically with the deadenylase CNOT7, which is part of the Ccr4-Not complex [11, 23]. To extend our knowledge of the differential binding partners of eIF4A1 and eIF4A2, we conducted LC-MS/MS analysis of immunoprecipitates of both endogenous proteins. These revealed that eIF4A2 interacts with CNOT1, the central component of the Ccr4-Not complex, as well as other components of the Ccr4-Not complex, including CNOT7 (Fig. 1d). This strongly reinforces our previous observations that eIF4A2 associates with Ccr4-Not complex. We confirmed these interactions and showed they were RNA independent (Fig. 1e). Many of these have also been validated in a separate study [23]. Several other proteins identified as highly enriched in the MS/MS analysis in the eIF4A2 IPs are not only known to be part of the mRNA turnover pathway, but are involved in miRNA-mediated repression. For example, TRIM32 has been shown to enhance the activity of miRNAs [43] and associate with many protein components of the repression machinery, including DDX6, in mouse neural progenitors [44]. Another of the interacting proteins, CSNK1A1, was shown to regulate the efficiency of miRNA-mediated repression through the phosphorylation of Ago2 [45]. Similarly, CLP1 acts as an activator of miRNAs [46]. Finally, TNKS1BP1 (also known as TAB182) has been previously identified as a component of the Ccr4-Not complex [47]. Together, our findings show that eIF4A2 interacts with a complex involved in miRNA-mediated repression and the control of translation in general.

We further investigated this endogenous complex by performing gel filtrations of cytoplasmic lysate from HeLa cells, which revealed that both CNOT1 and eIF4A2 are present within the same fractions (and eIF4A1 levels are minimal in these fractions—see inputs Fig. 1f and Additional file 1: Figure S3A) migrating at an approximate molecular weight of 1.3 MDa (Additional file 1: Figure S3B). Reciprocal immunoprecipitations of both eIF4A2 and CNOT1 from these fractions show a strong RNA-independent interaction between them (Fig. 1f). Other groups have been unable to show an interaction between these two proteins [17, 24]. However, the critical difference is that the previous studies used overexpression and partial fragments of CNOT1, which may disturb either the complex formation or the delicate stoichiometry between the regulatory proteins. We, on the other hand, are investigating the endogenous complexes using multiple different technical approaches, and additional findings regarding these interactions have been reported in a recent study [23]. An interaction between CNOT1 and DDX6 is not obviously apparent in IPs from gel filtration fractions because of high background (Fig. 1f, right panel), but is readily detectable in IPs from total lysate (Additional file 1: Figure S3C). This could mean that DDX6- and eIF4A2-containing Ccr4-Not complexes have different molecular weights resulting in differential migration through the gel filtration columns. Together, these data show the existence of an RNA-independent interaction of endogenous eIF4A2 with CNOT1.

### eIF4A2-bound mRNAs are translationally repressed

**Fig. 2:**
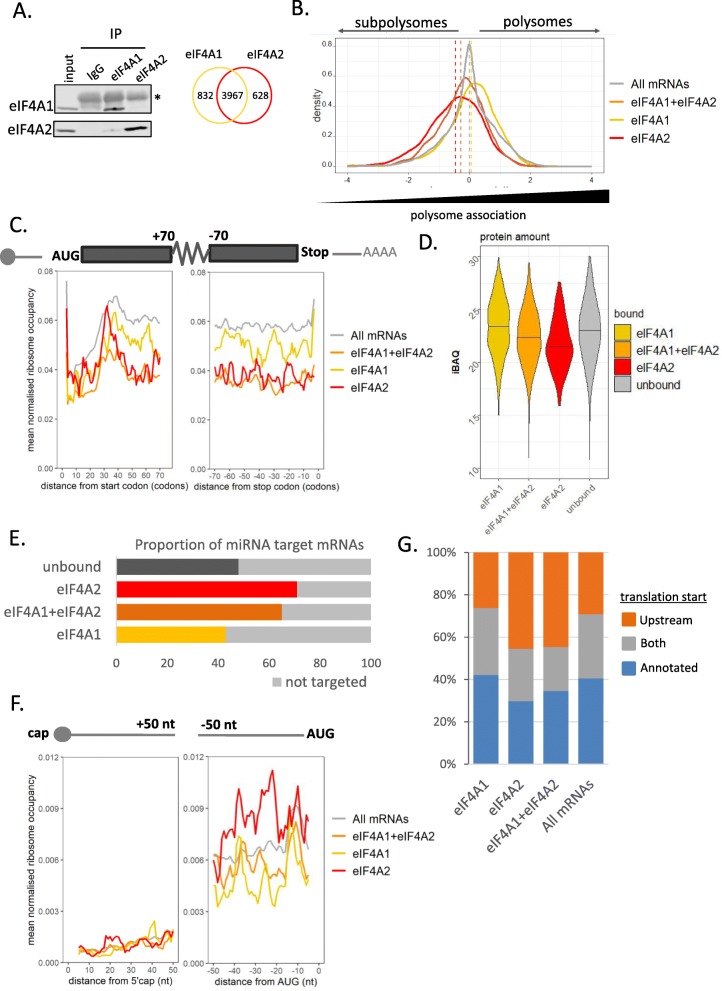
eIF4A2 represses translation at initiation. **a** Western blot demonstrates specificity of immunoprecipitation for each protein from a representative experiment. Input represents 10% of lysate used in IP. Asterisk denotes non-specific signal from IgG. Venn diagram showing numbers of mRNAs significantly (FDR < 0.05) enriched over input in the respective endogenous RIP-Seq (*n* = 3). **b** Differential association with polysomes of mRNAs bound to one of the two proteins or both compared to all mRNAs identified in the RIP-Seq experiment. Relative distribution of mRNAs on sucrose density gradients was calculated from RNA-Seq analysis of the subpolysomal and polysomal fractions in a separate experiment (*n* = 4) by subtracting counts per million between the two fractions. Significance calculated using Dunn’s test with Bonferroni’s correction. **c** Differential ribosome occupancy of eIF4A2- and eIF4A1-bound messages. Ribosome profiling was performed in HEK293 lysates (*n* = 3). Ribosome occupancy for each mRNA at each nt position is calculated as the number of ribosome footprints normalized to the mRNA abundance (transcripts per million—TPM). Shown is the mean number of normalized ribosome footprints 75 codons downstream of the AUG and upstream of the STOP codon. **d** iBAQ—intensity-based absolute quantification [48]—of protein abundance in control conditions in pulsed SILAC for bound mRNAs. **e** Proportions of mRNAs bound by eIF4A1 and eIF4A2 predicted to be miRNA targets by the Targetscan algorithm. **f** eIF4A2-bound mRNAs have increased ribosome occupancy in the last 50 nt, but not in the first 50 nt of the 5′UTR. The RPF coverage was normalized for the abundance of the mRNAs (TPM). **g** Translation of the main AUG start codon is repressed by activation of uORFs in eIF4A2-bound mRNAs. Global translation initiation sequencing (GTI-seq) data from Lee et al. [49], also conducted in HEK293 cells, was used to assess the translation from uORFs in the groups of mRNAs bound by either eIF4A1, eIF4A2, or both. The stacked bars represent the proportions of the groups of mRNAs with active translation from the annotated translation start site, upstream start sites, or both

Having obtained evidence of divergent activities and binding partners of eIF4A1 and eIF4A2, we sought to identify the mRNA interaction landscape of the two eIF4A paralogs at physiological levels by performing endogenous native RIP-Seq (Fig. 2a) [50]. While there was a large overlap between bound mRNAs, our analysis showed discrete groups of mRNAs were enriched in binding to only one paralog (Fig. 2a) and we focused on these in the first instance to identify distinctions between the roles of the two proteins. Specific enrichment was confirmed by RT-qPCR in independent experiments (Additional file 1: Figure S4A).

To gain a better understanding of how the eIF4As affect translation, we conducted sucrose density gradients and performed RNA-Seq on the polysomal and subpolysomal fractions to distinguish mRNAs that are highly associated with ribosomes from those that are not. We then calculated the relative distribution of mRNAs between the subpolysomal and polysomal fractions. This method of analysis allowed us to differentiate between mRNAs that are repressed at initiation of protein synthesis, which we expect to be more subpolysomal, from mRNAs undergoing translation or repressed at elongation, which would be expected to be more polysomal. We used this to evaluate the distribution of mRNAs bound exclusively by each of the eIF4A paralogs, as well as the group bound by both proteins, as identified in the RIP-Seq. As expected for a protein involved in translation initiation, eIF4A1-only associated messages displayed a polysome distribution similar to that of all mRNAs (Fig. 2b, Additional file 1: Figure S4B). In contrast to this, eIF4A2-only bound mRNAs displayed a markedly subpolysomal distribution, suggesting attenuation of translation initiation. This strong association with subpolysomes is particularly striking given the eIF4A2-bound mRNAs possess longer coding regions (Additional file 1: Figure S4D), which one would normally expect to be loaded with more ribosomes than the average mRNA due to their length and as a result be present in the higher polysomal fractions. This distribution is specific to the eIF4A2-bound mRNAs as a group of length-matched mRNAs is not subpolysomal (Additional file 1: Figure S4B, bottom panel). The large group of mRNAs bound by both eIF4A1 and eIF4A2 is also less associated with polysomes than the average mRNA, but not as much as the eIF4A2-only group (Fig. 2b).

To further investigate if the eIF4A2-bound mRNAs are being repressed, we performed ribosome profiling to obtain the precise distribution of the ribosomes along the mRNA. Metagene analysis along the CDS supports the polysome profiling data, showing eIF4A2-bound mRNAs and those bound by both proteins to have reduced ribosome occupancy along the mRNA compared to eIF4A1-bound mRNAs (Fig. 2c). These results could be explained by either an initiation block or a higher elongation speed, as has been suggested before [51, 52]. To differentiate between the two possibilities and test the hypothesis that these mRNAs are repressed at initiation, we turned to proteomic methodologies. Using pulsed SILAC, we showed the polysome distribution of the bound mRNAs is reflected in lower protein levels for mRNAs exclusively bound by eIF4A2 and bound by eIF4A1 and eIF4A2 compared to mRNAs not bound by either of the paralogs (Fig. 2d, Additional file 1: Figure S4C). The decreased protein levels for eIF4A2-bound mRNAs are maintained when comparing to a group of length-matched control mRNAs (Additional file 1: Figure S4C).

Since eIF4A2 has previously been implicated in miRNA-mediated repression [11], and we observe that eIF4A2-bound mRNAs are translationally repressed, we examined the proportions of miRNA targets bound by the protein. mRNAs bound by eIF4A2 or both eIF4A1 and eIF4A2 have a much higher proportion of miRNA targets compared to mRNAs only bound by eIF4A1 (Fig. 2e). This supports the role of eIF4A2, but not eIF4A1, in the miRNA pathway.

### eIF4A2-bound mRNAs display increased ribosome occupancy in the 5′UTR

In a ribosome profiling dataset, the majority of the ribosome-protected fragments (RPFs) align to the CDS. However, RPFs may be observed in the 5′UTR as a result of a block of translation initiation from the main AUG and possible translation of upstream open reading frames (uORFs) [25]. Analysis of our ribosome profiling experiment revealed that eIF4A2-bound mRNAs are enriched for RPF reads in the last 50 nt of their 5′UTR compared to all mRNAs and eIF4A1-bound mRNAs (Fig. 2f). Meanwhile, there is no difference in ribosome occupancy in the first 50 nt of the 5′UTR. One of the explanations for this observation could be the activation of upstream open reading frames (uORFs) leading to reduced translation from the main AUG start codon of the mRNAs.

Recently published global translation initiation sequencing (GTI-seq) [49], also performed in HEK293 cells, used lactimidomycin to obtain peaks of only initiating ribosomes—this allows identification of active uORFs in cells. We utilized these data to ask if the eIF4A2-bound mRNAs have a tendency to possess active uORFs. Of the detected mRNAs in the GTI-Seq dataset, a subset are only translated from their annotated AUG start codon; for some mRNAs, only the upstream translation initiation site is active (referred to as uTIS); and for other mRNAs, initiating ribosome peaks are found at both the upstream and the annotated start sites. Interrogation of the dataset revealed that there is a greater proportion of eIF4A2-bound mRNAs with initiating ribosome peaks only at the upstream and not at the annotated start site compared to all other mRNAs (Fig. 2g). We also see increased numbers of upstream initiation start sites in the mRNAs bound by both eIF4A1 and eIF4A2, but no increased ribosome density in the 5′UTR indicating that these mRNAs may be subject to more complex interplay between the two binding proteins. Looking in more detail at the upstream initiation, we observe no specific trends for start site position (Additional file 1: Figure S5A) or particular start codons driving this upstream initiation in the eIF4A2-bound mRNAs compared to the general features associated with uORFs (Additional file 1: Figure S5 BC). To confirm the observations are due to presence of uORFs and not an extension of the main ORF, we looked at the frame of the uORF and observe the majority of the uORFs are not in frame (Additional file 1: Figure S5DE). This suggests eIF4A2 has a role in reducing translation initiation at the main AUG start codon at least partly on account of the presence of active uORFs in the 5′UTRs of target mRNAs.

### eIF4A2 binds mRNAs belonging to distinct functional groups

Recent studies have highlighted the importance of eIF4A activity in cancer [53–55], and while eIF4A1 expression correlates with cell proliferation, that of eIF4A2 does not [56]. GO term enrichment analysis revealed striking functional differences between the mRNAs bound by the two eIF4A paralogs (Additional file 1: Figure S6). eIF4A2 was associated with mRNAs encoding the miRNA biogenesis apparatus and proteins regulating neural tube development as well as protein involved in negative regulation of transcription—all predominantly nuclear proteins. mRNAs bound by both proteins encode for factors involved in signaling, cell cycle arrest, and translation. Interestingly, eIF4A1-bound mRNAs did not show enrichment for a particular term in this analysis, suggesting the protein is not selectively targeting specific mRNAs.

### eIF4A2 affinity and selectivity for purine-rich RNA is comparable to that of inhibited eIF4A1

**Fig. 3:**
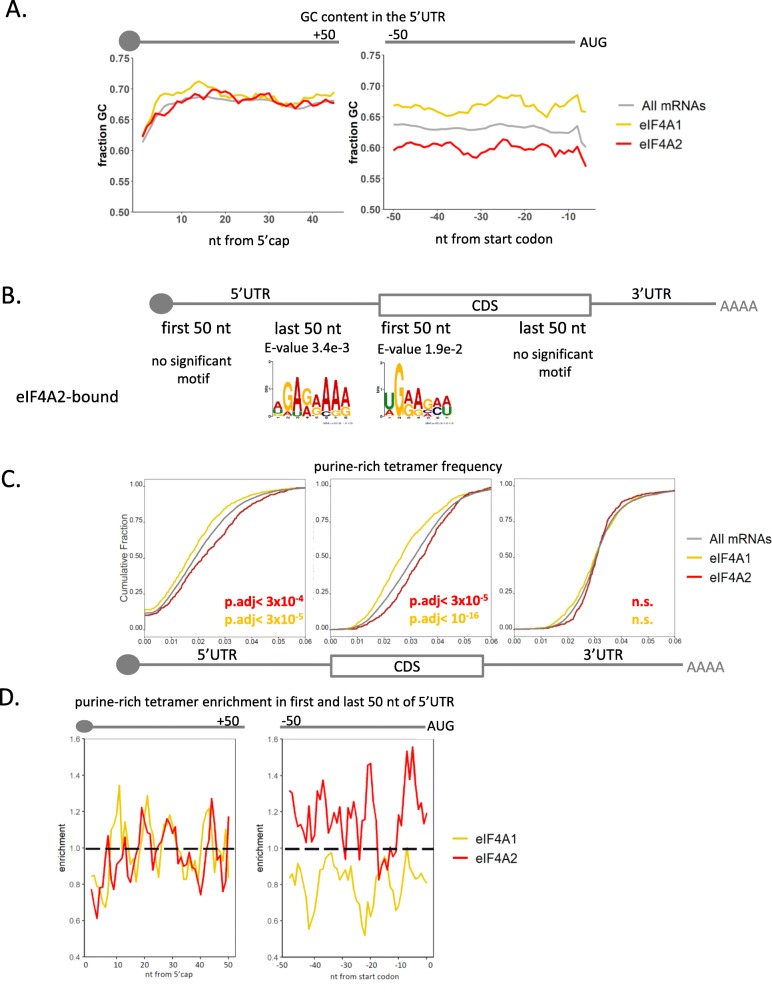
eIF4A2 selectively binds to purine-rich motifs within 5′UTRs. All figures use the groups of mRNAs bound by either eIF4A1 or eIF4A2 as depicted in the Venn diagram in Fig. 2a. **a** 6 nt rolling average GC content in the 5′UTR shows eIF4A1-bound mRNAs have increased GC content at 3′ end of the 5′UTR. **b** Motif enrichment analysis of eIF4A2-bound mRNAs compared to eIF4A1-bound mRNAs as controls was carried out for the first and last 50 nt of the 5′UTRs and coding sequences (CDS) using the MEME algorithm from the MEME Suite [57]. Shown are enriched motifs with associated probabilities. **c** eIF4A2-bound mRNAs have a higher frequency of purine-rich motifs (AAGA, AGAA, GAAA, GAGA, AGAG, GGAA, AAAA, GAAG) identified as targets of eIF4A clamping following chemical inhibition by RocA [37] in the 5′UTR and CDS. Cumulative frequency plots depicting frequencies of purine-rich motifs in 5′UTRs, CDSes, and 3′UTRs, respectively, of bound mRNAs. Significance calculated using Dunn’s test with Bonferroni’s correction. **d** eIF4A2-bound mRNAs have enrichment of purine-rich motifs directly upstream of the AUG start codon. The first and last 50 nt of 5′UTRs of mRNAs bound by either eIF4A1 or eIF4A2 were used in analysis of enrichment over all mRNAs identified in the RIP-Seq experiment. Significance calculated using Dunn’s test with Bonferroni’s correction

We next examined other sequence attributes of eIF4A-bound mRNAs in detail. It had previously been reported that the main function of eIF4A in translation initiation was to unwind secondary structure in the 5′UTR and several studies revealed the presence of specific GC-rich sequence motifs in 5′UTRs of mRNAs regulated by eIF4A1 [53–55]. Our experiments confirm that the eIF4A1-bound mRNAs have higher 5′UTR GC content, especially in the last 50 nt upstream of the AUG start codon (Fig. 3a). To investigate whether the eIF4A2-bound mRNAs bore any specific sequence motifs, we conducted an unbiased motif search on the beginning and end of both the 5′UTRs and coding sequences. This showed eIF4A2-only bound messages have a specific enrichment for purine-rich motifs in the last 50 nt of the 5′UTR and at the beginning of the coding region compared to the eIF4A1-specific mRNAs (Fig. 3b). Interestingly, it was previously reported that the inhibitor RocA transforms eIF4A1 into a translational inhibitor that clamps onto purine-rich motifs within 5′UTRs thus preventing 40S scanning [37]. We confirmed that 5′UTRs and coding sequences (CDS) of mRNAs bound only by eIF4A2 are significantly enriched for purine-rich tetramers that were identified as being the most commonly bound by inhibited eIF4A1 in the RocA study (Fig. 3c), especially directly upstream of the AUG start codon (Fig. 3d). Taken together with the observation that eIF4A2-bound messages have increased ribosome occupancy in the 5′UTR compared to all mRNAs (Fig. 2f), as was seen for 5′UTRs of mRNAs sensitive to inhibition of eIF4A1 by RocA by Iwasaki et al. [37], this suggested to us that uninhibited eIF4A2 might be displaying similar activity to RocA-inhibited eIF4A1. Under normal conditions, eIF4A1 is not expected to act as a clamp and thus should not have a binding preference for mRNAs containing purine-rich motifs, and this is what we observe in the following set of experiments addressing both RNA-binding specificity and selectivity.

**Fig. 4:**
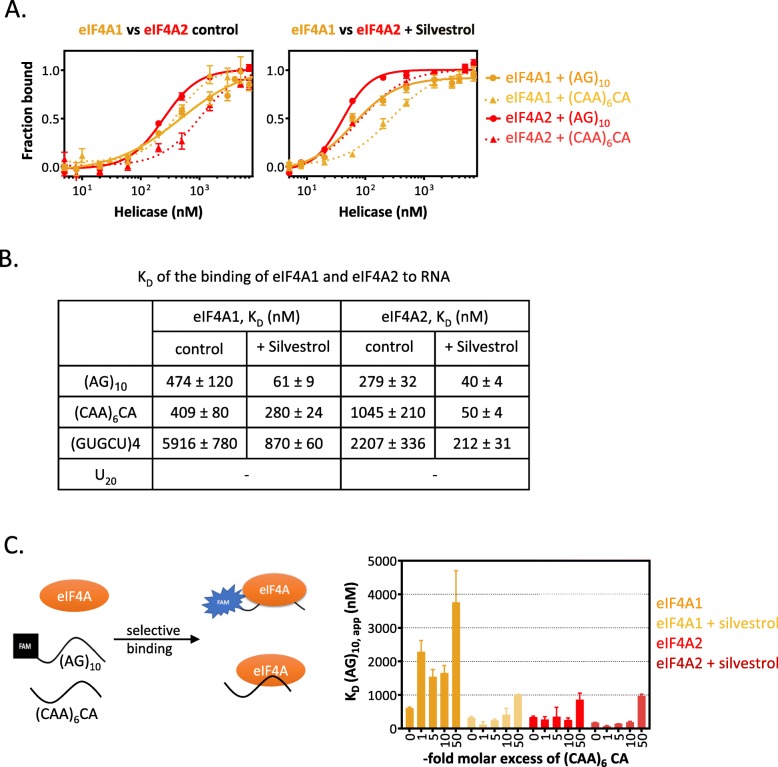
**a** Equilibrium binding of eIF4A1 and eIF4A2 to RNA substrates. Direct fluorescence polarization measurement of 5′ FAM-RNA/eIF4A association for wild-type eIF4A1 (gold) and eIF4A2 (red) in the absence (control) or presence (+ silvestrol) of 10 μM silvestrol. Representative plot for 20 nt (AG)_10_ (solid lines), (CAA)_6_CA (dashed lines) oligo association. Raw data were converted to changes in anisotropy. Shown are the mean ± SD of triplicates. **b** Dissociation constants (KD) of the binding of eIF4A1 and eIF4A2 to different FAM-labeled RNAs in the presence and absence of silvestrol. **c** Schematic representation of the competition experiments with simultaneous incubation of multiple RNAs with eIF4A. The binding of eIF4A1 and eIF4A2 with and without silvestrol to labeled (AG)_10_ in the presence of (CAA)_6_CA competitor ssRNA at increasing molar excess was analyzed using EMSA. Dissociation constants have been derived from fitting the binding data. Data represents mean ± SD, *n* = 3

To test the intrinsic capacity of these proteins for RNA-binding, we performed in vitro assays with recombinant proteins. These showed that only eIF4A2 has higher affinity and specificity for a single stranded unstructured purine-rich RNA (ssRNA) composed of (AG) repeats compared to a CA-only RNA, both with and without the presence of the chemical inhibitor silvestrol (Fig. 4a, b, Additional file 1: Figure S7AB), which acts in the same manner as RocA [58]. Meanwhile, eIF4A1 binds both ssRNAs at a comparably high affinity. In contrast, both proteins show a tenfold weaker affinity to a hairpin GCU-RNA (Fig. 4b). Addition of silvestrol increases binding of both proteins to RNA, regardless of sequence (Fig. 4a, b, Additional file 1: Figure S7A), as has been reported for eIF4A1 previously [37]. Neither protein showed any appreciable affinity for double-stranded RNA (dsRNA) with or without silvestrol (Additional file 1: Figure S7AB). Competition experiments, where one ssRNA is pre-bound to the protein and the competing purine-only RNA is added afterwards, showed that both eIF4A paralogs have clamping properties on a purine-only (AG_10_) oligo, especially when compared to eIF4H, a stimulator of eIF4A activity with known low RNA-binding capacity [59] (Additional file 1: Figure S7C). However, only eIF4A2 readily exchanged a CA-only RNA for a purine-only RNA (Additional file 1: Figure S7C), supporting the idea that the two paralogs show differences in selectivity of RNA binding. Importantly, selectivity experiments both in the presence of unhydrolysable AMPPNP (Fig. 4c) as well as ATP (Additional file 1: Figure S7D), in which the proteins are presented two different RNAs at once, demonstrate a lack of selectivity of eIF4A1 in RNA binding, while eIF4A2 displays a strong preference in binding a purine-only RNA even under high molar excess of competitor RNA (Fig. 4c). This observation is consistent with the difference in affinity and kinetic stability of eIF4A2 with the CA-RNA (Fig. 4b and Additional file 1: Figure S7C). Thus, uninhibited eIF4A2 shows selectivity for purine sequences that is at similar levels to silvestrol-inhibited eIF4A1 (Fig. 4c).

To confirm the in vitro results in the cellular context, we conducted RNA-IPs for eIF4A1 and eIF4A2 with and without RocA treatment (Additional file 1: Figure S8A) followed by qPCR of the previously validated targets (Additional file 1: Figure S4A). Following RocA treatment, there is a strong increase in binding of eIF4A1 to eIF4A2 targets (Additional file 1: Figure S8B), whereas we see minimal impact on their binding to eIF4A2, which is already bound to these mRNAs in control conditions. This is what we would predict given previous data for eIF4A1 acting as a translational repressor following RocA treatment [37].

Together, these data suggest that eIF4A2 has high affinity for and selective interaction with purine motifs within mRNAs similar to that of inhibited eIF4A1, both in vitro and in vivo. The enrichment of these motifs in eIF4A2-bound mRNAs concurrently with an accumulation of ribosome footprints in the 5′UTR is an indication that in this endogenous context, eIF4A2 shows similarities to chemically inhibited eIF4A1 [37].

### eIF4A2 represses translation of miRNA targets at initiation via CNOT1

**Fig. 5:**
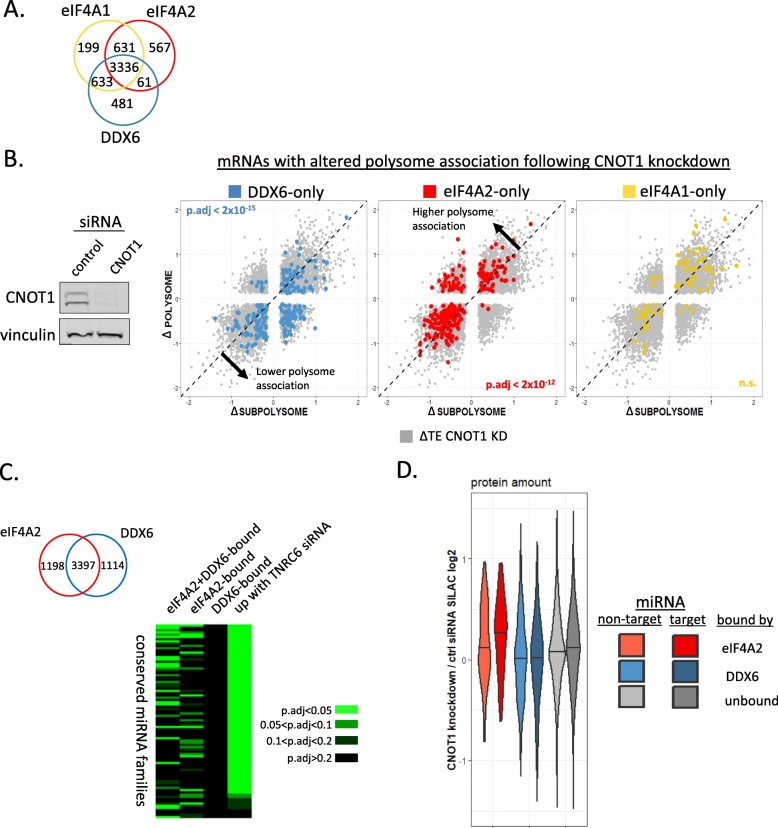
Different miRNA families target mRNAs bound by eIF4A2 alone or eIF4A2 and DDX6. **a** Venn diagram showing numbers of mRNAs enriched in RIP-Seq of eIF4A1, eIF4A2, and DDX6. **b** Depletion of CNOT1 shifts mRNAs bound by eIF4A2 into polysomes and mRNAs bound by DDX6 alone out of polysomes, while eIF4A1-bound mRNAs do not show a consistent shift. RNA-Seq experiment *n* = 4. Significance was calculated using the Dunn test with the Benjamini-Hochberg correction. Western blot shows a representative CNOT1 knockdown experiment confirming efficient knockdown with vinculin as loading control. **c** Venn diagram shows numbers of mRNAs enriched in RIP-Seq when only considering eIF4A2 and DDX6. mRNAs bound by eIF4A2, DDX6, or both (eIF4A2 + DDX6) as well as mRNAs upregulated following TNRC6A/B knockdown in HEK293 cells (FDR < 0.05) were categorized according to target prediction for conserved miRNA families (Targetscan [61]). Enrichment of mRNAs targeted by a particular miRNA family (for full list of families, see Additional file 2: Table S1) in each group was assessed using Fisher’s exact test. Heatmap presents enrichment below an adjusted *p* value (FDR) of 0.05, as well as between 0.05 and 0.1 and between 0.1 and 0.2, to show consistency even with lower stringency cutoffs. **d** Pulsed SILAC labeling for 14 h was performed following 34 h of CNOT1 or control knockdown. Violin plot shows ratios of labeled proteins for proteins encoded by mRNAs bound by indicated proteins. Each group was divided into miRNA “target” and “non-target” as assessed by up- or downregulation following TNRC6A/B knockdown

Since eIF4A2 had been previously implicated in miRNA-mediated repression [11], it interacts with the Ccr4-Not complex, and eIF4A2-regulated mRNAs were involved in the miRNA pathway, we further investigated its role in the miRNA silencing mechanism. We have shown that eIF4A2 interacts with the Ccr4-Not complex, but there is also ample evidence for DDX6 playing a role in imposing miRNA-mediated repression via the Ccr4-Not complex [4, 11, 17, 34, 35], which is the principal effector of translational repression and mRNA degradation induced by miRNAs [60]. We therefore extended our RIP-Seq study by performing DDX6 RNA-IPs to be able to compare the mRNA-binding repertoires of the two Ccr4-Not-interacting DEAD-box proteins (Fig. 5a, Additional file 1: Figure S9A). This revealed a number of mRNAs bound uniquely to eIF4A2 or DDX6, as well as eIF4A1 (Fig. 5a, Additional file 1: Figure S9A).

Thus far, the data presented has suggested that mRNAs bound by eIF4A2 are repressed at initiation, possibly through the interaction with the Ccr4-Not complex. To test this hypothesis, we performed RNA-Seq on subpolysomal and polysomal fractions of sucrose density gradients from cells with and without depletion of CNOT1 (Fig. 5b, Additional file 1: Figure S9B). We then compared the relative changes in polysome association of mRNAs bound by the two Ccr4-Not partner proteins—eIF4A2 and DDX6, and eIF4A1. Knockdown of CNOT1 caused eIF4A2-only bound mRNAs to shift into the polysomal fraction, as expected for mRNAs released from repression at initiation (Fig. 5b). Unexpectedly though, DDX6-only bound mRNAs shifted into the subpolysomal fraction (Fig. 5b). This is not what would have been expected for a protein interacting with mRNAs translationally repressed by the Ccr4-Not complex. We confirmed these observations by RT-qPCR along every fraction of the polysome gradient for representative mRNAs that were also predicted miRNA targets (Additional file 1: Figure S10). Meanwhile, the eIF4A1-only bound mRNAs did not show a trend in shifting to either the sub- or the polysomal fractions (Fig. 5b). We examined the mRNAs that shifted in polysome distribution following CNOT1 depletion for conserved miRNAs targets and subdivided these based on association with eIF4A1, eIF4A2, and DDX6. This analysis revealed that predicted miRNA targets are more associated with eIF4A2 than either eIF4A1 or DDX6 (Additional file 1: Figure S11C).

To further investigate this, we focused on DDX6 and eIF4A2 on account of their described role in both the Ccr4-Not complex and miRNA-mediated regulation [11, 17, 23, 42], as opposed to eIF4A1 which is associated with the eIF4F initiation complex [18, 53, 55] (Fig. 1, Additional file 1: Figure S1). We subdivided our RIP-Seq results between eIF4A2 and DDX6 and examined the mRNAs bound specifically to only one or bound by both proteins for miRNA target sites in their 3′UTRs. To create a reference dataset, we conducted RNA-Seq following knockdown of TNRC6A/B, the two highly expressed members of the TNRC6 family of proteins which are the key effectors of the miRNA pathway [62] (Additional file 1: Figure S9C). miRNA targets are specifically upregulated following TNRC6 depletion [8], and thus, this dataset can be considered to be a faithful representation of miRNA targets in our cell line. Enrichment analysis for miRNA targets among mRNAs bound by eIF4A2 and DDX6 was compared to mRNAs upregulated following TNRC6A/B depletion. This revealed, as expected, that targets of many conserved miRNA families were increased after TNRC6A/B knockdown (Fig. 5c). Strikingly, different sets of miRNA families were enriched among mRNAs only bound by eIF4A2 and those bound by both eIF4A2 and DDX6 (Fig. 5c, Additional file 2: Table S1). mRNAs bound only by DDX6 did not display significant enrichment for any conserved miRNA families (though this of course does not mean there are not many miRNA targets in this group, only that the mRNAs are not enriched for conserved miRNA family targets). Interestingly, from our RIP-Seq, we observed that DDX6 associated strongly with mRNAs encoding P-body components, RNA processing machinery, and proteins involved in mitochondrial function, most of which are cytosolic in nature (Additional file 1: Figure S9E). It is remarkable that the repertoire of mRNAs bound by DDX6 comprises so many mRNAs encoding proteins involved in the very pathways DDX6 has been shown to be active in, suggesting that DDX6 may form an RNA regulon [63].

As DDX6 has been implicated in translational repression, we asked whether we observe evidence of reduced ribosome occupancy for mRNAs bound by the protein. When comparing mRNAs bound specifically by either eIF4A2 or DDX6 (see Venn diagram Fig. 5c), our ribosome profiling data showed that eIF4A2-bound mRNAs had much lower ribosome occupancy than mRNAs specifically depleted in the eIF4A2 IPs (Additional file 1: Figure S11A). In contrast, DDX6-bound mRNAs showed minimal differences in ribosome occupancy compared to mRNAs depleted in the DDX6 IPs and we were able to confirm that the polysome association of these mRNAs is puromycin sensitive (Additional file 1: Figure S11B). DDX6 is reported to stimulate decapping, which is preceded by deadenylation of the mRNA, so we further looked at the poly(A) tail length of DDX6-bound mRNAs using data from Subtelny et al. [64] This shows DDX6-bound mRNAs have shorter poly(A) tails compared to mRNAs not enriched for binding either eIF4A2 or DDX6 and eIF4A2 (Additional file 1: Figure S11D). Dividing the bound mRNAs by whether or not they are targeted by miRNAs then highlighting only the predicted miRNA target mRNAs shows the same polysome shifts following CNOT1 depletion for eIF4A2-bound and DDX6-bound mRNAs shown in Fig. 5b (Additional file 1: Figure S11E).

This led us to ask what the effects of CNOT1 depletion were on protein output from these mRNAs. For this, we performed pulsed SILAC experiments with or without knockdown of CNOT1. eIF4A2-bound mRNAs showed increased levels of proteins following CNOT1 depletion, as opposed to those bound by DDX6 (Fig. 5d). We subdivided these groups, designating those upregulated in the TNRC6A/B knockdown RNA-Seq as genuine targets of miRNAs in our cells. As expected for a protein involved in this pathway, eIF4A2-bound miRNA targets were expressed at levels over and above non-target eIF4A2-bound mRNAs upon CNOT1 depletion (Fig. 5d). In fact, when we consider all mRNAs immunoprecipitated with respect to whether they are enriched either in the eIF4A2 IP versus the DDX6 IP, we see a robust increase in protein expression of miRNA targets following CNOT1 depletion in those preferentially bound by eIF4A2 (Additional file 1: Figure S9F). Meanwhile, no such effects were observed for the DDX6-bound mRNAs. This further supports the role of eIF4A2 in miRNA-mediated repression through its interaction with the Ccr4-Not complex.

## Discussion

The two closely related paralogs of eIF4A, eIF4A1 and eIF4A2, have been previously shown to have similar activity in in vitro assays [22]. However, recent studies examining their function in cellular systems have found their actives differ and that they are in fact not functionally redundant [11, 23, 38, 65, 66]. Unlike eIF4A1 which is part of the eIF4F complex, a number of reports have indicated that eIF4A2 interacts with components of the Ccr4-Not complex and is involved in miRNA-mediated repression [11, 23, 65, 67], although this has also been contested [17, 25, 68]. The details of how these two closely related proteins differ were unknown. Here, we show that the two paralogs of eIF4A differ in their abilities to interact with eIF4G and determine the amino acids that distinguish these characteristics (Fig. 1a–c, Additional file 1: Figure S1 and S2). We further validate the interaction of endogenous eIF4A2 with components of the Ccr4-Not complex (Fig. 1d–f, Additional file 1: Figure S3). Previous reports had identified endogenous eIF4A2 interacting with the Ccr4-Not complex [11, 23, 65, 67], while others using overexpression approaches with either full-length or truncated versions of these proteins have refuted these observations [17, 25]. These discrepancies are most likely due to different technical approaches, and importantly, our work has focused on characterizing endogenous complexes.

eIF4A, the archetypal DEAD-box protein, has long been thought to act primarily as a helicase which unwinds secondary structure in the 5′UTRs. DEAD-box proteins are known to also possess strand annealing and clamping capacity [4, 6, 69]. The third paralog of eIF4A, eIF4A3, functions as a molecular clamp as part of the exon junction complex [70] and preferentially binds to a purine-rich sequence motif [71]. In addition, eIF4A1 has been shown to become a clamp upon chemical inhibition with a silvestrol derivative, binding to purine-rich motifs and preventing ribosome progression along the mRNA [37]. We have provided evidence that eIF4A2-bound mRNAs are repressed at translation initiation, and we find that these mRNAs are enriched in their 5′UTR for purine motifs, ribosome occupancy, and uORFs (Figs. 2 and 3, Additional file 1: Figure S5), similar to eIF4A1 inhibited with RocA on purine-rich motifs near the start codon [37]. This repression at initiation results in these mRNAs producing less protein (Fig. 2d). Interestingly, purine motifs downstream of the uTIS have been shown to be sufficient to increase translation from the uTIS [25]. Our data is in line with this, and we see examples of purine motifs both within and outside of the uORF. However, we lack sufficient resolution to make a definitive conclusion about the precise location of these motifs and uTIS utilization.

Strikingly, in vitro experiments using purified proteins show that eIF4A2 binds purine-rich oligos with high selectively and affinity (Fig. 4, Additional file 1: Figure S7). We observe that the selectivity of eIF4A2 for AG-RNA is similar to that of silvestrol-inhibited eIF4A1 in the presence of either AMPPNP or ATP (Fig. 4c, Additional file 1: Figure S7D). While this similarity is striking, the underlying molecular mechanism may be different; in fact, our data show that the off-rate for AG-RNA is different between eIF4A2 and silvestrol-inhibited eIF4A1 (Additional file 1: Figure S7C).

From our RIP-seq data, we see that mRNAs associated with eIF4A2 are enriched for purine sequence motifs (Fig. 3b–d). In cells, we used RIP-qPCR to further demonstrate that RocA enables eIF4A1 to recover these eIF4A2 purine-rich targets (Additional file 1: Figure S8). Common sequence motifs can provide a mechanism for co-regulation, sorting, and subcellular co-localization of RNA regulons [63]. mRNAs that were enriched in the eIF4A2 IPs are also highly enriched for mRNAs stored in P bodies [72] (Additional file 1: Figure S9D), suggesting that mRNAs interacting with eIF4A2 are targeted to sites of mRNA storage. Meanwhile, DDX6-bound mRNAs are enriched for mRNAs encoding component proteins of P bodies and RNA turnover machinery (Additional file 1: Figure S9E) and thus may be involved in regulating their expression—a possibility that requires further investigation.

The Ccr4-Not complex has been shown to have multiple roles in the regulation of gene expression [16] and is recruited to mRNAs targeted by miRNAs where it is believed to act to deadenylate and translationally repress miRNA-targeted mRNAs [8–15]. Repression imposed by miRNAs and the Ccr4-Not complex has been shown by many groups to operate at the level of translation [30–33]. Here, we show that the mRNAs that interact specifically with eIF4A2 are enriched for predicted miRNA target sites (Figs. 2e and 5c, Additional file 1: Figure S11C) and that depletion of the Ccr4-Not complex component CNOT1 leads to their redistribution onto polysomes (Fig. 5b, c, d, Additional file 1: Figure S11E), consistent with translation repression at initiation. eIF4A2-associated mRNAs also show increased protein production by pulsed SILAC (Fig. 5d), together suggesting the Ccr4-Not complex is required for repression of these mRNAs. There are of course many mRNAs that our RIP-Seq showed as binding to both eIF4A1 and eIF4A2, and these mRNAs may be regulated by multiple mechanisms and the two proteins may act in tandem. Interestingly, a recent publication showed that eIF4A2 and CNOT1 cooperate in the unusual mechanism through which the HCV virus utilizes host miR-122 molecules for activation [65].

We have characterized the endogenous complex in which eIF4A2 associates with the Ccr4-Not complex and find it to contain multiple components of translation repression machinery (Fig. 1d–f, Additional file 1: Figure S3). Previous studies had not investigated purely endogenous complexes [17, 24], and this has likely been the reason for conflicting results. The use of RIP-seq to obtain eIF4A2 and DDX6 mRNA targets has provided a transcriptome-wide view of their roles in translational regulation and allowed us to examine mRNAs predicted to be regulated by miRNAs (Fig. 5c, d, Additional file 1: Figure S11C). 

Despite there being ample evidence for the interaction of DDX6 with the Ccr4-Not complex, our data does not suggest that DDX6 is specifically associated with predicted miRNAs’ target mRNAs (Fig. 5c, Additional file 1: Figure S11C). This is perhaps explained by DDX6 having a broader role in mRNA regulation, as has been suggested previously [21]. It cannot be excluded that DDX6 exerts post-initiation repression on bound mRNAs, as has been suggested for the yeast homolog Dhh1 [73], but our proteomic studies were unable to show an upregulation of DDX6-associated mRNAs following CNOT1 depletion (Fig. 5d, Additional file 1: Figure S9F) which may demonstrate that its role within the Ccr4-Not complex is more complicated than previously suggested. What we do observe instead is DDX6-bound mRNAs showing a slight shift out of polysomes following CNOT1 knockdown (Fig. 5b), which might mean these mRNAs are subject to compensatory translational “buffering” [74].

## Conclusions

We have demonstrated that eIF4A2 has distinct activity from eIF4A1 and that it acts to repress initiation of translation of bound mRNAs. Our data implies that this occurs though binding of purine-rich motifs. Moreover, we show that eIF4A2 is in complex with CNOT1 and is involved in miRNA-mediated repression in conjunction with the Ccr4-Not complex.

## Materials and methods

### Cell culture

All cell lines are maintained in Dulbecco’s modified Eagle’s medium (DMEM, GibCo) fortified with 10% fetal bovine serum and 2 mM l-glutamine. Cells were mycoplasma tested.

### Plasmid constructs and mutagenesis

Flag-eIF4A1 and Flag-eIF4A2 constructs were as previously described [11]. The 4A2N-4A1C and 4A1N-4A2C mutants were made by introducing BamHI sites as silent mutations into eIF4A1 and eIF4A2 coding sequence using mutagenesis primers 4A1 E18 BamHI mutF, 4A1 E18 BamHI mutR, 4A2 D18 BamHI mutF, and 4A2 D18 BamHI mutR (see Additional file 2: Table S2). These constructs were then digested with BamHI, and the inserts were cloned into a similarly digested reciprocal Flag construct. The 4A1Δ1-16 and 4A2Δ1-13 constructs were created by introducing SalI and BamHI (Additional file 2: Table S2), respectively, into the coding sequences and digesting out the intervening insert. Point mutations were introduced by site-directed mutagenesis (SDM) using native PfuUltra (Agilent). Mutations introduced into eIF4A2 sequence: Flag-eIF4A2 5xM: N34S, K41S, E101A, N143A, A150M; Flag-eIF4A2 7xM: N34S, K41S, E101A, N143A, A150M, S207N, I208T, and dominant negative mutations were introduced by SDM using primers listed in Additional file 2: Table S2. pRL-SV40 plasmid used in luciferase assay experiments was described previously [11]. N-terminal His-HA-tagged eIF4A1 and eIF4A2 were generated by excising either C-terminal flag-myc tagged eIF4A1 (Origene Inc.) or untagged eIF4A2 described previously [11] with AsiSI and NotI (NEB) restriction enzymes. The excised product was then gel purified and re-ligated into the N-terminal His-HA tagged pCMV6 backbone (Origene Inc., ps10017). A stop codon was inserted after the eIF4A1 ORF to remove extra linker residues. The Flag-eIF4G plasmid was a kind gift from Mark Coldwell. Primers used are listed in Additional file 2: Table S2.

### Tagged protein immunoprecipitations

Immunoprecipitation of Flag-tagged proteins was performed as described previously [11], with the exception that proteins were eluted from beads after washing using 200 ng/μl 3×Flag peptide (Sigma). Immunoprecipitation of HA-tagged proteins was performed using the same conditions, except that anti-HA agarose beads (Sigma A2095) were used and elution was performed with the HA peptide (Sigma I2149).

### Immunoprecipitations for mass spectrometry and gel filtration fractions

Cytoplasmic HeLa lysate (Ipracell) was diluted in buffer (20 mMTris-HCl pH 7.5, 200 mM NaCl, 2.5 mM MgCl2, 0.5% Triton X-100) and precleared by incubation at 4C for 1 h with rotation in the presence of Dynabeads ProteinG (Invitrogen). The precleared lysates were incubated with antibody (eIF4A1—abcam ab31217; eIF4A2—abcam 31218; rabbit IgG—Santa Cruz sc-2027). After an hour, protein G Dynabeads preblocked with BSA and tRNA were added and the mixture incubated for another 2 h. Beads were washed three times for 10 min and then resuspended in SDS-PAGE loading buffer and analyzed by mass spectrometry. Immunoprecipitation following gel filtration was performed as above with the following modifications: The buffer used was 5% (w/v) sucrose, 0.1% (w/v) CHAPS, 20 mM HEPES/NaOH, 5 mM DTT, and 50 mM NaCl, pH 7.0. Antibodies as stated above with the addition of DDX6 (abcam ab70955) and CNOT1 (Protein Technologies 14276-1-AP). Where indicated, RNaseA was added to the IP buffer at a concentration of 5 μg/ml and SuperaseIn at 10 U/ml.

### Luciferase assays

For dominant negative experiments, 6 × 10^4^ HEK cells were plated per well in a 24-well plate. Cells were transfected using GeneJammer and 150 ng protein-encoding plasmid, 10 ng pRL-SV40, and 40 ng pGL3. Cells were harvested after 48 h, and luciferase assays were performed as described previously [11].

### Mass spectrometry

Protein samples were separated on SDS-PAGE gels, Coomassie stained, serially sectioned, and digested with trypsin overnight, and peptides extracted and dried before analysis on a Synapt G2S mass spectrometer as described previously [75–77]. HDMSe data were processed and searched using Proteinlynx Global Server (Waters, Manchester, UK) against a reversed human Swissprot database. The results were visualized using Scaffold (Proteome Software, OR, USA), the filters were set at high stringency to give a protein FDR of 0.0%, and the emPAI results were generated using quantitative analysis in Scaffold. PLGS data files were then loaded in into Scaffold (Proteome Software. Portland, OR, USA), and peptide counts (SAF, spectral abundance factor) calculated as previously described [75].

### SILAC

SILAC-labeled HEK293 cells were obtained by culturing in SILAC-DMEM lacking arginine and lysine (Life Technologies) supplemented with [13C6] l-arginine and [13C6] [15 N2] l-lysine(SILAC medium—M) (Sigma-Aldrich) or [13C6][15 N4] l-arginine and [2H4] l-lysine (SILAC heavy—H; Cambridge Isotope Laboratories, Tewksbury, MA) for 14 h. Each comparison was done in the forward (H/M) and reverse (M/H) directions. After this, cells were harvested into SDS-free RIPA buffer. One hundred fifty micrograms of each quantified SILAC-labeled lysates was mixed in a 1:1 ratio, total protein amount of 300 μg. Samples were then reduced with DTT, to a final concentration of 5 mM, and alkylated with IAA, final concentration of 50 mM. Samples were then subject to a two-step digestion, firstly with Endoproteinase Lys-C (ratio 1:33 enzyme:lysate) for 1 h at room temperature then with trypsin (ratio 1:33 enzyme:lysate) overnight at 37 °C. The digested SILAC samples were fractionated using reverse phase chromatography at pH 10. Solvents A (98% water, 2% ACN) and B (90% ACN, 10% water) were adjusted to pH 10 using ammonium hydroxide. Three hundred micrograms of digested peptides were loaded onto a Kinetex C18 column (150 × 2.1 mm) coupled with a Dionex Ultimate 3000 HPLC system, software version 6.7 (Chromeleon). Injected peptides were subject to a two-step gradient, 4–27% solvent B in 36 mins then 27–48% solvent B in 8 min. The flow rate was set to 200 μl/min. The samples were collected into 21 fractions. Peptide samples were run on the Q-Exactive HF mass spectrometer coupled to an EASY-nLC II 1200 chromatography system (Thermo Scientific). Samples were loaded into a 20-cm fused silica emitter, packed in-house with ReproSIL-Pur C18-AQ, 1.9 μm resin, which was heated to 35 °C using a column oven (Sonation). Peptides were eluted at a flow rate of 300 nl/min over three optimized two-step gradient methods for fractions 1–7, 8–15, and 16–21. Step 1 was commenced for 20 min, and step 2 for 7 mins. For fractions 1–7, the percentage of solvent B was 2–20% at step 1 and 39% at step 2; for fractions 8–15, 4–23% at step 1 and 43% at step 2; and for fractions 16–21, 6–28% at step 1 and 48% at step 2. Peptides were electrosprayed into the mass spectrometer using a nanoelectropsray ion source (Thermo Scientific). An Active Background Ion Reduction Device (ABIRD, ESI Source Solutions) was used to decrease air contaminants. Data was acquired with the Xcalibur software (Thermo Scientific) in positive mode utilizing data-dependent acquisition. The full scan mass range was set to 375–1400 m/z at 60,000 resolution. Injection time was set to 20 ms with a target value of 3E6 ions. HCD fragmentation was triggered on the 15 most intense ions for MS/MS analysis. MS/MS injection time was set to 50 ms with a target of 5E2 ions. Ions that have already been selected for MS/MS were dynamically excluded for 25 s. MS raw data was processed using MaxQuant software [78] version 1.6.3.3 and searched with the Andromeda search engine [79] against the Uniprot *Homo sapiens* database (95,146 entries). First and main searches were done with a precursor mass tolerance of 20 ppm and 4.5 ppm, respectively. MS/MS mass tolerance was set to 20 ppm. Minimum peptide length was set to 6 amino acids, and trypsin cleavage was selected allowing up to 2 missed cleavages. Methionine oxidation and N-terminal acetylation were selected as variable modifications and carbamidomethylation as fixed modification. False discovery rate for peptide and protein identification was set to 1%. SILAC multiplicity was set to 3, and the medium (Arginine 6 and Lysine 4) and heavy (Arginine 10 and Lysine 8) labels were selected. MaxQuant output was processed using Perseus software [80] version 1.6.2.3. Reverse and potential contaminant proteins were removed as well as proteins identified only by site and those that did not have at least one uniquely assigned peptide. For protein amounts in control conditions, iBAQ values were calculated using MaxQuant. For relative protein amounts, H/M and M/H ratios from MaxQuant were used. Two replicates—forward and reverse labeled—were analyzed.

### Gel filtration chromatography

Protein complexes in cytoplasmic HeLa lysate (Ipracell) were separated by size-exclusion chromatography using a HiPrep 16/60 Sephacryl S-500 HR column connected to an AKTApurifier protein purification system (GE Healthcare Life Sciences, Buckinghamshire, UK), essentially as described previously [81–83]. The column was eluted at 4 °C with 5% (w/v) sucrose, 0.1% (w/v) CHAPS, 20 mM HEPES NaOH, 5 mM DTT, and 150 mM NaCl, pH 7.0, at 0.15 ml/min and 2 ml fractions collected. The column was calibrated with protein standards (GE Healthcare Life Sciences) as shown in Additional file 1: Figure S3B.

### RIP-Seq

Immunoprecipitation was performed using a modified version of the method described previously [50]. This methodology involves a very short, 20-min immunoprecipitation to limit non-specific binding of mRNA to beads. This allowed us to isolate endogenous mRNA-protein complexes. HEK293 cells were harvested and lysed in lysis buffer (20 mM Tris pH 7.5, 200 mM NaCl, 5 mM MgCl_2_, 0.5% Triton-X100, 1× protease inhibitors (Roche), 1% BSA, 0.5 mM DTT, 5 mM NaF, 40 U/ml RiboLock (Thermo)). Lysates were spun down at 5000 rpm for 10 min, and supernatants used in subsequent steps. Aliquots were retained for total mRNA preparation. Protein G Dynabeads (Invitrogen) were pre-incubated with antibodies at a ratio of 1 μg antibody to 4.5 μl Dynabeads with rotation for 2.5 h in lysis buffer at 4C. For each 2 × 10^6^ cells, 8 μg of eIF4A1 antibody (ab31217), 4 μg of eIF4A2 antibody (ab31218), 4 μg of DDX6 antibody (ab70455), and 8 μg of rabbit IgG were used. Pre-incubated beads were washed 3 times with lysis buffer. Lysate was added to the washed beads and incubated at 4C with rotation for 20 min to minimize background. Beads were washed 3 times with lysis buffer. RNA from beads and total fractions was extracted using Trireagent (Invitrogen) followed by an additional acid phenol (Ambion) extraction. One microgram of RNA was subjected to library preparation using the TruSeq Stranded mRNA library preparation kit followed by NextSeq500 High Output 75 cycle sequencing. The RIP-Seq was performed in triplicate.

### Ribosome profiling sample and library preparation

The ribosome profiling methodology was adapted from the protocol from Ingolia 2012. 3 × 10 cm plates of HEK293 cells were used per condition. Medium was changed 1.5 h prior to treatment with cycloheximide (CHX) at 100 μg/ml at 37 °C for 3 min. Cells were then washed with PBS-CHX (100 μg/ml) and lysed in 400 μl lysis buffer (20 mM Tris-Cl pH 7.4, 150 mM NaCl, 5 mM MgCl_2_, 1 mM DTT and 100 μg/ml CHX, 25 U/ml Turbo DNase, 1% Triton X100). Prior to digestion, 50 μl lysate was retained for total RNA samples and extracted with Trizol. Four hundred sixty microliters of lysate was digested with 11.6 μl RNase I (Ambion) for 40 min at 22 °C 650 rpm. The digestions were stopped with 14.8 μl Superase.In (Invitrogen). Three hundred microliters of the digestion was used for sucrose cushions (900 μl 1 M sucrose in polysome buffer), spun for 4 h at 70,000 rpm 4 °C. Pellets were then resuspended in 700 μl Qiazol and extracted using the miRNeasy kit (Qiagen) following the manufacturer’s instructions and eluted in 2× 40 μl RNase-free water. Three hundred twenty microliters of TE buffer was added to each sample before loading on 100 k columns (Amicon), which were spun at 12,000 rpm for 12 min at 20 °C. The filtrate was retained and ethanol precipitated overnight at − 20 °C. This was repeated in triplicate followed by library preparation.

The RPF samples and markers at 500 nM (28 nt AGCGUGUACUCCGAAGAGGAUCCAACGU[phos], 34 nt AUGUACACGGAGUCGACCCAACGCGA[phos]) were run on a 15% TBE-Urea gel, stained with Sybr Gold (1 in 10,000), and imaged on a Typhoon Phospho Imager. The RPF sample region was extracted from the gel using the 28 nt and 34 nt oligos as markers (inclusive of 28 nt and exclusive of 34 nt). The gel piece was broken up, and the RPFs extracted in 400 μl RNA extraction buffer (300 mM NaOAc pH 5.5, 1 mM EDTA, 0.25% SDS) shaking overnight at 16 °C 550 rpm. The gel pieces were removed using Spin-X columns, and the RNA was isopropanol precipitated on dry ice. T4 PNK (NEB, M0201S) was used for 5′ phosphorylation and 3′ dephosphorylation at 37 °C for 1 h. Five microliters of 10 mM dATP was added, and the samples incubated at 37 °C for a further 30 min followed by 65 °C for 20 min. The sample was then precipitated with isopropanol on dry ice. For rRNA depletion, the RiboZero gold kit was used according to the manufacturer’s protocol with 10 μl rRNA depletion solution and ethanol precipitated at − 20 °C overnight. Ribosome-protected fragment libraries were prepared using Biooscientific Nextflex small RNA kit using 100 ng as input, 10 PCR cycles and with the gel extraction option. Total RNA libraries were prepared using the Biooscientific NEXTflex directional qRNA-Seq kits with 10 PCR cycles. The libraries were sequenced on NextSeq 75-cycle high output.

### RT-qPCR

For IP validations (Additional file 1: Figure S4A, S9A), RT-PCR was conducted on 50 ng of the RNA extracted from the IPs and the 10% input RNA using SuperScript III (Invitrogen). Primers were designed for RNAs found to be enriched in each of the IPs as well as RNAs enriched/depleted in all IPs (Additional file 2: Table S2). qPCR was conducted using Fast SYBR Green PCR Master Mix on a 7500 Fast Real Time PCR System (Applied Biosystems) with three technical replicates for two biological replicates. ΔCT of IP to input was used for relative quantification.

RT-qPCR for samples treated with 0.3 μM DMSO or 0.3 μM RocA (Additional file 1: Figure S8B) for 30 min prior to harvesting were conducted as described above. ΔCT of IP to input per condition and ΔΔCT RocA to DMSO were used for relative quantification of the change in enrichment in IP binding following RocA treatment.

For qPCR of gradient fractions (Additional file 1: Figure S10), RT-PCR was conducted on equal volumes of RNA from each gradient fraction with three technical replicates for two biological replicates. The proportion of the mRNA present in each fraction is plotted. Primers used are listed in Additional file 2: Table S2.

### siRNA knockdowns and RNA-Seq of total mRNA

HEK293 cells were plated at 10^6^ per 10 cm plate 24 h before transfection with 30 nM siRNA (control siRNA #3 from Dharmacon; specific siRNA from Ambion: CNOT1—ID no. S22844, TNRC6A—ID no. S26154, TNRC6B—ID no. S23060) and Dharmafect 1. Forty-eight hours after transfection, cells were harvested by scraping into ice cold PBS, spun down, and directly extracted in Trireagent (Invitrogen) followed by acid phenol (Ambion) extraction. Four micrograms of RNA was subjected to library preparation using the TruSeq Stranded mRNA library preparation kit followed by NextSeq500 High Output 75 cycle sequencing. The TNRC6A knockdowns were sequenced in duplicate, and CNOT1 knockdown sequencing performed in quadruplicate.

### Sucrose density gradient RNA-Seq and RT-qPCR

Cells were transfected as above. Forty-eight hours after transfection, cells were harvested by scraping into ice cold PBS and lysed in lysis buffer (15 mM TrisHCl (pH 7.4), 15 mM MgCl2, 0.15 M NaCl, 1% Triton X-100, 0.1 mg/ml cycloheximide, and 1 mg/ml heparin). The nuclei and debris were removed by centrifugation at 12,000×*g* for 5 min, and the supernatants were loaded onto 10–50% sucrose gradients and performed as described previously [84]. Subpolysomal and polysomal fractions were pooled, and alongside the input RNA, the purified RNA was subjected to 2.5 M LiCl precipitation at 4C overnight, followed by 20 min centrifugation at 12,000 rpm. The RNA was washed twice with 75% ethanol and resuspended in H_2_O. Four micrograms of RNA of total, subpolysomal, and polysomal RNA was subjected to library preparation using the TruSeq Stranded mRNA library preparation kit followed by NextSeq500 High Output 75 cycle sequencing. For puromycin treatment, cells were harvested as above, omitting cycloheximide, and treated with 100 μg/ml puromycin for 3 min prior to scraping into ice cold PBS. Control cells were treated with an equivalent amount of DMSO. Gradients and extraction were performed as above. Individual fractions for the puromycin-treated samples (Additional file 1: Figure S11B) and the control and CNOT1 siRNA experiments (Additional file 1: Figure S9B, S10) were collected, and RNA was prepared as above with LiCl precipitation. Equal volumes of each fraction (1 μl) were subjected to RT-qPCR, as described above. Relative amounts in each fraction were calculated by comparing to signal from all fractions. The experiments were performed in triplicate.

### Protein production and purification

cDNAs corresponding to eIF4A1, eIF4A2, and eIF4H were cloned into pET-SUMO vector and heterologously produced in *E. coli* BL21 (DE3) CodonPlus-RP as N-terminal SUMO-fusion proteins. Biomass was produced applying standard protocols for IPTG-induction. Cells were harvested, resuspended, and lysed in buffer A (20 mM Tris/HCl, pH 7.5, 1 M NaCl, 30 mM imidazole, and 10% (v/v) glycerol) supplemented with 1 mM PMSF and complete EDTA-free protease inhibitor cocktail (Roche). After centrifugation at 75,000*g* supernatant was filtered (0.45 μm) and applied to HisTrap (GE Healthcare) affinity chromatography. Bound protein was eluted with a linear imidazole gradient. Pooled fractions were diluted in buffer B (20 mM Tris/HCl, pH 7.5, 10% (v/v) glycerol, 0.1 mM EDTA) and incubated with SUMO-protease for 1 h at 8 °C for cleavage of the SUMO-tag. The protein solutions were further diluted with buffer B and applied to a ResourceQ (GE Healthcare) anion exchange chromatography. Bound protein was eluted with a linear KCl gradient. Pooled fractions were further purified by size exclusion chromatography using a Superdex 200 column equilibrated in storage buffer (20 mM Hepes/KOH, pH 7.5., 100 mM KCl, 0.1 mM EDTA, 10% (v/v) glycerol, 1 mM TCEP). Pooled fractions were concentrated, snap-frozen in liquid nitrogen, and stored at − 80 °C. Protein concentrations were calculated from the absorbance at 280 nm (A280) using extinction coefficients 34,630 M^-1^ cm^-1^ (eIF4A1) and 40,130 M^-1^ cm^-1^ (eIF4A2) obtained from ExPASy server. All protein preparation showed an A280/A260 ratio of ≥ 1.9 indicating negligible amounts of contaminations by nucleic acids and nucleotides.

### Fluorescence anisotropy assay

For RNA-binding studies, 10 nM FAM-labeled RNAs (Sigma) were incubated with proteins (0–40 μM) in binding buffer (BB, 20 mM Hepes/KOH, pH 7.5, 100 KCl, 1 mM MgCl_2_, 1 mM AMP-PNP, 1 mM TCEP, 0.1% DMSO) in the presence and absence of 50 μM silvestrol in 20 μl reactions for 60 min at 25 °C. For experiments, protein-RNA complexes were formed by incubation of 1 μM FAM-labeled RNA with 1 μM protein in BB in the presence or absence of 50 μM silvestrol. Strand release was induced by addition of 20-fold excess of unlabelled (AG)_10_-RNA. For dilution-induced strand release, protein-RNA complexes were pre-formed as described above, or with 10 nM FAM-labeled RNA and 1 or 3 μM protein in the presence or absence of 50 μM silvestrol, respectively. Strand release was induced by 1:1 dilution of the reactions with BB. Fluorescence anisotropy was measured using a Victor X5 (Perkin Elmer). Dissociation constants and half-lives were obtained from fitting the experimental data to the Hill- and single-exponential decay equation.

### Binding selectivity assay

To investigate selectivity in RNA-binding studies, 0–7 μM proteins were incubated simultaneously with 25 nM Dy780-(AG)_10_ ssRNA (IBA life science) and 1–50-fold molar excess of competitor ssRNAs (CAA)_6_CA or A_20_ in binding buffer in the presence or absence of 50 μM silvestrol in 20 μl reactions for 60 min at 25 °C. Samples were adjusted to 5% (v/v) glycerol, and protein-RNA complexes were resolved by electrophoresis on TB-acrylamide gels. After separation, gels were incubated for 5 min in 10% (v/v) acetic acid and bands corresponding to the labeled RNA visualized using an Odyssey scanner (Licor) and signals were quantified using ImageStudio (Licor). Dissociation constants were derived from fitting the fraction bound versus protein concentration to the Hill equation using Prism GraphPad.

### Informatic methods

All scripts used in the analyses are available upon request.

### RNA-Seq analysis

FASTQ files were aligned to the human genome (hg19) using TopHat2 [85]. Alignment files were then transformed into raw count data using htseq-count [86]. Differential expression was performed using EdgeR [87].

### Sucrose gradient NGS analysis

Differential expression analysis was conducted for CNOT1 knockdown compared to control for subpolysomal and polysomal fractions. Only genes significantly (FDR < 0.05) altered in both fractions were then used in plots in Fig. 5b. For polysome association, FPKM values from the control siRNA subpolysomal fraction were subtracted from those of the polysomal fraction to obtain a measure of mRNA distribution between these fractions (Fig. 2b, Additional file 1: Figure S4B). These were used for polysome association density plots.

### RIP-Seq enrichment analysis

Performed similarly to previous RIP-Seq studies [50, 88].

### Ribosome profiling data processing and analysis

For RPF samples, Cutadapt was used to remove adapter sequences then PCR duplicates were removed using cd-hit-dup. The unique molecular identifiers were then removed with Cutadapt. First, the reads were aligned to rRNA and tRNA sequences and then to the hg19 transcriptome using bowtie. The number of mapped reads 28–30 nt in length for each replicate was 23, 33, and 41 million. The positions of the reads were counted using a modified script from RiboCounts selecting for read lengths 28–30 nt, and the reads that showed periodicity were retained for downstream analysis.

For total RNA samples, Cutadapt was used to remove adapter sequences then PCR duplicates were removed using cd-hit-dup. The reads were aligned to the hg19 transcriptome using Hisat2 and read counts obtained using HTseq-count. The most abundant transcript for each gene was used in downstream analysis.

Custom R scripts were used for downstream analysis. To avoid bias due to multiple mRNA isoforms, the most abundant transcript in the total RNA samples was used as the representative transcript for each gene. Only transcripts with at least 25 RPF reads and CDS length greater than 300 nt were included in the analysis.

In Fig. 2c and Additional file 1: Figure S11A, for the ribosome occupancy plots, RPF read counts were normalized for library size and an offset of 13 applied to the 28–30 nt RPF fragments. Transcripts per million (TPM) was calculated for total RNA samples as a measure of mRNA abundance. RPF read counts at each position of the transcript were divided by the TPM of the transcript to account for the mRNA abundance. Plotted is the mean normalized RPF read counts at each codon position 75 codons into the CDS from the AUG and STOP codon.

In Fig. 2f, the ribosome occupancy for the first 50 nt and last 50 nt of 5′UTRs (5′UTR length greater than 100 nt) of the mRNAs was calculated in the same way described above for Fig. 2c but using RPFs from all frames.

### GO term enrichment analysis

Performed using the Gene Ontology enrichment tool [89] using hierarchical sorting and retaining the most relevant child terms with Fisher’s exact test, only terms with FDR < 0.05 were considered significant.

### Sequences used in mRNA feature analysis

Sequences used in the analysis were derived from RefSeq annotations based on gene ID [90]; only unique sequences were considered.

### GA-tetramer enrichment

5′UTR, CDS, and 3′UTR sequences were obtained from the RefSeq database based on gene ID. The non-overlapping occurrence of the polypurine motif was counted for each of the regions using eight of the most enriched purine-rich motifs identified using Bind-n-Seq in Iwasaki et al. [37] (AAGA|AGAA|GAAA|GAGA|AGAG|GGAA|AAAA|GAAG). Motif frequency is calculated to account for sequence length. *p* values were obtained using the dunnTest with Bonferroni’s correction, part of the FSA package in R studio. For positional calculations, the occurrence of the eight motifs above was calculated per base in the first and last 50 nt of the 5′UTR (Fig. 3d) and corrected for gene number in each group and enrichment was calculated compared to values for all mRNAs detected in the RIP-Seq experiment. Statistical significance was calculated as above. For the motif analysis, MEME was used for selective enrichment of motifs between eIF4A2- and eIF4A1-bound mRNAs. The MEME settings used were as follows: -rna –mod zoops –minw 6 –maxw 8 –objfun se.

### 3′UTR analysis

Pumilio binding sites in the 3′UTR were calculated using the regular expression TGTA(A|C|G|T)ATA [91]. For control 3′UTRs, mRNAs bound by DDX6, eIF4A2, or both proteins were excluded from the group of all detected mRNAs. Enrichment of conserved miRNA family targets was calculated using Fisher’s exact test on Targetscan7 target predictions conserved miRNA families for human mRNA, with PCT > 0.5 [92].

### Statistical methods

For luciferase assays, all data represent three biological repeats unless stated otherwise. Error bars represent standard deviation. Significance is determined using a *t* test (two-tailed, paired). Statistical significance in figures is as follows: *p < 0.05, **p < 0.01, ***p < 0.001, and n.s.—not significant.

### Structure superimposition

The human eIF4A1 sequence was mapped onto the yeast eIF4A structure (PDB: 2vso) using Pymol.

### Antibodies used for Western blotting

The antibodies used are as follows: eIF4A1 (Abcam ab31217 1:1000), eIF4A2 (Abcam ab31218 1:1000; Santa Cruz sc-137148 1:1000), eIF4A pan (Cell Signaling 2013 1:1000), DDX6 (Abcam ab70455 1:1000, ab54611 1:1000), CNOT1 (ATLAS HPA 046577 1:500), GAPVD1 (Sigma SAB 1401626), TRIM32 (Abcam ab96612 1:500), CLP1 (Sigma SAB 1407080), CNOT7 (Abcam, ab57095), eIF4GI (Cell Signaling 1:500), EDC3 (Bethyl A303-986A-T 1:1000), GAPDH (Protein technologies 60004-1-Ig 1:5000), TNRC6A (Novus Biologicals, NBP1-28751, 1:2000), Vinculin (Abcam, ab18058 1:1000), and Flag (Sigma, F1804 1:1000).

## Supplementary information

**Additional file 1.** Supplementary Figure S1-S11 and Supplemental References.

**Additional file 2.** Supplementary Tables S1-S2.

**Additional file 3.** Review history.

## Data Availability

The datasets generated and/or analyzed during the current study are available in the GEO and ProteomeXchange repository. RIP-Seq, TNRC6, and CNOT1 siRNA RNA-Seq experiments have been deposited under GSE94690 [93]. Ribosome profiling experiments have been deposited under GSE134517 [93]. SILAC data is deposited under PXD014764 [94]. IP mass spectrometry data is deposited under PXD015772 [94].

## References

[1] Merrick WC (2015). eIF4F: a retrospective. J Biol Chem.

[2] Rogers GW, Richter NJ, Merrick WC (1999). Biochemical and kinetic characterization of the RNA helicase activity of eukaryotic initiation factor 4A. J Biol Chem.

[3] Conroy SC, Dever TE, Owens CL, Merrick WC (1990). Characterization of the 46,000-dalton subunit of eIF-4F. Arch Biochem Biophys.

[4] Lu Wei-Ting, Wilczynska Anna, Smith Ewan, Bushell Martin (2014). The diverse roles of the eIF4A family: you are the company you keep. Biochemical Society Transactions.

[5] Watanabe R (2010). The eukaryotic initiation factor (eIF) 4G HEAT domain promotes translation re-initiation in yeast both dependent on and independent of eIF4A mRNA helicase. J Biol Chem.

[6] Hinnebusch AG, Lorsch JR. The mechanism of eukaryotic translation initiation: new insights and challenges. Cold Spring Harb Perspect Biol. 2012;4. https://www.ncbi.nlm.nih.gov/pubmed/22815232 10.1101/cshperspect.a011544 PMC3475172 22815232

[7] Sen ND, Zhou F, Ingolia NT, Hinnebusch AG (2015). Genome-wide analysis of translational efficiency reveals distinct but overlapping functions of yeast DEAD-box RNA helicases Ded1 and eIF4A. Genome Res.

[8] Behm-Ansmant I (2006). mRNA degradation by miRNAs and GW182 requires both CCR4:NOT deadenylase and DCP1:DCP2 decapping complexes. Genes Dev.

[9] Eulalio A (2009). Deadenylation is a widespread effect of miRNA regulation. RNA.

[10] Chen CY, Zheng D, Xia Z, Shyu AB (2009). Ago-TNRC6 triggers microRNA-mediated decay by promoting two deadenylation steps. Nat Struct Mol Biol.

[11] Meijer H. A., Kong Y. W., Lu W. T., Wilczynska A., Spriggs R. V., Robinson S. W., Godfrey J. D., Willis A. E., Bushell M. (2013). Translational Repression and eIF4A2 Activity Are Critical for MicroRNA-Mediated Gene Regulation. Science.

[12] Cooke A, Prigge A, Wickens M (2010). Translational repression by deadenylases. J Biol Chem.

[13] Fukaya T, Tomari Y (2011). PABP is not essential for microRNA-mediated translational repression and deadenylation in vitro. EMBO J.

[14] Mishima Y (2012). Translational inhibition by deadenylation-independent mechanisms is central to microRNA-mediated silencing in zebrafish. Proc Natl Acad Sci U S A.

[15] Wilczynska A, Bushell M (2014). The complexity of miRNA-mediated repression. Cell Death & Differentiation.

[16] Collart M, The A (2016). Ccr4-Not complex is a key regulator of eukaryotic gene expression. Wiley Interdiscip Rev RNA.

[17] Mathys Hansruedi, Basquin Jérôme, Ozgur Sevim, Czarnocki-Cieciura Mariusz, Bonneau Fabien, Aartse Aafke, Dziembowski Andrzej, Nowotny Marcin, Conti Elena, Filipowicz Witold (2014). Structural and Biochemical Insights to the Role of the CCR4-NOT Complex and DDX6 ATPase in MicroRNA Repression. Molecular Cell.

[18] Marintchev A (2009). Topology and regulation of the human eIF4A/4G/4H helicase complex in translation initiation. Cell.

[19] Minshall N, Kress M, Weil D, Standart N (2009). Role of p54 RNA helicase activity and its C-terminal domain in translational repression, P-body localization and assembly. Mol Biol Cell.

[20] Weston A, Sommerville J (2006). Xp54 and related (DDX6-like) RNA helicases: roles in messenger RNP assembly, translation regulation and RNA degradation. Nucleic Acids Res.

[21] Rissland OS (2017). The influence of microRNAs and poly(A) tail length on endogenous mRNA–protein complexes. Genome Biol.

[22] Yoder-Hill J, Pause A, Sonenberg N, Merrick WC (1993). The p46 subunit of eukaryotic initiation factor (eIF)-4F exchanges with eIF-4A. J Biol Chem.

[23] Meijer Hedda A, Schmidt Tobias, Gillen Sarah L, Langlais Claudia, Jukes-Jones Rebekah, de Moor Cornelia H, Cain Kelvin, Wilczynska Ania, Bushell Martin (2019). DEAD-box helicase eIF4A2 inhibits CNOT7 deadenylation activity. Nucleic Acids Research.

[24] Chen Y (2014). A DDX6-CNOT1 complex and W-binding pockets in CNOT9 reveal direct links between miRNA target recognition and silencing. Mol Cell.

[25] Kuzuoğlu-Öztürk D (2016). miRISC and the CCR4-NOT complex silence mRNA targets independently of 43S ribosomal scanning. EMBO J.

[26] Olsen PH, Ambros V (1999). The LIN-4 regulatory RNA controls developmental timing in Caenorhabditis elegans by blocking LIN-14 protein synthesis after the initiation of translation. Dev Biol.

[27] Petersen CP, Bordeleau ME, Pelletier J, Sharp PA (2006). Short RNAs repress translation after initiation in mammalian cells. Mol Cell.

[28] Maroney PA, Yu Y, Fisher J, Nilsen TW (2006). Evidence that microRNAs are associated with translating messenger RNAs in human cells. Nat Struct Mol Biol.

[29] Nottrott S, Simard MJ, Richter JD (2006). Human let-7a miRNA blocks protein production on actively translating polyribosomes. Nat Struct Mol Biol.

[30] Pillai RS (2005). Inhibition of translational initiation by Let-7 MicroRNA in human cells. Science.

[31] Mathonnet G (2007). MicroRNA inhibition of translation initiation in vitro by targeting the cap-binding complex eIF4F. Science.

[32] Bazzini AA, Lee MT, Giraldez AJ (2012). Ribosome profiling shows that miR-430 reduces translation before causing mRNA decay in zebrafish. Science.

[33] Thermann R, Hentze MW (2007). Drosophila miR2 induces pseudo-polysomes and inhibits translation initiation. Nature.

[34] Fukaya T, Iwakawa H, Tomari Y (2014). MicroRNAs block assembly of eIF4F translation initiation complex in Drosophila. Mol Cell.

[35] Fukao A (2014). MicroRNAs trigger dissociation of eIF4AI and eIF4AII from target mRNAs in humans. Mol Cell.

[36] Ricci EP (2013). miRNA repression of translation in vitro takes place during 43S ribosomal scanning. Nucleic Acids Res.

[37] Iwasaki S, Floor SN, Ingolia NT (2016). Rocaglates convert DEAD-box protein eIF4A into a sequence-selective translational repressor. Nature.

[38] Galicia-Vázquez G, Cencic R, Robert F, Agenor AQ, Pelletier J (2012). A cellular response linking eIF4AI activity to eIF4AII transcription. RNA.

[39] Galicia-Vázquez G (2014). Regulation of eukaryotic initiation factor 4AII by MyoD during murine myogenic cell differentiation. PLoS One.

[40] Svitkin YV, Pause A, Haghighat A, Pyronnet S (2001). The requirement for eukaryotic initiation factor 4A (elF4A) in translation is in direct proportion to the degree of mRNA 5 ’ secondary structure.

[41] Ishihama Y (2005). Exponentially modified protein abundance index (emPAI) for estimation of absolute protein amount in proteomics by the number of sequenced peptides per protein. Mol Cell Proteomics.

[42] Schutz P (2008). Crystal structure of the yeast eIF4A-eIF4G complex: an RNA-helicase controlled by protein-protein interactions. Proc Natl Acad Sci U S A.

[43] Schwamborn JC, Berezikov E, Knoblich JA (2009). The TRIM-NHL protein TRIM32 activates microRNAs and prevents self-renewal in mouse neural progenitors. Cell.

[44] Nicklas S (2015). The RNA helicase DDX6 regulates cell-fate specification in neural stem cells via miRNAs. Nucleic Acids Res.

[45] Golden Ryan J., Chen Beibei, Li Tuo, Braun Juliane, Manjunath Hema, Chen Xiang, Wu Jiaxi, Schmid Vanessa, Chang Tsung-Cheng, Kopp Florian, Ramirez-Martinez Andres, Tagliabracci Vincent S., Chen Zhijian J., Xie Yang, Mendell Joshua T. (2017). An Argonaute phosphorylation cycle promotes microRNA-mediated silencing. Nature.

[46] Salzman DW (2016). miR-34 activity is modulated through 5′-end phosphorylation in response to DNA damage. Nat Commun.

[47] Lau N-C (2009). Human Ccr4-Not complexes contain variable deadenylase subunits. Biochem J.

[48] Schwanhäusser B (2011). Global quantification of mammalian gene expression control. Nature.

[49] Lee S (2012). Global mapping of translation initiation sites in mammalian cells at single-nucleotide resolution. Proc Natl Acad Sci.

[50] Costello J (2015). Global mRNA selection mechanisms for translation initiation. Genome Biol.

[51] Dana A, Tuller T (2012). Determinants of translation elongation speed and ribosomal profiling biases in mouse embryonic stem cells. PLoS Comput Biol.

[52] Lian X (2016). Genome-wide and experimental resolution of relative translation elongation speed at individual gene level in human cells. PLoS Genet.

[53] Modelska A (2015). The malignant phenotype in breast cancer is driven by eIF4A1-mediated changes in the translational landscape. Cell Death Dis.

[54] Wolfe AL (2014). RNA G-quadruplexes cause eIF4A-dependent oncogene translation in cancer. Nature.

[55] Rubio CA (2014). Transcriptome-wide characterization of the eIF4A signature highlights plasticity in translation regulation. Genome Biol.

[56] Raza F (2015). Translational dysregulation in cancer: eIF4A isoforms and sequence determinants of eIF4A dependence. Biochem Soc Trans.

[57] Bailey TL (2009). MEME SUITE: tools for motif discovery and searching. Nucleic Acids Res.

[58] Chu Jennifer, Pelletier Jerry (2015). Targeting the eIF4A RNA helicase as an anti-neoplastic approach. Biochimica et Biophysica Acta (BBA) - Gene Regulatory Mechanisms.

[59] Rogers GW, Richter NJ, Lima WF, Merrick WC (2001). Modulation of the helicase activity of eIF4A by eIF4B, eIF4H, and eIF4F. J Biol Chem.

[60] Wilczynska A, Bushell M (2014). The complexity of miRNA-mediated repression. Cell Death Differ.

[61] Friedman RC, Farh KK-H, Burge CB, Bartel DP (2009). Most mammalian mRNAs are conserved targets of microRNAs. Genome Res.

[62] Huntzinger Eric, Braun Joerg E, Heimstädt Susanne, Zekri Latifa, Izaurralde Elisa (2010). Two PABPC1-binding sites in GW182 proteins promote miRNA-mediated gene silencing. The EMBO Journal.

[63] Keene JD (2007). RNA regulons: coordination of post-transcriptional events. Nat Rev Genet.

[64] Subtelny AO, Eichhorn SW, Chen GR, Sive H, Bartel DP (2014). Poly(a)-tail profiling reveals an embryonic switch in translational control. Nature.

[65] Ahmed CS, Winlow PL, Parsons AL, Jopling CL (2018). Eukaryotic translation initiation factor 4AII contributes to microRNA-122 regulation of hepatitis C virus replication. Nucleic Acids Res.

[66] Robert F (2014). Translation initiation factor eIF4F modifies the dexamethasone response in multiple myeloma. Proc Natl Acad Sci U S A.

[67] Schweingruber C, Soffientini P, Ruepp M-D, Bachi A, Mühlemann O (2016). Identification of interactions in the NMD complex using proximity-dependent biotinylation (BioID). PLoS One.

[68] Galicia-Vázquez G, Chu J, Pelletier J (2015). eIF4AII is dispensable for miRNA-mediated gene silencing. RNA.

[69] Linder P, Jankowsky E (2011). From unwinding to clamping—the DEAD box RNA helicase family. Nat Rev Mol Cell Biol.

[70] Ballut L (2005). The exon junction core complex is locked onto RNA by inhibition of eIF4AIII ATPase activity. Nat Struct Mol Biol.

[71] Saulière J (2012). CLIP-seq of eIF4AIII reveals transcriptome-wide mapping of the human exon junction complex. Nat Struct Mol Biol.

[72] Hubstenberger A (2017). P-body purification reveals the condensation of repressed mRNA regulons. Mol Cell.

[73] Radhakrishnan A (2016). The DEAD-box protein Dhh1p couples mRNA decay and translation by monitoring codon optimality. Cell.

[74] Oertlin C (2019). Generally applicable transcriptome-wide analysis of translation using anota2seq. Nucleic Acids Res.

[75] Dickens LS (2012). A death effector domain chain DISC model reveals a crucial role for caspase-8 chain assembly in mediating apoptotic cell death. Mol Cell.

[76] Craxton A (2015). XLS (c9orf142) is a new component of mammalian DNA double-stranded break repair. Cell Death Differ.

[77] Hughes MA (2016). Co-operative and hierarchical binding of c-FLIP and Caspase-8: a unified model defines how c-FLIP isoforms differentially control cell fate. Mol Cell.

[78] Cox J, Mann M (2008). MaxQuant enables high peptide identification rates, individualized p.p.b.-range mass accuracies and proteome-wide protein quantification. Nat Biotechnol.

[79] Cox J (2011). Andromeda: a peptide search engine integrated into the MaxQuant environment. J Proteome Res.

[80] Tyanova S (2016). The Perseus computational platform for comprehensive analysis of (prote)omics data. Nat Methods.

[81] Cain K (2000). Apaf-1 oligomerizes into biologically active approximately 700-kDa and inactive approximately 1.4-MDa apoptosome complexes. J Biol Chem.

[82] Feoktistova M (2011). cIAPs block ripoptosome formation, a RIP1/caspase-8 containing intracellular cell death complex differentially regulated by cFLIP isoforms. Mol Cell.

[83] Langlais C, Hughes MA, Cain K, MacFarlane M (2015). Biochemical analysis of initiator caspase-activating complexes: the apoptosome and the death-inducing signaling complex. Cold Spring Harb. Protoc.

[84] Kong YW (2008). The mechanism of micro-RNA-mediated translation repression is determined by the promoter of the target gene. Proc Natl Acad Sci U S A.

[85] Kim D (2013). TopHat2: accurate alignment of transcriptomes in the presence of insertions, deletions and gene fusions. Genome Biol.

[86] Anders S, Pyl PT, Huber W (2015). HTSeq--a Python framework to work with high-throughput sequencing data. Bioinformatics.

[87] Robinson MD, McCarthy DJ, Smyth GK (2010). edgeR: a bioconductor package for differential expression analysis of digital gene expression data. Bioinformatics.

[88] Castelli LM (2015). The 4E-BP Caf20p mediates both eIF4E-dependent and independent repression of translation. PLoS Genet.

[89] Alexa A, Rahnenfuhrer J (2010). topGO: topGO: enrichment analysis for Gene Ontology.

[90] Pruitt KD (2014). RefSeq: an update on mammalian reference sequences. Nucleic Acids Res.

[91] Gerber AP, Luschnig S, Krasnow MA, Brown PO, Herschlag D (2006). Genome-wide identification of mRNAs associated with the translational regulator PUMILIO in Drosophila melanogaster. Proc Natl Acad Sci U S A.

[92] Agarwal V, Bell GW, Nam J-W, Bartel DP. Predicting effective microRNA target sites in mammalian mRNAs. Elife. 2015;4:e05005. 10.7554/eLife.05005 10.7554/eLife.05005 PMC4532895 26267216

[93] Wilczynska A, Gillen SL, Schmidt T, Meijer HA, Jukes-Jones R, Langlais C, Kopra K, Lu WT, Godfrey JD, Hawley BR, Hodge K, Zanivan SR, Cain K, Le Quesne J, Bushell M. eIF4A2 drives repression of translation at initiation by Ccr4-Not through purine-rich motifs in the 5’UTR. Gene Expr Omnibus. 2019; https://www.ncbi.nlm.nih.gov/geo/query/acc.cgi?acc=GSE94690. 10.1186/s13059-019-1857-2 PMC6886185 31791371

[94] Wilczynska A, Gillen SL, Schmidt T, Meijer HA, Jukes-Jones R, Langlais C, Kopra K, Lu WT, Godfrey JD, Hawley BR, Hodge K, Zanivan SR, Cain K, Le Quesne J, Bushell M. eIF4A2 drives repression of translation at initiation by Ccr4-Not through purine-rich motifs in the 5’UTR. PRIDE Archive. 2019; https://www.ebi.ac.uk/pride/archive/projects/PXD014764. 10.1186/s13059-019-1857-2 PMC6886185 31791371

