# Supplementary material for: eIF4A2 drives repression of translation at initiation by Ccr4-Not through purine-rich motifs in the 5′UTR
Source: Genome Biol. 2019 Dec 2;20:262. doi: 10.1186/s13059-019-1857-2 (PMC6886185; doi:10.1186/s13059-019-1857-2)
Supplement: Supplementary file 1 — Additional file 1. Supplementary Figure S1-S11 and Supplemental References. [file 13059_2019_1857_MOESM1_ESM.pdf]

Fig. S1.

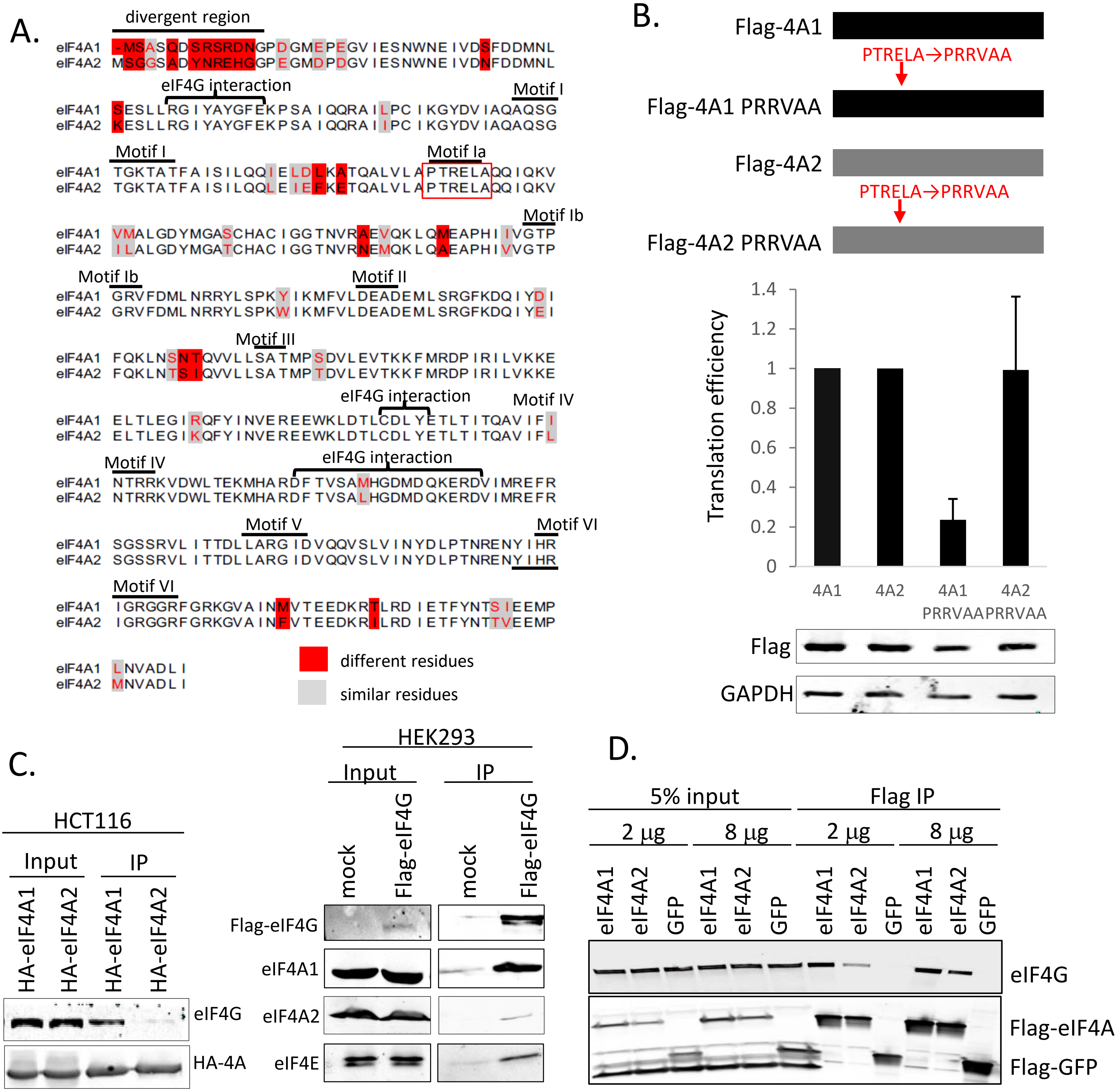

Fig. S1. eIF4A2 is not a core component of the eIF4F complex. A. Alignment of human eIF4A1 and eIF4A2 protein sequence. Different and similar residues are highlighted in red and grey, respectively. The remaining residues are identical. Sequence motifs and eIF4G interaction sites have been marked, as in Lu et al.[1]. The divergent region deleted in Fig. S2A is marked, the PTRELA motif mutated in dominant negative constructs is marked with a red box. **B.** PRRVAA mutant of eIF4A2 does not have dominant negative activity on translation. HEK293 cells were transfected with pRL-SV40 reporter plasmid together with plasmids encoding eIF4A constructs as shown in the schematics (top). Translational efficiency denotes luciferase activity compared to reporter RNA abundance measured by qPCR. Graph shows an average of 3 experiments. Western blots show overexpressed proteins (Flag) and control (GAPDH) from a representative experiment. **C.** eIF4G is not the principle protein partner of eIF4A2. Immunoprecipitations using anti-Flag or anti-HA beads were performed on lysates from two different cell lines (HCT116 and HEK293) expressing tagged versions of either eIF4A paralogs or eIF4G. The HA-tag is N-terminal, while the previously used Flag-tagged eIF4A1 and eIF4A2 constructs were C-terminal [2]. Interactions were confirmed by Western blot. Input represents 10% of lysate. mock = mock transfected. Figure presents representative blots. **D.** Relative stoichiometry of overexpression of Flag-tagged eIF4A1 and eIF4A2 is critical for observation of the preferential interaction of eIF4G with eIF4A1. Immunoprecipitations using anti-Flag beads were performed on lysates from HEK293 cells transfected with either 2 µg or 8 µg of Flag-4A plasmid per 10 cm plate. Western blots revealed that high levels of overexpression of eIF4A2 (8 µg transfection) resulted in a disproportional increase of pulled down eIF4G compared to eIF4A1.

Fig. S2.

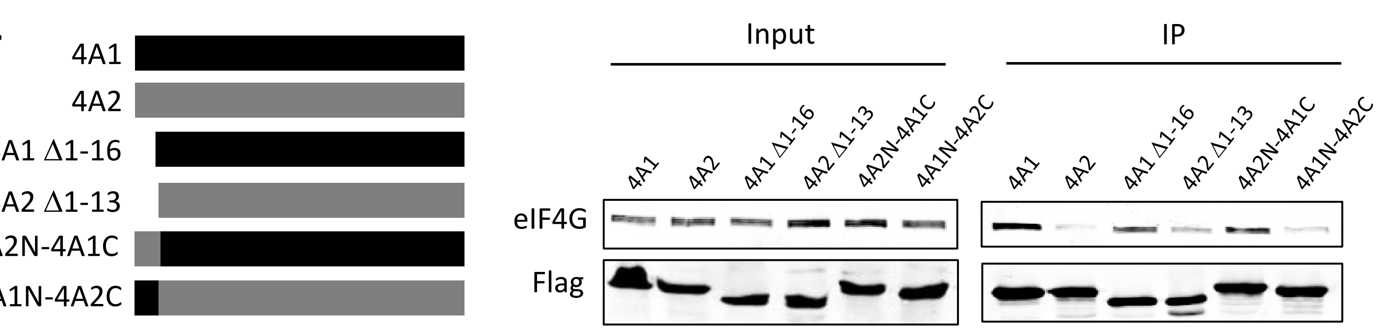

B.

```
                                                           34      41          101  143    150      207 208
hs_eIF4A2   --------MS GGSADYNREH GGPEGMDPDG VIESNWNEIV DNFDDMNLKE...QQLEIEFKET...NEMQKLQAEA...KLNTSIQVVL
Mm_eIF4A2   --------MS GGSADYNREH GGPEGMDPDG VIESNWNEIV DNFDDMNLKE...QQLEIEFKET...NEMQKLQAEA...KLNTSIQVVL
Gg_eIF4A2   --------MS GGSADYSRDH GGPEGMEPDG VIESNWNEIV DNFDDMNLKE...QQLEIDLKET...NEMQKLQAEA...KLSTNIQVVL
Dm_eIF4A    ---------- -MDDRNEIPQ DGPASMEPEG VIESTWHEVY DNFDDMNLRE...QQIDTSIREC...EDARILESG-...MLPPDVQVIL
Anopheles   ---------- MDDNRQEQTY DGPAGMNPDG IIESNYSESY DNFDQMGLRE...QQIKTEIPDC...DDMRRLEQG-...KLPADVQVIL
Ce_eIF4A    ---------- --MTDVKNDV NVSSVVDADG LIEGNYDQVV ESFDDMELKE...QRIDHEDPHV...DDQRKLEAG-...SMPQDVQVVL
At_eIF4A2   MAGSAPEGTQ FDTRQFDQRL NEVLDGQD-- EFFTSYDEVH ESFDAMGLQE...QQLDYALLQC...EDQRILQAG-...LLPPKIQVGV
At_eIF4A1   MAGSAPEGTQ FDARQFDQKL NEVLEGQD-- EFFTSYDDVH ESFDAMGLQE...QQLDFSLIQC...EDQRILQAG-...LLPPKIQVGV
Os_Os06g07  MAGMAPEGSQ FDAKHYDSKM QELLNQGETE EFFTSYDEVH ESFDDMGLQE...QQLDYAVVEC...EDQRILASG-...LLPSKIQVGV
Os_Os02g01  MAGMAPEGSQ FDAKHYDSKM QELLHQGDNE EFFTSYDEVF ESFDDMGLQE...QQLDYGLVEC...EDQRILASG-...LLPPKIQVGV
hs_eIF4A1   ---------M SASQDSRSRD NGPDGMEPEG VIESNWNEIV DSFDDMNLSE...QQIELDLKAT...AEVQKLQMEA...KLNSNTQVVL
Mm_eIF4A1   ---------M SASQDSRSRD NGPDGMEPEG VIESNWNEIV DSFDDMNLSE...QQIELDLKAT...AEVQKLQMEA...KLNSNTQVVL
Dr_eIF4A2   ---------M SSEHEDRPRD NGPEGMEPDG VIESNWKEIV DSFDDMNLRE...QQIDVELKAT...NDVQKLQADV...KLATDTQVIL
Dr__eIF4A1  ---------M SADYEGRPRD NGPEGMEPDG VIESNWDEIV DSFDDMNLRE...QQIDIELKGT...NEVQKLQAEA...KLSTSIQVVL
Sc__eIF4A   ---------- ---------M SEGITDIEES QIQTNYDKVV YKFDDMELDE...QRIDTSVKAP...EDAEGLRD--...LLPPTTQVVL
Eremotheci  ---------- ---------M SDSITNPENS EIQTNYDKIV HKFDELKLKE...QRIDESIKAP...EDAEALRAG-...MLPPTTQVVL
Sp_eIF4A    ---------- ---------- --MVDQLEDS VIETNYDEVI DTFDDMNLKP...QKIDTSLKQC...DDMAALQAG-...LLPPTAQVVL
Magnaporth  ---------- --------MA DKGLEDVPEG QIESNYDETV DSFDEMNLKS...QKIDTNVKQC...DDMKALQDG-...LLPQSTQVVL
Neurospora  ---------- -------MAT DKGLEDIPEG QIESNYDETV DSFDEMNLKP...QKIDPSLKAC...DDMKALQDG-...LLPQSTQVVL
Xt_eIF4A1   ---------M SASYESRPRD NGPEGMEPDG VIESNWNEVV DSFDDMSLSE...QQIELDMKAT...AEVQKLQSEA...KLSSNAQVVL
```

C.

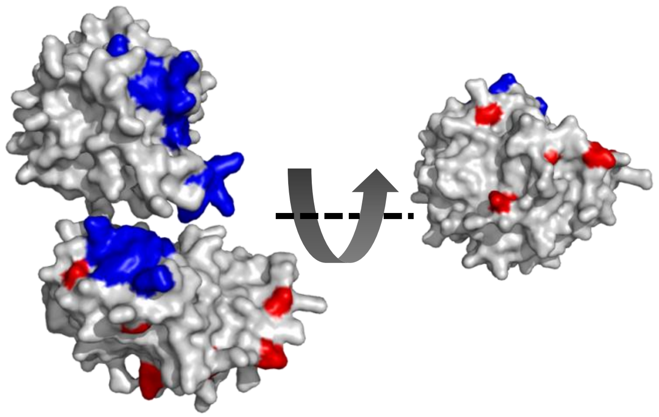

Fig. S2. Amino acids responsible for differential function of eIF4A1 and eIF4A2. A. The N-terminal region of eIF4A paralogs is not responsible for differential eIF4G binding. Immunoprecipitations using anti-Flag beads were performed on lysates from HEK293 cells expressing Flag-tagged constructs shown in schematics (left). Western blots confirm that truncation or exchange of N-termini between the proteins does not affect the relative interactions with eIF4G. Input represents 10% of lysate used in IP. **B.** Fragments of Clustal Omega alignments showing conservation of amino acids between eIF4A orthologues from very diverse organisms, including plants. Numbers on top denote amino acid positions within human eIF4A2 that were mutated in constructs used in Fig. 1AB. Red highlighting shows amino acid conservation of eIF4A2-like amino acids, while blue highlighting represents eIF4A1-like amino acid conservation. NCBI accession numbers: At_eIF4A2 - *Arabidopsis thaliana*, 841868; At_eIF4A1 - *Arabidopsis thaliana*, 820605; Anopheles - *Anopheles gambiae* str. PEST, 1279280; Ce_eIF4A - *Caenorhabditis elegans*, 175966; Dm_eIF4A - *Drosophila melanogaster*, 33835; Dr__eIF4A1 – *Danio rerio*, 386634; Dr_eIF4A2 - *Danio rerio*, 406760; Eremotheci - *Eremothecium gossypii*, 4619790; Gg_eIF4A2 - *Gallus gallus*, 395232; hs_eIF4A1 - *homo sapiens*, 1973; hs_eIF4A2 - *homo sapiens*, 1974; Magnaporth - *Magnaporthe oryzae, 2675387;* Mm_eIF4A1 - *Mus musculus*, 13681; Mm_eIF4A2 - *Mus musculus*, 13682; Neurospora - *Neurospora crassa*, 3874573; Os_Os06g07 - *Oryza sativa*, 4341966; Os_Os02g01 - *Oryza sativa*, 4328286; Sc__eIF4A - *Saccharomyces cerevisiae*, 853933; Sp_eIF4A – *Schizosaccharomyces pombe*, 2542948; Xt_eIF4A1 – *Xenopus tropicalis*, 496556. **C.** Position of the 7xMUT amino acids (as in Fig. 1A) (red) mapped onto yeast eIF4A structure (PDB: 2vso). eIF4G binding surfaces are marked in blue.

Fig. S3.

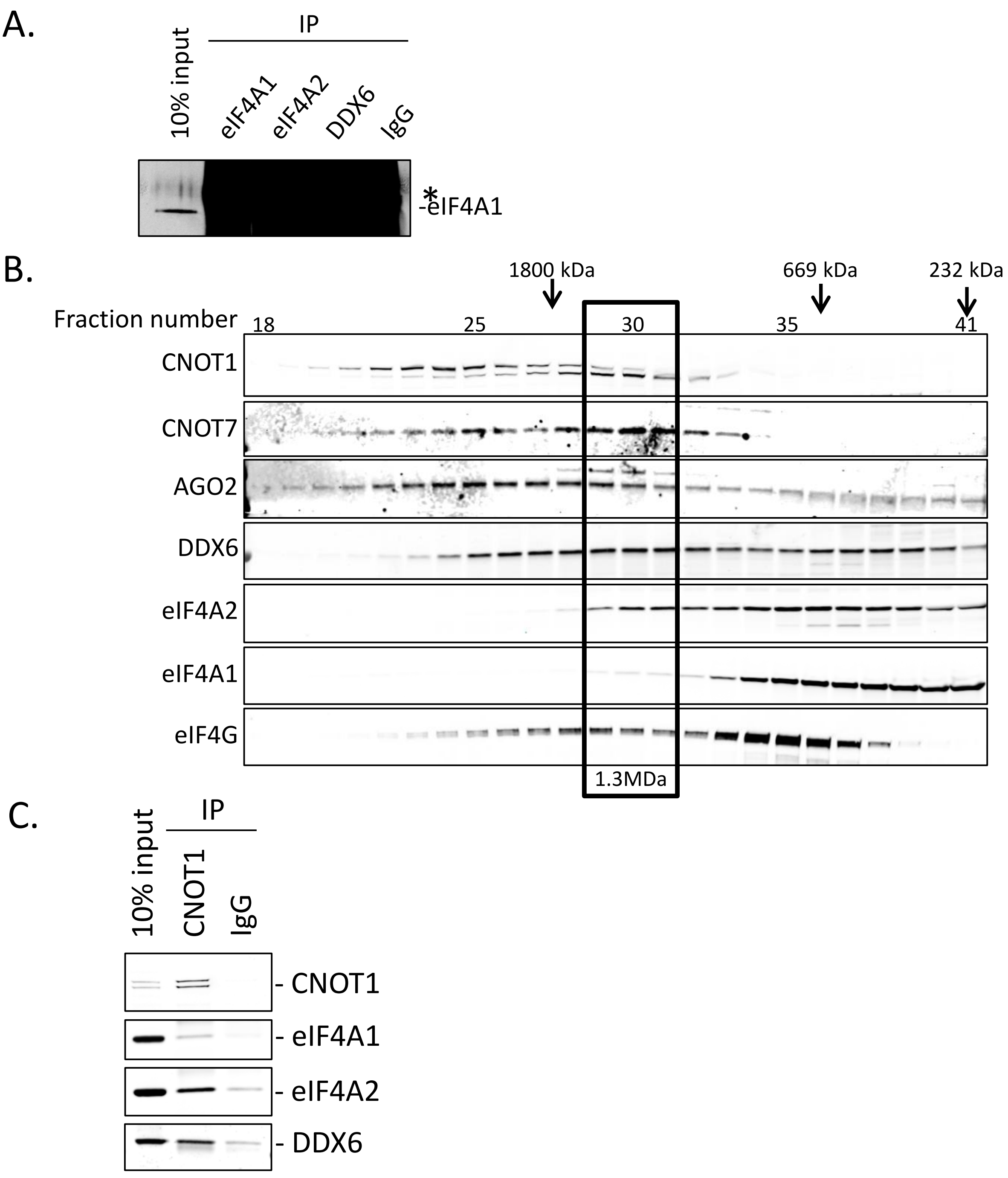

Fig. S3. Gel filtration identifies fractions containing CNOT1 and eIF4A2 and interactions of eIF4A2 and DDX6 with CNOT1 in total lysate. A. Overexposed image of blot shown in Fig. 1F shows eIF4A1 input. **B.** HeLa cytoplasmic lysate was subjected to gel filtration chromatography on a Sephacryl S-500 HR column, calibrated with protein standards denoted by arrows (1800 kDa – GE Standard 1; 669 kDa – thyroglobulin; 232 kDa – catalase). Fractions 29-31 contain both CNOT1 and eIF4A2 and migrate at a size of about 1.3 MDa, as assessed by comparison protein standard migration. These fractions were subsequently used for immunoprecipitations shown in Fig. 1F. **C.** CNOT1 was immunoprecipitated from total cytoplasmic HeLa lysate and Western blotting revealed robust interactions with both eIF4A2 and DDX6.

Fig. S4.

A.

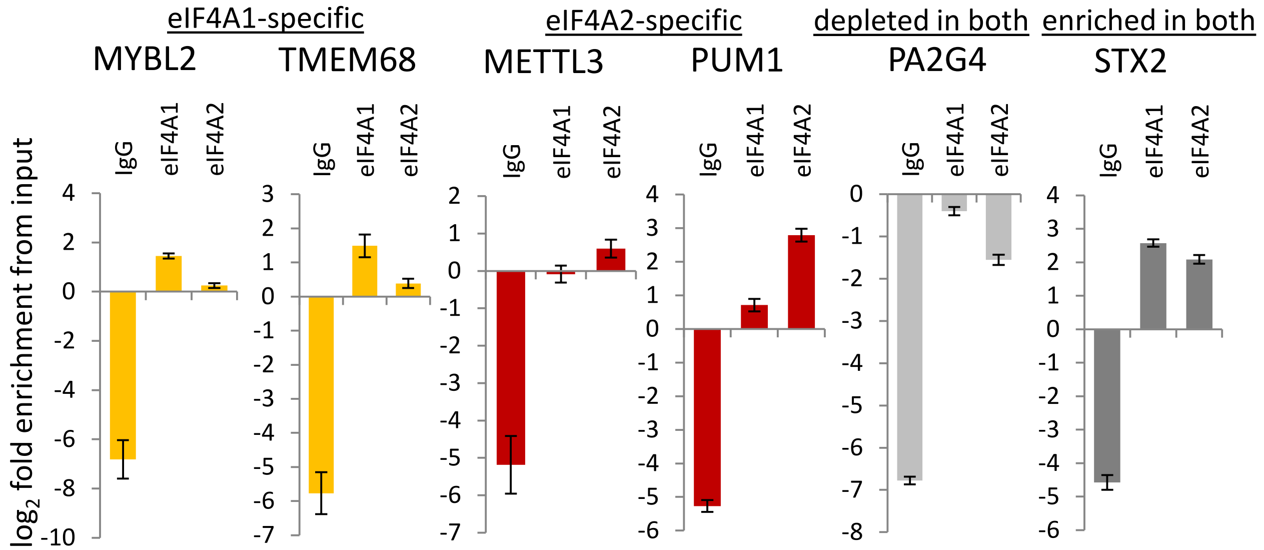

Enrichment in RIP-seq:	eIF4A1: 0.420<br>eIF4A2: -0.364	eIF4A1: 0.509<br>eIF4A2:0.154	eIF4A1: -0.376<br>eIF4A2: 0.318	eIF4A1: -0.066<br>eIF4A2: 0.746	eIF4A1: -1.726<br>eIF4A2: -1.743	eIF4A1: 1.640<br>eIF4A2: 1.596
FDR in RIP-seq:	0.046<br>0.09 (n.s.)	3.38×10^{-5}<br>0.238 (n.s.)	0.018<br>0.047	0.746<br>5.50×10^{-5}	3.32×10^{-36}<br>5.96×10^{-37}	9.69×10^{-39}<br>6.34×10^{-37}

B.

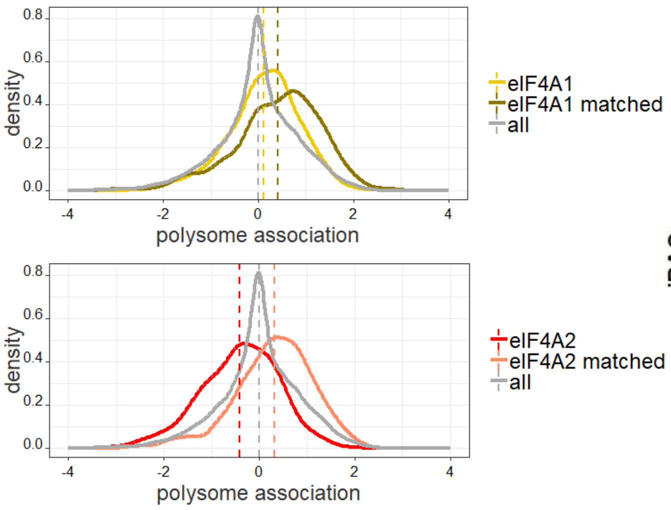

C.

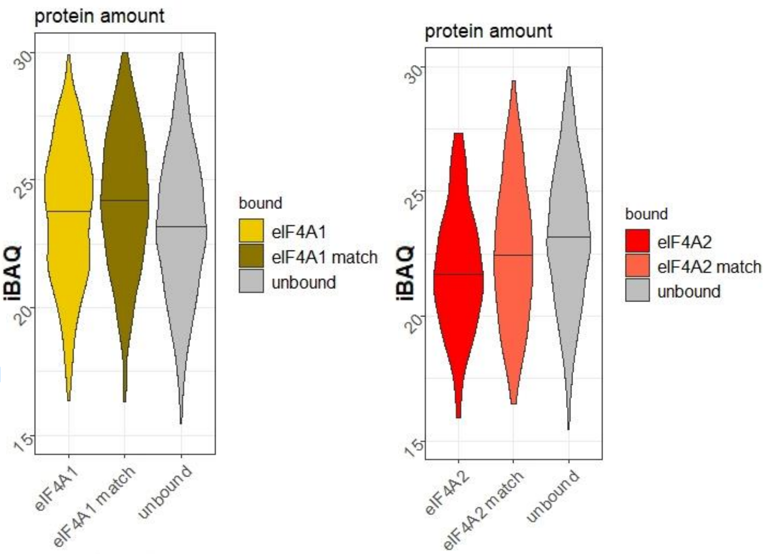

D.

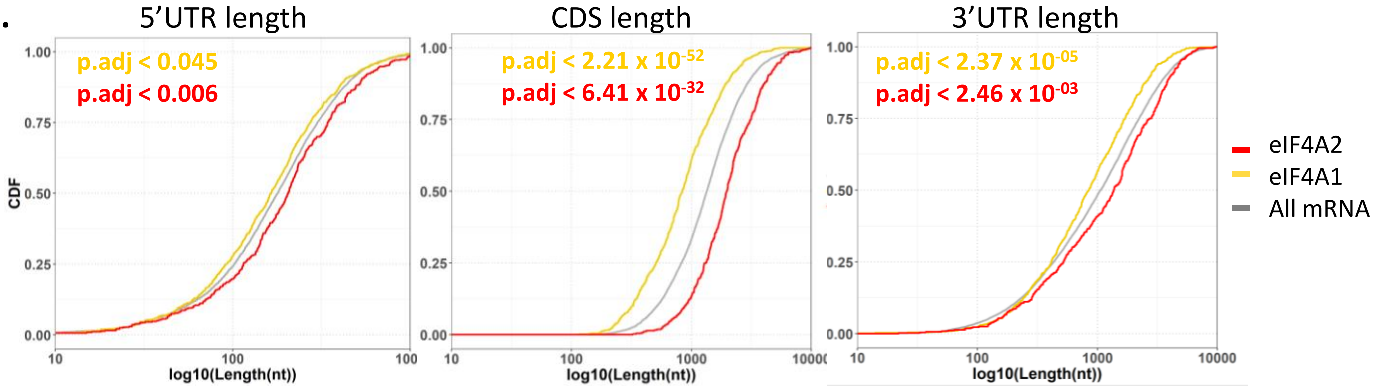

Fig.S4. A. RIP-Seq reveals differential binding of helicases to mRNAs. Validation of enrichment of mRNAs in IPs of the eIF4A paralogs as in Fig. 2A. Bar charts show RT-qPCR validation of several genes from the IPs in independent experiments (n=2). Recovery in IPs is calculated relative to input, error bars represent confidence intervals. The table underneath shows log_2-fold changes and FDR values of enrichment from RIP-Seq experiments as in Fig. 2A. (not significant – n.s. – results in gray). **B. Polysome distribution of length-matched mRNAs.** Differential association with polysomes as in Fig. 2B for eIF4A1 and eIF4A2 compared to length-matched mRNAs. **C. Protein production from different groups of mRNAs.** iBAQ quantitation of protein abundance in control conditions in pulsed-SILAC as in Fig. 2D but with the addition of length-matched groups as in (**B**). **D. UTR and CDS length of DEAD-box protein bound mRNAs.** eIF4A2-bound mRNAs have significantly longer CDSes, and eIF4A1-bound mRNAs have shorter CDSes than the average mRNA.

Fig. S5.

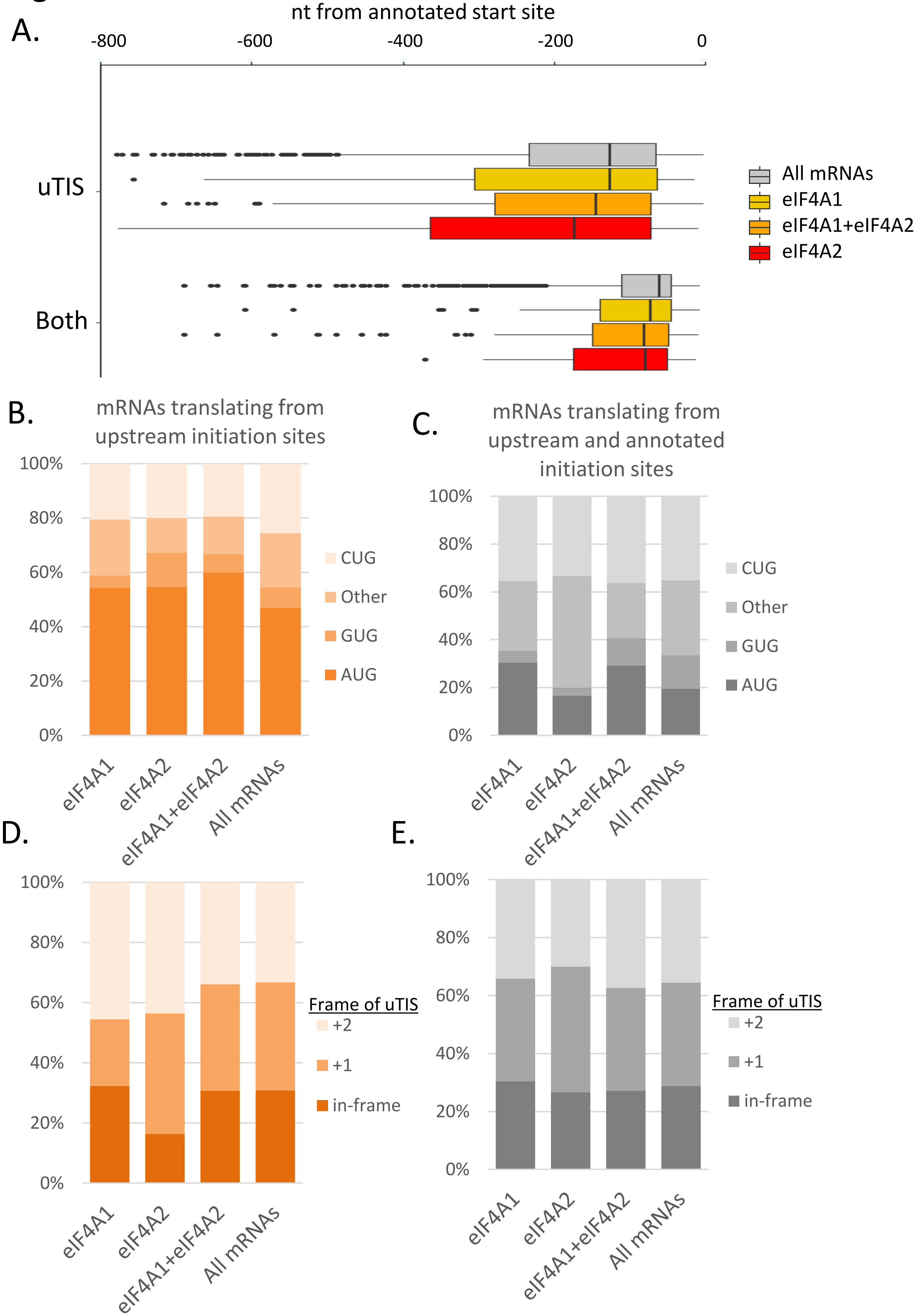

Fig. S5.Characterisation of uORFs. A. For mRNAs only translating from upstream translation initiation sites (uTIS), the uTIS is further away from the annotated start site than for those translating from both initiation sites. **B&C.** The start codon of the upstream translation initiation site (where there are multiple per transcript the most utilised site, as determined by the GTI-seq data, is used in the analysis) for the mRNAs only translating from upstream translation initiation sites (**B**) and mRNAs translating both from upstream sites and the annotated start site (**C**). **D&E.** The frame of the most utilised upstream start site in relation to the annotated start site of the transcript for mRNAs only translating from upstream translation initiation sites (**D**) and mRNAs translating both from upstream sites and the annotated start site (**E**).

Fig. S6.

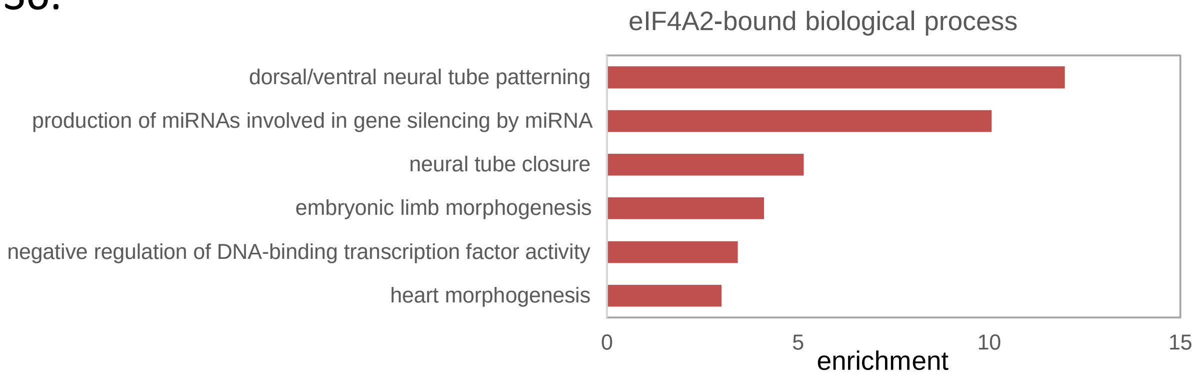

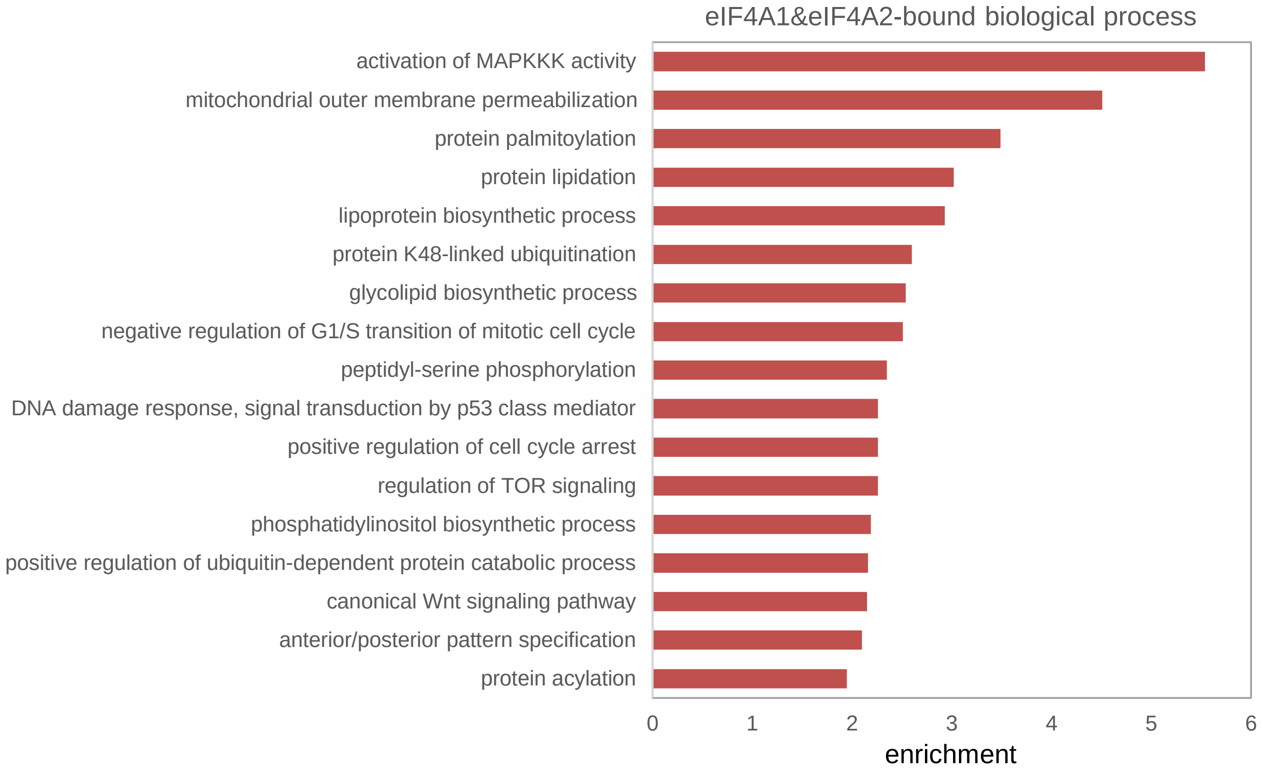

Fig. S6. eIF4A2 binds mRNAs encoding for proteins in specific functional groups. Top GO terms enriched in groups of mRNAs bound by the eIF4A1 and eIF4A2 together and eIF4A2 alone. Graphs show enrichment for GO terms with FDR<0.05.

Fig. S7.

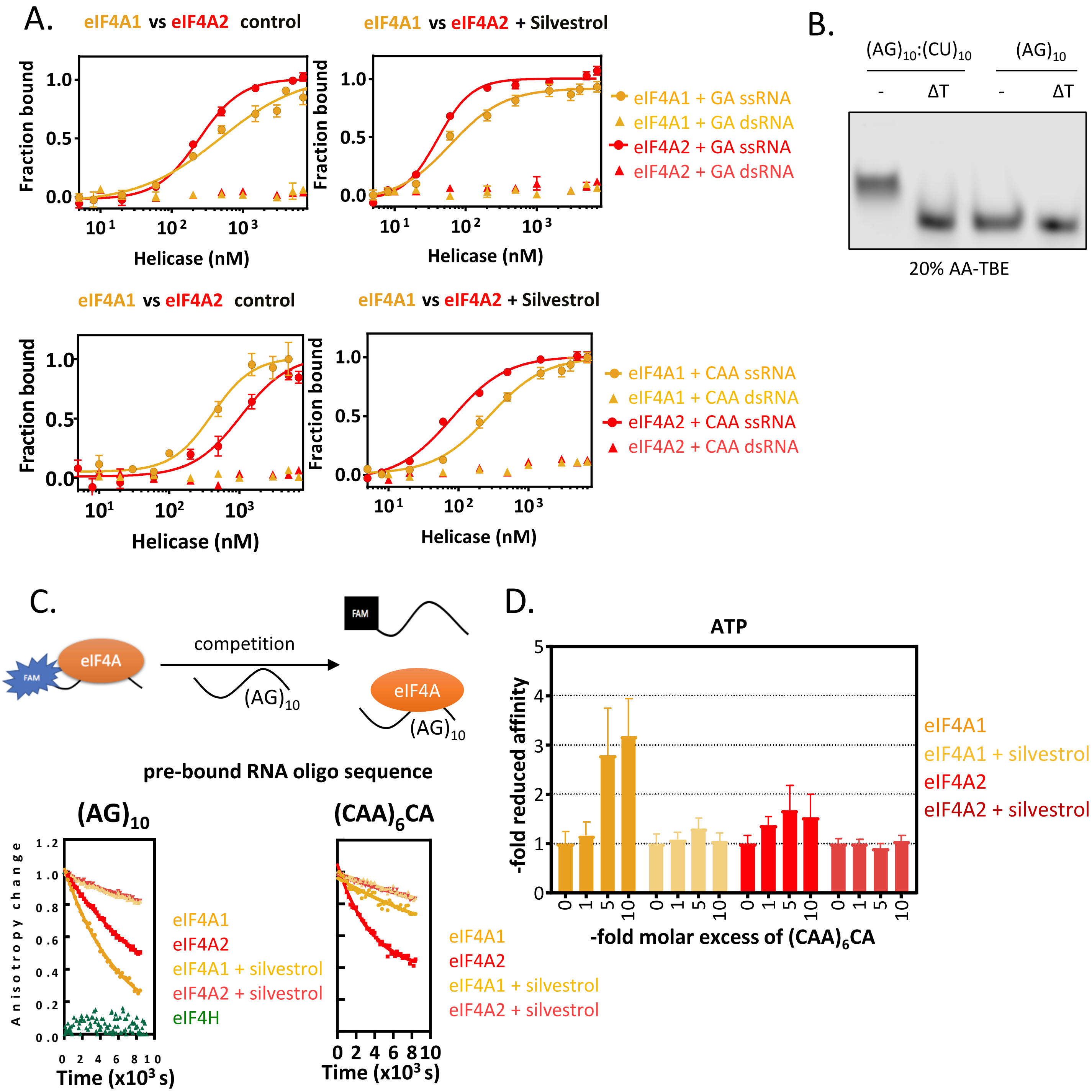

Fig. S7. A. eIF4A1 and eIF4A2 do not bind to dsRNA. Protein-RNA interactions between eIF4A1 (gold) and eIF4A2 (red) with FAM-labelled GA ssRNA [$(AG)_{10}$], GA dsRNA [$(AG)_{10}$:$(CU)_{10}$], CAA ssRNA [$(CAA)_6CA$], and CAA dsRNA [$(CAA)_6CA$:$UG(UUG)_6$] in the absence (left panels) and presence (right panels) of 50 µM Silvestrol measured by fluorescence anisotropy. Data represent mean ± SD, n = 3. **B. The purine-only ssRNA oligos show no secondary structure**. Analysis of 0.2 pmol Dy780-$(AG)_{10}$ and dsRNA version Dy780-$(AG)_{10}$:$(CU)_{10}$ before and after heat-treatment resolved on a 20% polyacrylamide TBE gel. Gel was scanned with Licor Odyssey and RNA bands visualised using the 800 nm channel. **C.** Schematic representation of the performed clamping experiments**. The pre-formed eIF4A-FAM-RNA complex displays high fluorescence anisotropy.** Strand release is induced by addition of excess amounts of unlabelled competitor RNA resulting in displacement of the FAM-labelled RNA strand from the protein which lowers fluorescence anisotropy. Each clamping experiment was started from pre-formed eIF4A1- (gold) and eIF4A2-FAM-RNA complexes (red) in the presence (light colors) and absence (dark colors) of 50 µM silvestrol. Comparison of clamping abilities of eIF4A1 and eIF4A2 with eIF4H (green) shows strong affinity of 4A paralogs for purine-only RNA. Competition was induced by addition of unlabelled $(AG)_{10}$ RNA. Data represent mean ± SD, n = 3. **D.** The binding affinity of eIF4A1 and eIF4A2 to 20 nM labelled $(AG)_{10}$ in the presence of $(CAA)_6CA$ competitor ssRNA at increasing molar excess in the presence of ATP was analysed using fluorescence polarisation. The apparent binding affinity of eIF4A1 and eIF4A2 to $(AG)_{10}$ has been determined after 1 h incubation together with the competitor RNA by fitting the binding data to the Hill-equation. Data has been plotted as the x-fold reduction of affinity by normalising the apparent affinities to the respective binding affinity in the absence of competitor RNA. Data represents mean ± SD, n = 3.

Fig. S8.

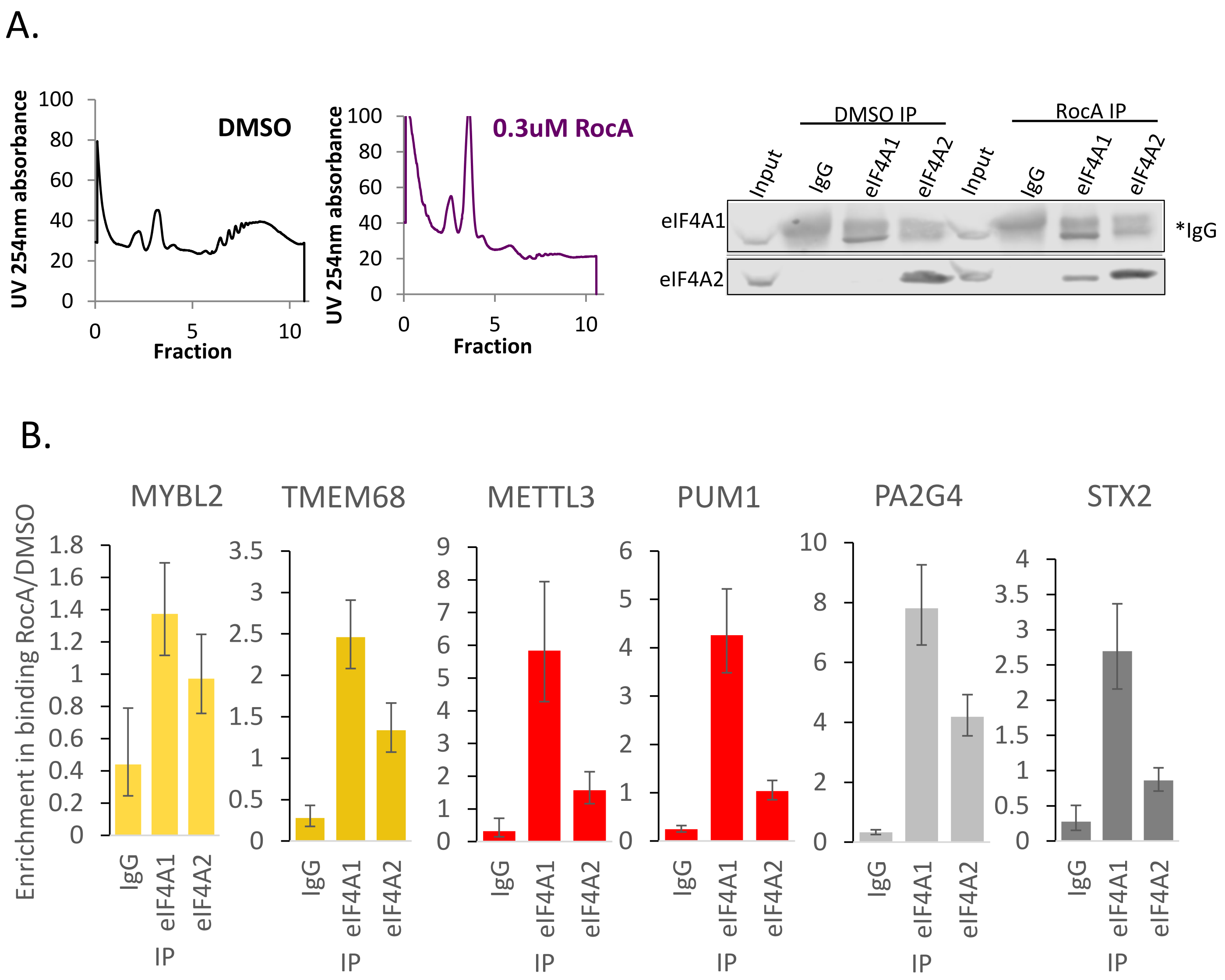

Fig. S8. RIP-qPCRs with RocA treatment show redistribution of eIF4A2-bound mRNAs to eIF4A1. A. Gradient profiles show the global change in translation following 30 minute 0.3uM RocA treatment. Western blot shows IPs for eIF4A1, eIF4A2 and IgG control following DMSO/RocA treatment. **B.** RIP-qPCR shows change in enrichment (dCT to input per condition and then enrichment RocA to DMSO) for binding IgG, eIF4A1 or eIF4A2 following RocA treatment. eIF4A1 clearly binds eIF4A2 mRNA targets following RocA treatment. Error bars are 95% confidence intervals.

Fig. S9.

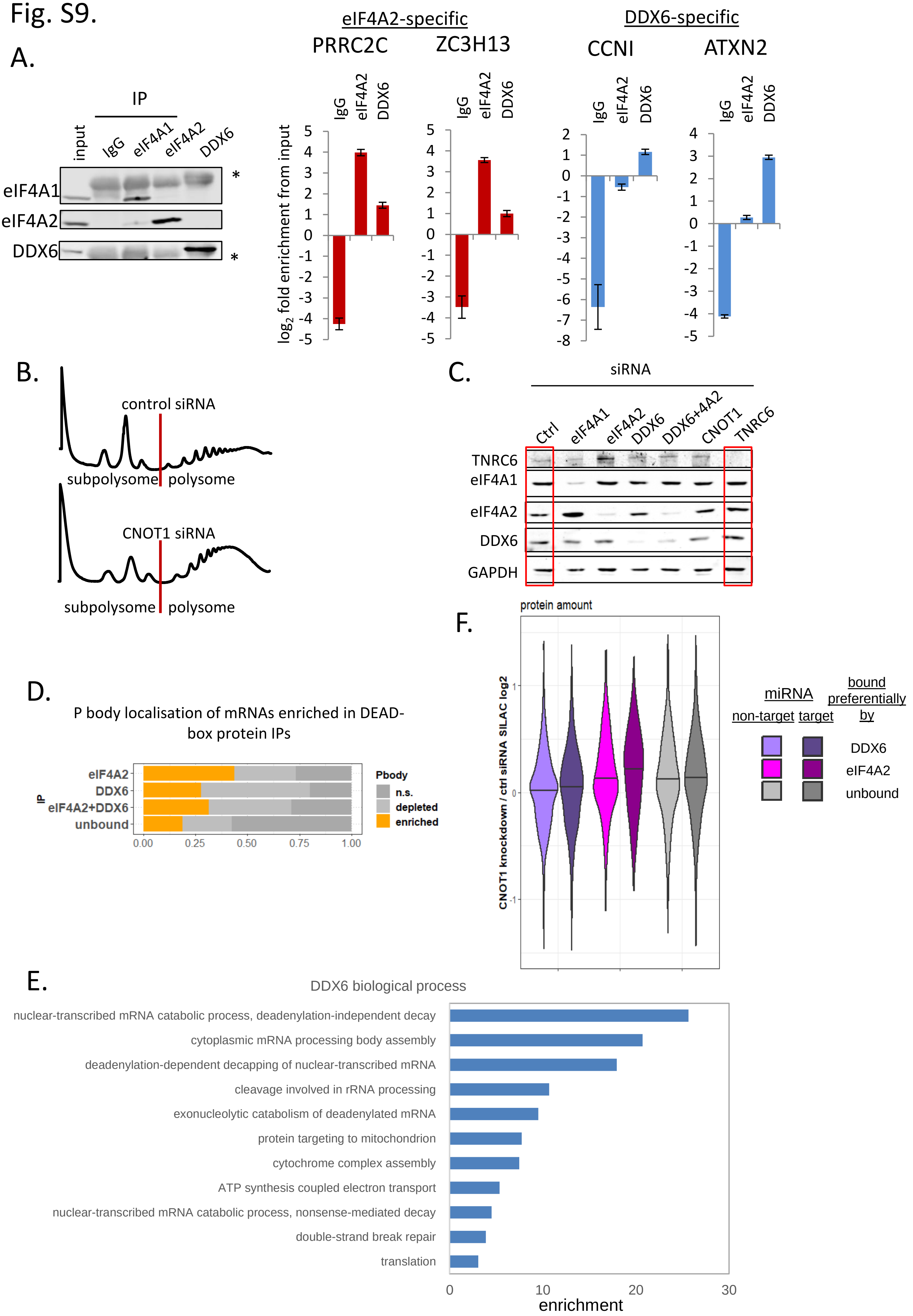
A.
eIF4A2-specific
DDX6-specific
PRRC2C
ZC3H13
CCNI
ATXN2
IP
input
IgG
eIF4A1
eIF4A2
DDX6
*
log2 fold enrichment from input
B.
control siRNA
subpolysome
polysome
CNOT1 siRNA
C.
siRNA
Ctrl
eIF4A1
eIF4A2
DDX6
DDX6+4A2
CNOT1
TNRC6
GAPDH
D.
P body localisation of mRNAs enriched in DEAD-box protein IPs
IP
eIF4A2
DDX6
eIF4A2+DDX6
unbound
Pbody
n.s.
depleted
enriched
0.00
0.25
0.50
0.75
1.00
F.
protein amount
CNOT1 knockdown / ctrl siRNA SILAC log2
miRNA
non-target
target
bound preferentially by
DDX6
eIF4A2
unbound
E.
DDX6 biological process
nuclear-transcribed mRNA catabolic process, deadenylation-independent decay
cytoplasmic mRNA processing body assembly
deadenylation-dependent decapping of nuclear-transcribed mRNA
cleavage involved in rRNA processing
exonucleolytic catabolism of deadenylated mRNA
protein targeting to mitochondrion
cytochrome complex assembly
ATP synthesis coupled electron transport
nuclear-transcribed mRNA catabolic process, nonsense-mediated decay
double-strand break repair
translation
0
10
20
30
enrichment

Fig. S9. Differential features of mRNAs bound to DEAD-box proteins. A. RIP-Seq reveals differential binding of DDX6 and eIF4A2 to mRNAs. A. Validation of enrichment of mRNAs in eIF4A2 and DDX6 IPs as in Fig. S4A. Western blot from Fig. 2A with the inclusion of DDX6 IP validation. **B. Polysome gradient analysis of CNOT1 knockdown.** Traces of sucrose density gradients used for preparation of sub- and polysomal fractions for CNOT1 knockdown translational efficiency analysis shown in Fig. 5B. HEK293 cells were transfected with either a CNOT1-specific siRNA or a control siRNA. 48h after transfection, cell lysates were subjected to sucrose density gradient fractionation. Samples as in Fig. 5B. Fractions were divided into subpolysomal and polysomal along the red line shown in the traces. Each of these was analysed by RNA-Seq in parallel with RNA extracted from an aliquot of input lysate. **C.** Western blot showing representative knockdown of TNRC6A. Whole blot is shown, but relevant lanes are highlighted with red rectangles. **D. eIF4A2-bound mRNAs tend to be more enriched in P-bodies** than those bound by either DDX6 as well as all mRNAs identified as unbound by either of the two DEAD-box proteins conditions. P-body enriched and depleted mRNAs were taken from Hubstenberger et al.[3] **E. DDX6 binds mRNAs encoding proteins involved in mRNA turnover and translation control.** Enriched GO terms for DDX6-bound mRNAs. **F. All miRNA target mRNAs bound more by eIF4A2 than DDX6 produce more protein upon CNOT1 depletion than non-target mRNAs or mRNAs bound more by DDX6.** Analysis performed as in Fig. 5D, except all mRNAs were taken into account, rather than uniquely bound ones. They are separated into groups based on preferential binding to either DDX6 or eIF4A2, or into mRNAs that are unbound.

Fig. S10.

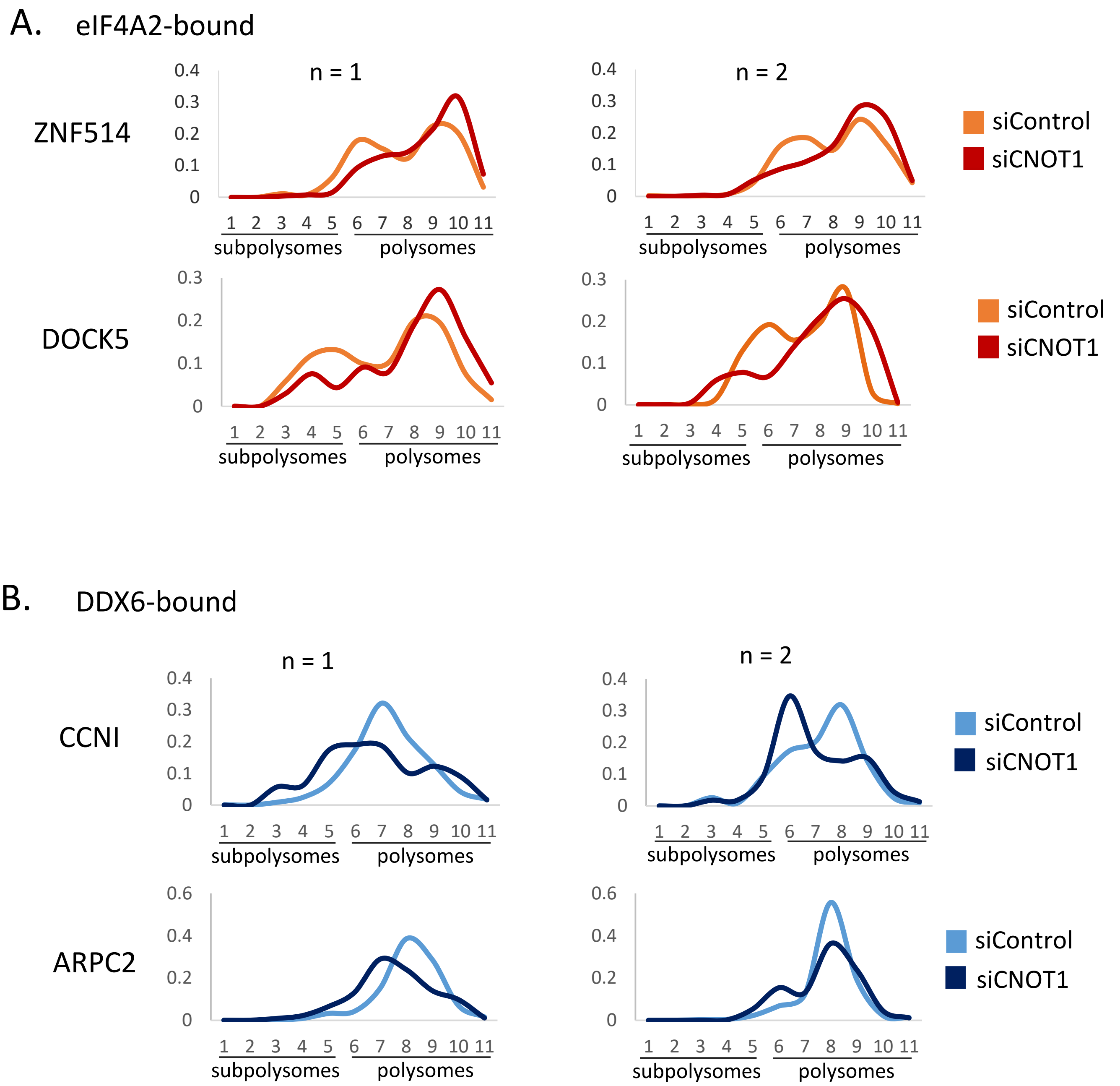

Fig. S10. Validation of eIF4A2- and DDX6-bound mRNA changes in polysome association. qPCR of gradient fractions (n=2) from control or CNOT1 siRNA treated samples. **A.** eIF4A2-bound mRNAs that contain miRNA target sites shift into polysomes following CNOT1 depletion. **B.** DDX6-bound mRNAs with miRNA target sites shift towards the sub-polysomes after CNOT1 depletion.

Fig. S11.

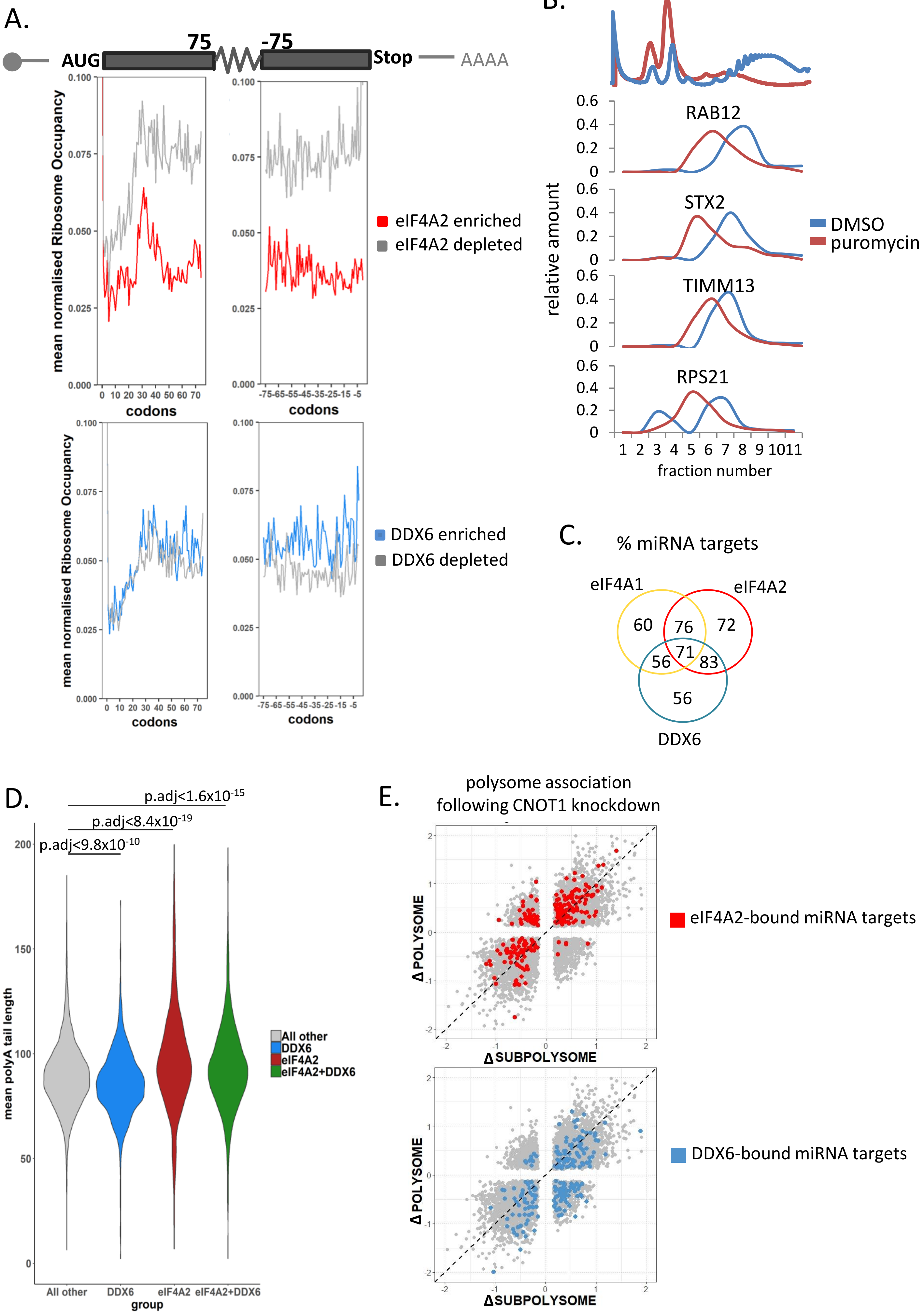
A.
AUG
75
-75
Stop
AAAA
mean normalised Ribosome Occupancy
codons
eIF4A2 enriched
eIF4A2 depleted
DDX6 enriched
DDX6 depleted
B.
RAB12
STX2
TIMM13
RPS21
relative amount
fraction number
DMSO
puromycin
C.
% miRNA targets
eIF4A1
eIF4A2
DDX6
60
76
72
71
56
83
56
D.
p.adj<1.6x10-15
p.adj<8.4x10-19
p.adj<9.8x10-10
mean polyA tail length
All other
DDX6
eIF4A2
eIF4A2+DDX6
group
E.
polysome association
following CNOT1 knockdown
Δ POLYSOME
Δ SUBPOLYSOME
eIF4A2-bound miRNA targets
DDX6-bound miRNA targets

Fig. S11. A. Differential ribosome occupancy of eIF4A2- and DDX6-bound messages compared to mRNAs specifically depleted in the eIF4A2 or DDX6 IP. Ribosome profiling as in Fig. 2C. Ribosome occupancy is the mean number of normalised ribosome footprints (to mRNA abundance (TPM)) 75 codons downstream of the AUG and upstream of the STOP codon. **B. DDX6-bound mRNAs are associated with polysomes in a puromycin-sensitive manner.** Lysates from HEK293 cells treated with either DMSO as control or puromycin for 3 minutes prior to harvest. Top graph shows the trace of RNA in the sucrose density gradient. The 4 bottom graphs show mRNAs being released from polysomes following puromycin treatment as assessed by RT-qPCR of individual gradient fractions. RAB12 and STX2 represent mRNAs bound by both eIF4A2 and DDX6, while TIMM13 and RPS21 are bound only by DDX6. **C. A greater proportion of eIF4A2-bound mRNAs are predicted miRNA targets.** mRNAs significantly changing in the subpolysomes and polysomes following CNOT1 depletion are separated into groups based on the proteins they bind to in the RIP-seq. Venn diagram shows the percentage of the miRNA targets for each group. **D. DDX6-bound mRNAs have shorter poly(A) tails.** Mean poly(A) tail length data taken from Subtelny et al[4] shows DDX6-bound mRNAs to have short poly(A) tails compared to mRNAs not enriched for binding either eIF4A2 or DDX6**. E. miRNA targets bound to eIF4A2 show a strong shift into polysomes following CNOT1 knockdown.** Graphs as in Fig. 5B showing miRNA target mRNAs bound to eIF4A2 and DDX6 that contain miRNA target sites (groups from Fig. 5B, but only ones with predicted miRNA target sites).

Supplemental References

1. Lu, W.-T. T. W.-T., Wilczynska, A., Smith, E. & Bushell, M. The diverse roles of the eIF4A family: you are the company you keep. *Biochem. Soc. Trans.* **42**, 166–72 (2014).

2. Meijer, H. A. A. *et al.* Translational repression and eIF4A2 activity are critical for microRNA-mediated gene regulation. *Science (80-.).* **340**, 82–85 (2013).

3. Hubstenberger, A. *et al.* P-Body Purification Reveals the Condensation of Repressed mRNA Regulons. *Mol. Cell* **68**, 144-157.e5 (2017).

4. Subtelny, A. O., Eichhorn, S. W., Chen, G. R., Sive, H. & Bartel, D. P. Poly(A)-tail profiling reveals an embryonic switch in translational control. *Nature* **508**, 66–71 (2014).
