# Supplementary material for: eIF4A2 drives repression of translation at initiation by Ccr4-Not through purine-rich motifs in the 5′UTR
Source: Genome Biol. 2019 Dec 2;20:262. doi: 10.1186/s13059-019-1857-2 (PMC6886185; doi:10.1186/s13059-019-1857-2)
Supplement: Supplementary file 2 — Additional file 2. Supplementary Tables S1-S2. [file 13059_2019_1857_MOESM2_ESM.pdf]

Table S1. Adjusted p-values for enrichment of conserved miRNA family targets among mRNAs enriched in the RIP-seq experiments and following TNRC6 knockdown Table represents the numerical values and miRNA family names for represented graphically in Fig. 5C.

conserved miRNA family	DDX6 & eIF4A2	eIF4A2	DDX6	TNRC6
miR-196abc	0.63421649	0.08776844	0.30536103	7.57E-05
miR-216b/216b-5p	0.85822051	0.95246836	0.46791323	0.11798271
miR-126-3p	0.64568697	0.05129455	0.55568456	0.05466153
miR-122/122a/1352	0.69870867	0.88510233	0.59621293	0.00518091
miR-191	0.04675424	0.19375776	0.67378935	0.13638918
miR-10abc/10a-5p	0.3693709	0.71415024	0.71833629	6.27E-08
miR-499-5p	0.6229291	0.19328321	0.73126783	8.04E-05
miR-99ab/100	0.62456261	0.46115437	0.75532763	0.0114281
miR-24/24ab/24-3p	0.00066443	0.77339002	0.80765168	0.00033434
miR-218/218a	0.14270163	0.50647564	0.8105381	1.96E-08
miR-183	0.57357324	0.35323984	0.84792688	3.56E-06
miR-137/137ab	0.04119696	0.72050629	0.85339637	1.53E-15
miR-21/590-5p	0.46573487	0.62433979	0.85811048	9.93E-10
miR-216a	0.99736583	0.86905695	0.88553305	0.20151329
miR-155	0.55331766	0.07639527	0.89224327	9.13E-05
miR-33ab/33-5p	0.22431503	0.84043163	0.89915523	1.92E-06
miR-219-5p/508/508-3p/4782-3p	0.17416997	0.72598502	0.90072508	1.06E-13
miR-490-3p	0.2800956	0.27508964	0.90305274	0.12718414
miR-135ab/135a-5p	0.09996024	0.38344116	0.90826771	1.38E-11
miR-208ab/208ab-3p	0.39866168	0.12375871	0.92015518	9.23E-07
miR-142-3p	0.71518543	0.54395955	0.92245098	9.00E-12
miR-138/138ab	0.02935179	0.03328175	0.92515405	1.67E-06
miR-133abc	0.08150865	0.59289049	0.93243164	4.33E-08
miR-184	0.15987382	0.27909718	0.9391545	0.010051
miR-33a-3p/365/365-3p	0.29043444	0.01852504	0.95247785	2.06E-09
miR-143/1721/4770	0.66286951	0.36756283	0.95639211	0.00249107
miR-383	0.46356607	0.13675972	0.95892175	0.37146425
miR-194	0.52368093	0.26570075	0.95979151	0.00015442
miR-124/124ab/506	0.06290108	0.95696045	0.96106003	1.40E-20
miR-34ac/34bc-5p/449abc/449c-5p	0.00011101	0.95092998	0.96134372	7.60E-09
miR-22/22-3p	0.00060838	0.88496536	0.96194616	1.96E-05
miR-503	0.04419078	0.2403605	0.96258293	6.72E-23
miR-25/32/92abc/363/363-3p/367	0.05718001	0.20967743	0.96382102	7.70E-47
miR-18ab/4735-3p	0.41659161	0.09060445	0.96730041	2.59E-08
miR-139-5p	0.97986407	0.43214391	0.96870496	0.0003193
miR-29abcd	0.05561036	0.22760598	0.9733345	2.40E-09
miR-210	0.05889326	0.59921187	0.9766741	0.01680871
miR-190/190ab	0.45090144	0.42635317	0.9769718	0.00571111
miR-375	0.849498	0.13601692	0.97913966	0.00195001
miR-200bc/429/548a	0.45535092	0.323934	0.98107455	3.18E-13
miR-153	0.08526926	0.84056723	0.98208039	9.61E-13
miR-125a-5p/125b-5p/351/670/4319	4.38E-06	0.16805592	0.98343926	7.74E-09
miR-96/507/1271	0.10368578	0.63771687	0.98361521	2.05E-10
miR-17/17-5p/20ab/20b-5p/93/106ab/427/518a-3p/519d	0.00979961	0.00198891	0.98438251	1.25E-95
miR-7/7ab	0.13280828	0.47169931	0.98493146	0.00665162
miR-129-5p/129ab-5p	0.99980546	0.50154606	0.98566111	0.26443367
miR-146ac/146b-5p	0.16715377	0.04502042	0.98611596	0.1297471
miR-150/5127	0.00390768	0.14003036	0.98764428	0.91863968
miR-148ab-3p/152	0.0258044	0.11770075	0.98811381	1.78E-16
miR-217	0.94112662	0.64897803	0.98832627	0.00025091
miR-214/761/3619-5p	5.03E-07	0.11667412	0.9900362	6.98E-10
miR-15abc/16/16abc/195/322/424/497/1907	0.03213509	0.01610821	0.99146379	6.44E-33
miR-31	0.35126103	0.83149309	0.99150807	0.00110683
miR-101/101ab	0.56524818	0.15082477	0.99262958	1.70E-17
miR-187	0.1773717	0.05970539	0.99366124	0.05781147
miR-193/193b/193a-3p	0.82158147	0.51603414	0.99375474	1.84E-05
miR-144	0.63064315	0.04701553	0.99411879	1.68E-17
miR-93/93a/105/106a/291a-3p/294/295/302abcde/372/373/428/519a/520be/520acd-3p/1378/1420ac	0.09626413	0.0356846	0.99454894	7.21E-42
miR-27abc/27a-3p	0.00814728	0.63959176	0.99455984	5.00E-15
miR-140/140-5p/876-3p/1244	0.5255503	0.71130038	0.99490515	6.34E-06
miR-338/338-3p	0.02938005	0.43104066	0.99628449	0.15805476
miR-182	0.15954985	0.85935381	0.99666001	1.43E-11
miR-132/212/212-3p	0.41693242	0.4834144	0.99715097	6.77E-09
miR-455-5p	0.80607221	0.25975512	0.99779002	0.0011328
miR-221/222/222ab/1928	0.62710441	0.22939568	0.99784841	1.63E-08
miR-103a/107/107ab	0.23455387	0.05015705	0.99821661	3.94E-21
miR-26ab/1297/4465	0.50942906	0.53545241	0.99824427	5.62E-18
let-7/98/4458/4500	0.07489199	0.09590379	0.99870673	1.03E-13
miR-30abcdef/30abe-5p/384-5p	0.16395544	0.51794032	0.99887812	1.39E-27
miR-223	0.8442671	0.05423629	0.99897155	0.00016754
miR-9/9ab	0.13462097	0.71085895	0.9990132	4.24E-16
miR-205/205ab	0.50225503	0.26488827	0.99935164	3.78E-07
miR-130ac/301ab/301b/301b-3p/454/721/4295/3666	0.03425468	0.05509918	0.99940899	2.32E-43
miR-23abc/23b-3p	0.21778469	0.65279499	0.99942183	4.10E-10
miR-128/128ab	0.00106361	0.40788502	0.99952282	2.67E-17
miR-1ab/206/613	0.59903832	0.93070128	0.99955771	2.38E-12
miR-425/425-5p/489	0.89022395	0.00524466	0.99961606	0.0184331
miR-199ab-5p	0.32037299	0.09917452	0.99962374	2.36E-05
miR-141/200a	0.91449454	0.07695385	0.99972127	7.31E-08
miR-203	0.94652409	0.1733933	0.99975095	0.00013203
miR-181abcd/4262	0.13695843	0.73533667	0.99981649	5.29E-22
miR-145	0.41281691	0.49542161	0.9998379	2.49E-08
miR-204/204b/211	0.59192609	0.03270783	0.99984511	0.00025059
miR-19ab	0.00710764	0.35596366	0.99997365	3.71E-71
miR-192/215	0.76537551	0.33328964	1	0.00469772
miR-451	0.39238908	0.35271101	1	0.02607869

Table S2. Primers used in the study.

Construct/Gene	Primer name	Sequence
Flag-eIF4A1-PRRVAA	4A1_PRRVAA_F	CTAGCACCCCGTCGAGTAGCGGCTCAGCAGATACAGAAGGTGG
	4A1_PRRVAA_R	CCACCTTCTGTATCTGCTGAGCCGCTACTCGACGGGGTGCTAG
Flag-eIF4A2-PRRVAA	4A2_PRRVAA_F	GCACTAGTATTGGCCCCCAGGAGAGTAGCGGCTCAACAGATCCAAAAGG
	4A2_PRRVAA_R	CCTTTTGGATCTGTTGAGCCGCTACTCTCCTGGGGGCCAATACTAGTGC
4A1N-4A2C/4A2N-4A1C	4A1 E18 BamHI mutF	GACAATGGCCCCGATGGGATGGAtCCCGAAGGCGTC
	4A1 E18 BamHI mutR	CTCTCGATGACGCCTTCGGGaTCCATCCCATCG
	4A2 D18 BamHI mutF	GGCGGCCCAGAGGGAATGGAtCCCGATGGTGTCATC
	4A2 D18 BamHI mutR	GCTCTCGATGACACCATCGGGaTCCATTCCCTCTG
4A1Δ1-16	4A1_16SalImutF	GATCCAGAGACAATGGCGTCGACGGGATGGAGCCCGAAG
	4A1_16SalImutR	CTTCGGGCTCCATCCCGTCGACGCCATTGTCTCTGGATC
4A2Δ1-13	4A2_13BamHImutF	GGATTATAACAGAGAACATGGATCCCCAGAGGGAATGG
	4A2_13BamHImutR	CCATTCCCTCTGGGGATCCATGTTCTCTGTTATAATCC
eIF4A2 point mutations	4A2_E101A_F	GATTGAGTTCAAGGCGACCCAAGCAC
	4A2_E101A_R	GTGCTTGGGTCGCCTTGAACTCAATC
	4A2_N143A_A150M_F	GGTGGAACAAATGTTCGAGCTGAAATGCAAAAACTGCAGATGGAAGCACCACATATTG
	4A2_N143A_A150M_R	CAATATGTGGTGCTTCCATCTGCAGTTTTTGCATTTCAGCTCGAACATTTGTTCCACC
	4A2_S207N_I208T_F	CCAAAAACTAAACACAAATACTCAGGTTGTGTTGC
	4A2_S207N_I208T_R	GCAACACAACCTGAGTATTTGTGTTTAGTTTTTGG
METTL3	METTL3_F	GAAAGACTATCCCTGGCACTC
	METTL3_R	GTACCTTTGCTTGAACGTG
TMEM68	TMEM68_F	TGGTGCAAGGAAAACAGTG
	TMEM68_R	TTATAAGTGCTGGTCCATCTTC
MYBL2	MYBL2_F	ATTGTGGATGAGGATGTGAAG
	MYBL2_R	GAGGCTGGAAGAGTTTGAAG
PUM1	PUM1_F	CGGAAGATCGTCATGCATAAG
	PUM1_R	CCAGAATGTGCTTGCCATAG
ZC3H13	ZC3H13_F	ATGATCGACGCCATGAAAG
	ZC3H13_R	TGTCATCTCTGTGGTCTCTAG
PRRC2C	PRRC2C_F	ACCAACATCTAGTCCCTTCC
	PRRC2C_R	GCTTCTGGTAGACAATGCTG
ATXN2	ATXN2_F	TCACACTTCAGATTTCAACCC
	ATXN2_R	CCTGACTGGTAGCGAGAAG
PA2G4	PA2G4_F	ACCATTATCCAGAATCCCACAG
	PA2G4_R	TTCTCTGTCCTGCATCCTTG
STX2	STX2_F	CTTCAGTGGATCTTCGGATACG
	STX2_R	GTCTGTGCCTCATTGTACTCC
RAB12	RAB12_F	GACAGAGAAATCACCAGGC
	RAB12_R	TCGTCCACATTGAAGTTATCC
TIMM13	TIMM13_F	ACAACTCCGAGCAGAAGTG
	TIMM13_R	TGGAAATGAACAGGGTGGG
CCNI	CCNI_F	TGGAAAAGGCAATCACTAGGG
	CCNI_R	GTTTCTGGGTAAAGGTTGAATTG
ARPC2	ARPC2_F	CAGCACAGTGTTTAAGGATG
	ARPC2_R	GTGTCTTTCAGCTCCAGAG
ZNF514	ZNF514_F	TTCTGGGCCTTCTAGTATCC
	ZNF514_R	ACTTGGCATTGATTCCTTGG
DOCK5	DOCK5_F	ACACCTATGGCCTCTATGTG
	DOCK5_R	CTGCCCCAACGAATTAGATAG
RPS21	RPS21_F	TCCGCTAGCAATCGCATC
	RPS21_R	GACTCACCCATCCTACGAATG
