# Supplementary material for: eIF4A2 drives repression of translation at initiation by Ccr4-Not through purine-rich motifs in the 5′UTR
Source: Genome Biol. 2019 Dec 2;20:262. doi: 10.1186/s13059-019-1857-2 (PMC6886185; doi:10.1186/s13059-019-1857-2)
Supplement: Supplementary file 3 — Additional file 3. Review history. [file 13059_2019_1857_MOESM3_ESM.docx]

Review History

**First round of review**

**Reviewer 1**

**Are you able to assess all statistics in the manuscript, including the appropriateness of statistical tests used?**

No, I do not feel adequately qualified to assess the statistics.

**Comments to author:**

The manuscript by Wilcynska et al, and Bushell co-corresponding, addresses the roles of related RNA helicases in human cells. The study provides strong evidence that the eIF4A2 paralog is functionally distinct from eIF4A1, despite having extremely high sequence identity. Molecular biology approaches identify residues that differ between the paralogs that contribute to the differential translation of a luciferase reporter. Unlike eIF4A1, eIF4A2 appears not to be a component of eIF4F, the cap-binding complex that stimulates translation initiation. Instead evidence is presented that eIF4A2 can associate with the negative regulator of translation and mRNA stability, the Ccr/NOT complex. The manuscript shows via a range of 'omics approaches that 4A1 and 4A2 interact with quite different sets of mRNAs. 4A2-enriched mRNAs differ from the 4A1-bound mRNAs in polysome association (4A2 less). These different RNAs appear to have different motif enrichments and ribosome occupancies, suggesting that sequence variations in 5'UTRs contribute to variations in 4A1/2 binding that in turn alter ribosome engagement. These in vivo studies are complemented by in vitro binding studies where isolated 4A1 or 2 binding to different sequences is evaluated. as is the impact of the 4A inhibitor silvestrol.

Overall the manuscript makes a very good case that eIF4A2 can act as a translational repressor, while eIF4A1 is known as an activator of protein synthesis. These studies make a novel, significant and interesting contribution that will be of interest to the translational control field.

However, towards the end of the results (specifically Fig. 5 and related supplementary figures) the authors extend their findings to investigate the link between 4A2, DDX6 and CNOT1. Shown earlier in the manuscript is evidence that a portion of the cellular CNOT1 micrates in a size exclusion column at the same mol wt as some of the eIF4A2. They also reciprocally Ip each other. CNOT1 has many roles in RNA processing and decay including a role in miRNA-mediated repression of translation. The authors conclude that 4A2 and NOT1 are acting together as part of the miRNA repression mechanism to repress translation at the initiation step. While the mechanism may be attractive to the authors, the evidence presented to support this idea is, at best, indirect and the descriptions and interpretations offered for this part of their story appear selective or that conclusions are overstated. This for the latter part of the manuscript the conclusions are not adequately supported by the data shown.

As outlined below there are a range of issues with the presentation or interpretation of some aspects of this manuscript which will need to be addressed. Some of these are simply performing/presenting additional computational analyses of the data, others are changing presentation and/or sentence wording. One will likely require an additional experiment. There are also numerous inconsistencies in labelling and reference to figures which suggests the authors have not proofread this manuscript carefully prior to submission.

Major

1	Can the authors explain how it is that the luciferase reporter translation is more susceptible to repression than FLAG-4A construct expression is. How does the DN 4A1 not repress its own synthesis? Do the various 5'UTR sequence elements identified later in the MS correlate with 4A1 DN sensitivity seen here?

2	2. Fig 2A. The Rip-Seq experiments show that most 4A1/2 enriched mRNAs are actually in common. While it is of interest to focus on differences, the text should make it clear that 80%+ of the targets are shared. Showing how these shared enriched mRNAs are distributed across polysomes and ribosome footprints and in the proteomics would also be appropriate and only add one additional sample to each panel (Figs 2B-F).

3	Figure S4A shows eIF4A1 and 2 binding to different specific mRNAs as a validation of the RIpSeq. However nowhere in the manuscript are the calculated Rip-Seq enrichments shown for these or any other genes. It should be possible to show in Figure S4A the equivalent data±error for these genes from RIpseq experiments. In addition the EdgeR files where the enrichments were calculated for the RIpSeq, polysome seq and equivalent TE calculations for the ribosome profiling could easily be included as a multi-tab excel file so that the data becomes a resource for the wider research community and researchers can relatively easily view data for their favorite genes. As a reviewer I am asked: Are sufficient details provided to allow replication and comparison with related analyses that may have been performed? At present the answer is no.

4	Including length-matched control datasets is a great control for the polysome analysis figure S4B/C, as it is clear that 4A2-bound mRNAs encode longer proteins. What is the reason for dividing the protein abundance data by mol wt from the SILAC experiment shown in Figure 2D? The peptide signal intensity alone should be proportional to peptide abundance. Surely this 'correction' will impact differently on 4A1 and 4A2 targets helping to create the distributions observed. Examining abundance of size matched control sets of proteins comparable to the length-matched data in S4B would help convince a reader there is an effect on protein abundance here. This experiment is important to the authors argument that there is any translational repression occurring on eIF4A2 target mRNAs as the polysome and ribosome footprint data can be explained by more rapid ribosome movement during elongation, as they acknowledge.

5	Fig.2E. 5' leader 80S footprints. The observation that 4A2 starts are enriched in upstream only starts, suggests there may be N-terminal extended ORFs as well as uORFs present in the data. The text here (page8/9) focuses on uORFs but does not refer to extensions in frame as an alternative. Is the footprint phasing sufficient to observe if this a possible explanation, or are these ORFs known to have upstream starts from prior investigations?

6	If the last 50 nts of 4A2 target RNA 5' leaders are often translated, does this mean that the purine rich motif (Fig 3B) is within the coding region of a uORF or extended ORF rather than in a region where scanning ribosomes would be found? Or are the mRNAs with 80S ribosomes here (Fig 3D) distinct from those with the motif (Fig 3B)?

7	The in vitro analyses of 4A1/2 binding to oligos provides some interesting data, but is not integrated with the in vivo findings well. One prediction is that adding silvestrol to cells should change the Rip-Seq or Rip-PCR results enabling 4A1 to now recover mRNAs bearing purine-rich motifs that were previously found in the 4A2 only Ip's. For example which of the 6 mRNAs in Fig S4A contain purine rich motifs and which change recovery in Ips following silvestrol treatment?

8	In Figure 5 and that accompanying supplementary figures it is not made clear why comparisons with the 4A1 rip-seq are suddenly dropped. It would be fairly easy to include the 4A1 data in the Venn, showing as 3 overlapping circles, and to include a second Venn showing numbers of miRNA targets in each of the 7 zones of the Venn. Similarly all the analyses in Fig 5 should be also compared with the 4A1 Rip data as an important control.

9	The presentation of Figure 5C is misleading when compared with Table S1. The Table shows ~75 families of which only 19 have P<0.05 in the 4A2+DDX6 column and 9 meet the same threshold in in the 4A2-only bound column. The coloring threshold used in %C must allow non-significant changes to be highlighted. This panel must be changed so that only significant enrichments are highlighted. The text page 12 lines 287/8 states that DDX6-only enriched mRNAs has significant overlaps with miRNA families, but Table S1 P values do not support this conclusion, none are significant.

10	I am confused by the apparently conflicting information in Figures S6C and D and S4D in reference to P bodies. Is the GO analysis (S6D-although called S6E in the text) covering all DDX6 enriched genes or the set termed 'DDX6 only' in other figure panels? S6C suggests that 4A2 mRNAs are highly enriched in mRNAs going to P bodies. However P bodies were not highlighted as significant in the GO analyses shown in S4D. Can the authors explain this? Are different gene subsets used in each analysis? Is it necessary to refer to P bodies at all in the results section here? Perhaps this text should be restricted to the discussion section, as there is no follow-up experimentation relating to P bodies presented.

11	CNOT1 siRNA knockdown studies.

a. The polysome profiles (Fig S6E) show that the siRNA to CNOT1 has a significant impact on the overall profile, shifting more ribosomes into heavier polysomes. This global change likely fully accounts for the changes in mRNA-ribosome engagement reported in S.Fig 8A. It also makes the DDX6 results (Fig.S8B) more striking. DDX6 mRNAs have longer polyA tails (Fig S7C) which typically leads to more active translation, yet here DDX6 target RNAs are shown shifting out of polysomes. One would assume that a shift out of polysomes would indicate translational change, likely repression, on loss of CNOT1. However, the silac data (Fig 5E, blue bars) all center around 0, so as many proteins are up as are down regulated. This does not seem to fit with the polysome fractionation data. How has the Silac data been normalised between the samples here? All the medians for each violin plot appear at or above 0. Is there a data processing error? The lack of fit between the polysomes and the silac data is referred to in the discussion, but no explanation is offered.

b. Does normalization of the RNA-seq samples for the polysome v subpolysomes fully neutralize the global effect of CNOT1 kd and mean that the changes shown in Fig 5D are above this?

c. As presented it is not clear which of the mRNAs in Fig 5D are miRNA targets and which changes are potentially attributable to CNOT1's other functions in general mRNA decay etc? In contrast in Fig 5E separates out miRNA and non-miRNA effects in the violin plots.

d. As per point 8 above, what is the pattern of eIF4A1 only targets in the CNOT1 kd polysome and silac analyses (Fig 5 D and E)? Fig 5A shows eIF4A1 binds to many miRNA target mRNAs.

12	In several places the manuscript text makes statements about things not yet shown.

a. Page 7, line 171/2. "To further investigate how the eIF4A2-bound mRNAs are being repressed…" At this point in the manuscript there is no evidence presented that these mRNAs are being repressed. So the text should be phrased: To further investigate 'if' the eIF4A2-bound mRNAs are being repressed…

b. Page 13 lines 310-11. "Thus far the data presented has suggested that mRNAs bound by 4A2 are repressed at initiation through their interaction with Ccr4-Not". This statement is not correct. No data shows that the interaction with ccr4-Not is necessary for 4A2 repression. This would require mutations that disrupt this interaction specifically which have not been made.

13	In the discussion there are multiple instances where the text overstates what has been shown. This is specifically where the authors are linking their observations to miRNA mediated translation repression. Here the evidence is indirect and would require significant experimental bolstering to match the claims made in the discussion. I suggest the authors have a radical reworking of this section and produce a more balanced discussion of their findings.

a. page 15. lines 259-361. P body statement is not supported by eIF4A2 GO analysis, unlike DDX6 GO data. Also it is not clear that eIF4A2-mRNA or DDX6-mRNA interactions are important for mRNA-P-body interaction. No data to support this are given. Does knockdown of either factor affect p bodies?

b. lines 365-66. Have not demonstrated that 4A2 repression of translation is specifically via interaction with CNOT1. There is no direct evidence that the independent interactions observed are linked. The data analysis for 4A2 focusses on a small subset of its interacting mRNAs. 628 4A2 only, while 3967 mRNAs that bind to both 4A1 and 4A2 are apparently sidelined. This suggests that the observations here likely represent one of many roles for 4A2 and that much remains to be deciphered about how 4A1 and 4A2 may act in tandem. The same holds for 4A2 v DDX6. The text does not appear to acknowledge this.

14	Conclusions section page 16, line 384. No text here.

minor

1. page 4, line 92 simply adding the words 'luciferase reporter' before 'translation' would help a reader understand the assay in Fig S1B without having to search through various pages to find the figure legends/methods.

2. Fig 1D. This table appears to simply report the numbers of peptides identified from each protein, rather than any quantitative assessment of the abundance of peptides. Have any other the other candidates been validated other than CNOT1

3. Figure 1F/S3A. the 4A1 probing is not adding anything to this experiment, as there is so much cross-reactivity. Why is this such an issue here when it was not in Fig 1E?

4. page 6, line 143. wording endogenous 'complex' should really be 'factors' or 'complexes' plural here.

5. page 7 171 'highly loaded with ribosomes'. These longer ORFs have the capacity to bind more ribosomes, the phrasing here could be improved.

6. Font sizes on multipanel figures lack uniformity. Frequently graph axis labels and scale font sizes are too small to see. For example Fig 2B looks good, but 2C, D, E and F are all different. Similar issues plague all figures in the manuscript.

7. SFig 6 legend does not correspond to the figure shown. It has an extra description -panel C that is not found in the Figure.

8. Fig 5A. The % enrichments could be indicated either on the figure or in the text (page 11, lines 268-269. Simply stating 'much higher' is not quantitative.

9. Table S1 header refers to Figure 3A, but the data is shown in Fig 5C.

10. page 14. lines 325-326. Poor sentence please revise to make sense. line 333 Fig. S6F not S6G.

11. p14 lines 344-5 poorly worded sentence.

12. page 15 lines 351-355. It is not clear of this paragraph is referring to miRNA-mediated translational repression or anything else.

13. page 15 lines 356-8. poor run-on sentence.

**Reviewer 2**

**Are you able to assess all statistics in the manuscript, including the appropriateness of statistical tests used?**

No, I do not feel adequately qualified to assess the statistics.

**Comments to author:**

This manuscript reports on evidence for functional distinctions between human the paralogous genes eIF4A1 and eIF4A2, with eIF4A1 playing the well-described role of eIF4A in promoting translation initiation while eIF4A2 acts in repressive complexes associated with microRNAs. These paralogous proteins are quite similar, and this work first reports data arguing that eIF4A2 does not interact with eIF4G, although this interaction can be restored by mutations that change several residues to match eIF4A1 sequences. Instead, it is argued that eIF4A2 interacts with repressive components of the CCR4-NOT complex. Next, RNA co-immunoprecipitation with 4A1 and 4A2 is used to argue that eIF4A2 associates with poorly translated RNAs, in contrast to eIF4A1. These distinct RNA interactions are linked to poly-purine motifs in the 5' UTRs of eIF4A2-bound mRNAs. In vitro binding experiments with recombinant protein and synthetic RNA targets are used to argue that eIF4A2 binds more tightly to these motifs, and an analogy is drawn with the action of silvestrol and related rocaglate drugs. Finally, it is argued that eIF4A2-bound RNAs are enriched for microRNA-repressed targets, based on an intersection between these data and knock-down of GW182 family proteins, and this is contrasted with the DEAD-box protein DDX6.

This manuscript continues an ongoing and important analysis of potential similarities and differences in the two, paralogous eIF4A proteins in humans. There are central conceptual and technical flaws in the analysis of eIF4A/RNA interactions, however, that affect central claims in the manuscript.

eIF4A is a DEAD-box RNA helicase that shows cooperative binding to RNA and ATP, followed by RNA-stimulated ATP hydrolysis and subsequent RNA release. 4A-RNA interactions are thus critically sensitive to the ATP hydrolysis cycle of the protein. In this manuscript, RNA immunoprecipitations are performed with no particular manipulation of ATP, and so the eIF4A proteins likely perform ATP hydrolysis, release RNAs bound in vivo, and redistribute in a complicated and unpredictable manner. As a result, the RNA immunoprecipitation experiments cannot be interpreted to reflect the in vivo binding status of these proteins.

The analysis of binding in vitro is carried out in the presence of non-hydrolyzable AMP-PNP. Under these conditions, eIF4A dissociates very slowly from RNA. This raises critical concerns about the competition experiments reported in Figure 4 — these probably don't reflect equilibrium measurements, and do not support the interpretations drawn in the paper.

Silvestrol and related rocaglate drugs are notable because they induce ATP-independent "clamping" of eIF4A proteins onto RNA. The present work analyzes these interactions only in the presence of AMP-PNP, however. Because of the fairly rapid ATPase cycle of eIF4A in vivo, the high RNA affinities reported in the ATP-bound state are irrelevant for translational inhibition. Instead, high-affinity, ATP-independent interactions are the key to silvestrol-based repression.

**Reviewer 3**

**Are you able to assess all statistics in the manuscript, including the appropriateness of statistical tests used?**

No, I do not feel adequately qualified to assess the statistics.

**Comments to author:**

This manuscript presents an elegant solution to a major conundrum in the field - Are human eIF4A1 and eIF4A2 functionally redundant or different? This question is even more important in the light of the finding that translational control by miRNA is mediated by CCR4-Not complex through eIF4A2. eIF4A1 and eIF4A2 are 90% identical to each other and therefore thought to have identical function - in mRNA unwinding related to eIF4F function. However, previous knockdown studies suggested that, while eIF4A1 is essential for cell's translation, eIF4A2 is not.

The evidence presented in this manuscript in favor of different - rather repressive- role for eIF4A2 is based on classical molecular biology work as well as IP-mass and a lot of RNAseq works (RIP-seq and 2-fraction [low-resolution] polysome profiling) and yet circumstantial at best. However, corroborating with the recent finding that chemical inhibitors turn eIF4A1 into a repressor against mRNA with purine-rich motifs, the work makes a reasonable proposal that eIF4A2 is normally a repressor against mRNA with purine-rich motifs and that CCR4-NOT essentially recruits eIF4A2 to miRNA-bound mRNA for its translational repression.

The major purpose of this review is to clarify the technical limitation of the methods used and more appropriately manipulate date presentation.

Major concerns:

1. As the authors are well aware, IP can pull down many physiologically irrelevant proteins. To better discuss physiological association, I strongly recommend you to treat data semi-quantitatively, using emPAI instead of SAF. This is shown to linearly correlate with the molar abundance of the component (1,2). In the translation field, the Asano group has adopted this to discuss the inclusion of eIF2, eIF2B and eIF3 in the IP complex with 5MP (3). Given the wide variation in the emPAI values (or even peptide counts) gained by the same amount of proteins, the average of emPAI value for CCR4/Not components vs eIF4A2 would make a good (allowable) estimation of "substoichiometric" complex formation.

2. Related to IP Mass, how did you differentiate eIF4A1 vs eIF4A2? Did you exclude fragments derived from common parts of the proteins? Also see minor concerns point 1.

3. Since you propose that "eIF4A2-bound message have an increased prevalence of translation initiation from upstream translation initiation sites" it is relevant to investigate whether it is due to increase in non-AUG initiation. To do so, sift the GTI-seq data according to the kind of start codons - AUG, CUG or GUG (the latter two for the most pronounced non-AUG codons) and examine whether eIF4A2 bound mRNAs are enriched for non-AUG initiation or not. Perturbation in initiation factor functions are known to cause regulation in non-AUG initiation (4,5).

4. Ribosome profiling data manipulation may be improved by including reanalysis of existing data from the same cells. The more mapped reads, the more dependable the ribosome density data. GWIPS and TRIPS-vis are the example of useful resources (6,7).

Minor concerns:

Results

1. Mass spec: Fig. 1D and Table S3 do not match. Under eIF4A1 IP, eIF4A2 is listed as 0 in Fig. 1D, but as 19 in Table S3. I could not find CNOT7 in Table S3, either.

2. Fig. 2D "This suggests eIF4A2 has a role in reducing translation initiation at the main AUG start codon at least partly on account of the presence of active uORFs in the 5'UTRs of target mRNAs." Data is suggested to partition into uORFs initiated by different kind of start codons, such as AUG, CUG and GUG.

Introduction

1. Ref 2-4 represent only a small portion of works on eIF4A. Here I suggest another paper, Watababe et al 2010, from yeast work characterizing the role of eIF4A in translation (8). Since the work from yeast is mentioned later, this work and the review, Hinnebusch et al 2007 (9), would be relevant to cite.

Reference:

1. Ishihama, Y., Oda, Y., Tabata, T., Sato, T., Nagasu, T., Rappsilber, J., and Mann, M. (2005) Exponentially Modified Protein Abundance Index (emPAI) for Estimation of Absolute Protein Amount in Proteomics by the Number of Sequenced Peptides per Protein. Mol Cell Proteomics 4, 1265-1272

2. Kudlicki, A. (2012) The Optimal Exponent Base for emPAI Is 6.5. PLos One 7, e32339

3. Kozel, C., Thompson, B., Hustak, S., Moore, C., Nakashima, A., Singh, C. R., Reid, M., Cox, C., Papadopoulos, E., Luna, R. E., Anderson, A., Tagami, H., Hiraishi, H., Slone, E. A., Yoshino, K.-i., Asano, M., Gillaspie, S., Nietfeld, J., Perchellet, J.-p., Rothenburg, S., Masai, H., Wagner, G., Beeser, A., Kikkawa, U., Fleming, S. D., and Asano, K. (2016) Overexpression of eIF5 or its protein mimic 5MP perturbs eIF2 function and induces ATF4 translation through delayed re-initiation. Nucl Acids Res 44, 8704-8713

4. Fijałkowska, D., Verbruggen, S., Ndah, E., Jonckheere, V., Menschaert, G., and Van Damme, P. (2017) eIF1 modulates the recognition of suboptimal translation initiation sites and steers gene expression via uORFs. Nucl Acids Res 45, 7997-8013

5. Tang, L., Morris, J., Wan, J., Moore, C., Gillaspie, S., Aube, E., Fujita, Y., Nanda, J., Anderson, A., Cox, C., Hiraishi, H., Dong, L., Singh, C. R., Saito, H., Witcher, M., Topisirovic, I., Qian, S.-B., and Asano, K. (2017) Competition between translation initiation factor eIF5 and its mimic protein 5MP determines non-AUG initiation rate genome-wide. Nucl Acids Res 45, 11941-11953

6. Kiniry, S. J., O'Connor, P. B. F., Michel, A. M., and Baranov, P. V. (2019) Trips-Viz: a transcriptome browser for exploring Ribo-Seq data. Nucl Acids Res 47, D847-D852

7. Michel, A. M., Kiniry, S. J., O'Connor, P. B. F., Mullan, J. P., and Baranov, P. V. (2018) GWIPS-viz: 2018 update. Nucl Acids Res 46, D823-D830

8. Watanabe, R., Murai, M. J., Singh, C. R., Fox, S., Ii, M., and Asano, K. (2010) The eIF4G HEAT domain promotes translation re-initiation in yeast both dependent on and independent of eIF4A mRNA helicase. J Biol Chem 285, 21922-21933

9. Hinnebusch, A. G., Dever, T. E., and Asano, K. (2007) Mechanism of translation initiation in the yeast Saccharomyces cerevisiae. in Translational Control in Biology and Medicine (Mathews, M. B., Sonenberg, N., and Hershey, J. W. B. eds.), Cold Spring Harbor Lab Press, Cold Spring Harbor, NY. pp 225-268

**Authors Response**

**Point-by-point responses to the reviewers’ comments:**

*We would like to thank you and the reviewers for the helpful comments on our manuscript. We have addressed all the points raised and feel that, thanks to the changes in the text and additional experiments, our manuscript is now much improved. Below we discuss each point in detail – citing reviewers’ comments in italics.*

*Reviewer #1*

*We thank the reviewer for the positive feedback and we hope that the alterations we have made improved the manuscript. We address the reviewer’s specific points below, but would like to first of all highlight that we have made considerable alterations to both the Results section and the Discussion, as suggested by the reviewer. We feel it now better reflects the data. In particular, we were prompted by the reviewer’s comments to reorganise the section regarding miRNAs and CNOT1 depletion.*

*Major points:*

*1.	Can the authors explain how it is that the luciferase reporter translation is more susceptible to repression than FLAG-4A construct expression is. How does the DN 4A1 not repress its own synthesis? Do the various 5'UTR sequence elements identified later in the MS correlate with 4A1 DN sensitivity seen here?*

*We would like to apologise for the mistake in the figure: the bars on the chart in Fig S1B were in the wrong order – this has now been corrected. Indeed, we do see that the translation of the eIF4A1 mutant is decreased compared to WT eIF4A1. This is also visible to a certain extent in Fig 1C. The reporter protein used in these assays – Renilla luciferase – has a fairly short halflife, so will be very sensitive to a dominant negative effect on global translation. We thank the reviewer for their perceptiveness. With regards to the second part of the question, this dominant negative mutant binds eIF4G more readily and creates and inactive 4F complex. Thus we would expect the effect of 5’UTRs to be rather complex in this case.*

*2. Fig 2A. The Rip-Seq experiments show that most 4A1/2 enriched mRNAs are actually in common. While it is of interest to focus on differences, the text should make it clear that 80%+ of the targets are shared. Showing how these shared enriched mRNAs are distributed across polysomes and ribosome footprints and in the proteomics would also be appropriate and only add one additional sample to each panel (Figs 2B-F).*

*We have now amended figure 2 and the text to include this group. The rationale behind focusing on the exclusive groups was to draw out the distinctions in the proteins’ respective binding. We see in a number of our analyses (many shown in Fig 2) that the overlap group has properties similar to that of the 4A2 group, only less pronounced. In fact, when we divide the gene overlap into mRNAs bound more by eIF4A1 or more by eIF4A2, we see that in all instances they behave similarly to the respective exclusive groups.*

*3.	Figure S4A shows eIF4A1 and 2 binding to different specific mRNAs as a validation of the RIpSeq. However nowhere in the manuscript are the calculated Rip-Seq enrichments shown for these or any other genes. It should be possible to show in Figure S4A the equivalent data±error for these genes from RIpseq experiments. In addition the EdgeR files where the enrichments were calculated for the RIpSeq, polysome seq and equivalent TE calculations for the ribosome profiling could easily be included as a multi-tab excel file so that the data becomes a resource for the wider research community and researchers can relatively easily view data for their favorite genes. As a reviewer I am asked: Are sufficient details provided to allow replication and comparison with related analyses that may have been performed? At present the answer is no.*

*We have now added the enrichment values to S4A but have also updated our GEO submissions to include the edgeR tables for RIP-Seq, polysome seq and TE calculations for ribosome profiling.*

*4.	Including length-matched control datasets is a great control for the polysome analysis figure S4B/C, as it is clear that 4A2-bound mRNAs encode longer proteins. What is the reason for dividing the protein abundance data by mol wt from the SILAC experiment shown in Figure 2D? The peptide signal intensity alone should be proportional to peptide abundance. Surely this 'correction' will impact differently on 4A1 and 4A2 targets helping to create the distributions observed. Examining abundance of size matched control sets of proteins comparable to the length-matched data in S4B would help convince a reader there is an effect on protein abundance here. This experiment is important to the authors argument that there is any translational repression occurring on eIF4A2 target mRNAs as the polysome and ribosome footprint data can be explained by more rapid ribosome movement during elongation, as they acknowledge.*

*The original values were derived from the reported sum of peptides intensities generated by MaxQuant which needed to be corrected for protein size. However, we have now improved this by reporting iBAQ values for the protein encoded by bound mRNAs. iBAQ is a widely-used measure of absolute protein quantity (Schwanhäusser, Nature 2011). This does not alter the conclusions drawn previously. In response to point 2 above we have also added the group of mRNAs bound by both eIF4A1 and eIF4A2 to this figure. We have added graphs showing the protein output of the length-matched controls (as in Fig. S4B) and we see eIF4A2-bound mRNAs have a lower protein output than the length-matched controls (Fig. S4C).*

*5.	Fig.2E. 5' leader 80S footprints. The observation that 4A2 starts are enriched in upstream only starts, suggests there may be N-terminal extended ORFs as well as uORFs present in the data. The text here (page8/9) focuses on uORFs but does not refer to extensions in frame as an alternative. Is the footprint phasing sufficient to observe if this a possible explanation, or are these ORFs known to have upstream starts from prior investigations?*

*We have now looked at the frame of the active upstream translation initiation sites (determined from GTI-seq data) in relation to the main annotated start codon. This shows most of the upstream translation occurs from sites not in frame (Fig. S5DE), supporting the conclusion the majority of these are genuine uORFs not extensions of the main ORF. In addition, if we look at the proportion which have a corresponding stop codon within the 5’UTR, we see eIF4A2-bound mRNAs are more likely to have a complete ORF upstream of the annotated start codon of the transcript, as shown below.*

*6	If the last 50 nts of 4A2 target RNA 5' leaders are often translated, does this mean that the purine rich motif (Fig 3B) is within the coding region of a uORF or extended ORF rather than in a region where scanning ribosomes would be found? Or are the mRNAs with 80S ribosomes here (Fig 3D) distinct from those with the motif (Fig 3B)?*

These are interesting but difficult questions to address. Below we show the potential uORF positions and GA stretches of greater than 4nt for each of the eIF4A2 validated genes. For these two genes it suggests the GA motifs tend to be within the uORF sequence. However, globally the purine-rich motifs are both present within the uORFs as well as outside them. Given the GTI-Seq data only looks at initiation sites, rather than mapping complete uORFs, we can only estimate where the Stop codons are – see above. Therefore, we do not feel we have the resolution to draw firm conclusions based on these estimations of the precise uORF sequence, and this would be an interesting direction for a follow-on study. We have now added text to the discussion to elaborate on this. In the analysis by Iwasaki et al 2016 Nature, data using uORF reporter constructs would suggest that the positioning of the purine motif downstream of the uTIS is sufficient to induce its usage following 0.03uM RocA inhibition (Fig. 4E of Iwasaki et al).

*7.	The in vitro analyses of 4A1/2 binding to oligos provides some interesting data, but is not integrated with the in vivo findings well. One prediction is that adding silvestrol to cells should change the Rip-Seq or Rip-PCR results enabling 4A1 to now recover mRNAs bearing purine-rich motifs that were previously found in the 4A2 only Ip's. For example which of the 6 mRNAs in Fig S4A contain purine rich motifs and which change recovery in Ips following silvestrol treatment?*

We thank the reviewer for this suggestion, conducting these experiments and the addition of this new data strengthens our conclusions. We have now conducted RNA-IP experiments treating cells with 0.3uM RocA (a silvestrol derivative) or DMSO as a control (Fig S8). qPCR for the mRNAs shown in figure S4A in these conditions shows that eIF4A1 has increased enrichment for binding these mRNAs with RocA treatment. mRNAs bound to eIF4A1 in control conditions show a slight further increase in eIF4A1 binding following RocA treatment. However, the increased binding of eIF4A1 to messages that are only bound by eIF4A2 in control conditions is far greater (Fig. S8B). This is exactly what we would expect for these purine motif-containing mRNAs in light of data from Iwasaki et al and clearly demonstrates that RocA enables eIF4A1 to recover these eIF4A2 purine-rich targets. We also see that RocA treatment does not increase eIF4A2 binding to its original targets, suggesting eIF4A2 is already maximally bound to theses mRNAs.

*8	In Figure 5 and that accompanying supplementary figures it is not made clear why comparisons with the 4A1 rip-seq are suddenly dropped. It would be fairly easy to include the 4A1 data in the Venn, showing as 3 overlapping circles, and to include a second Venn showing numbers of miRNA targets in each of the 7 zones of the Venn. Similarly all the analyses in Fig 5 should be also compared with the 4A1 Rip data as an important control.*

We have now reorganised this section and have included results for polysome sequencing of CNOT knockdowns for eIF4A1 targets (Fig. 5B). We have also moved the figure comparing miRNA targets of eIF4A1 and eIF4A2 bound mRNAs to Fig. 2E and as suggested we have added a Venn diagram with the 7 zones showing the percentage of these mRNAs that are miRNA targets, as predicted by TargetScan (Fig S11C).

If we are to dissect the analyses in figure 5C&D to the seven groups and then split by miRNA targets the groups would become particularly small and difficult to conclude from. One of the reasons why further in the figure we only look at eIF4A2 and DDX6 is because these proteins have been implicated to have roles in miRNA-mediated repression and interact with the Ccr4-Not complex via CNOT1. Whereas, eIF4A1 is described to function as part of the eIF4F translation initiation complex through its interaction with eIF4G. We feel the reorganised data now follows a more logical sequence and should address the reviewer’s concerns.

*9.	The presentation of Figure 5C is misleading when compared with Table S1. The Table shows ~75 families of which only 19 have P<0.05 in the 4A2+DDX6 column and 9 meet the same threshold in in the 4A2-only bound column. The coloring threshold used in %C must allow non-significant changes to be highlighted. This panel must be changed so that only significant enrichments are highlighted. The text page 12 lines 287/8 states that DDX6-only enriched mRNAs has significant overlaps with miRNA families, but Table S1 P values do not support this conclusion, none are significant.*

We completely agree with the reviewer and have now adjusted the heatmap to correctly reflect pvalues (we’ve taken a relaxed stringency to the adjusted p-value, but highlight this in the figure). We also thank the reviewer for spotting the error in the text – we have now corrected this.

*10	I am confused by the apparently conflicting information in Figures S6C and D and S4D in reference to P bodies. Is the GO analysis (S6D-although called S6E in the text) covering all DDX6 enriched genes or the set termed 'DDX6 only' in other figure panels? S6C suggests that 4A2 mRNAs are highly enriched in mRNAs going to P bodies. However P bodies were not highlighted as significant in the GO analyses shown in S4D. Can the authors explain this? Are different gene subsets used in each analysis? Is it necessary to refer to P bodies at all in the results section here? Perhaps this text should be restricted to the discussion section, as there is no follow-up experimentation relating to P bodies presented.*

We would like to draw the Reviewer’s attention to the fact that the GO analysis of eIF4A2-bound mRNAs looks at the function of the mRNAs and is unconnected to whether or not these mRNAs are actually preferentially sequestered in P bodies. The information about P-body targeted mRNAs comes from the P-body RNA-Seq data from Hubstenberger, 2017 Mol Cell. Meanwhile, the GO analysis showed the DDX6-bound mRNAs actually encode for P-body proteins, which is indicative of coordinated regulation. We have altered the text to make this clearer. We feel that given the involvement of the two proteins with the Ccr4-Not complex, miRNA-mediated repression and translation repression in general, it is of interest that it is eIF4A2 that binds to mRNAs targeted to P-bodies more prominently. However, we have now restricted the comments on this to the discussion.

*11.	CNOT1 siRNA knockdown studies.*

*a.	The polysome profiles (Fig S6E) show that the siRNA to CNOT1 has a significant impact on the overall profile, shifting more ribosomes into heavier polysomes. This global change likely fully accounts for the changes in mRNA-ribosome engagement reported in S.Fig 8A. It also makes the DDX6 results (Fig.S8B) more striking. DDX6 mRNAs have longer polyA tails (Fig S7C) which typically leads to more active translation, yet here DDX6 target RNAs are shown shifting out of polysomes. One would assume that a shift out of polysomes would indicate translational change, likely repression, on loss of CNOT1. However, the silac data (Fig 5E, blue bars) all center around 0, so as many proteins are up as are down regulated. This does not seem to fit with the polysome fractionation data. How has the Silac data been normalised between the samples here? All the medians for each violin plot appear at or above 0. Is there a data processing error? The lack of fit between the polysomes and the silac data is referred to in the discussion, but no explanation is offered.*

We’d like to draw the reviewer’s attention to the fact that the DDX6-bound mRNAs have shorter, not longer polyA tails (now Fig. S11D). As the reviewer points out, we have commented on the discrepancy between the polysomes and silac data and have now expanded this comment in the discussion. It may well be the result of a buffering effect that had been previously described (Oertlin C et al 2019 NAR). As this manuscript is not focused around DDX6 itself, we did not pursue this further.

MaxQuant normalized ratios are used to account for technical variations that occur from the forward and reverse labelling in the pulsed SILAC procedure. Most importantly we are interested in how the bound groups of mRNAs shift in comparison to the majority of mRNAs. We see the eIF4A2-bound mRNAs, particularly the miRNA targets increase in protein levels following CNOT1 depletion in agreement with our other data. As noted by the reviewer knockdown of CNOT1 results in a global shift towards polysomes, so we would expect a global increase in protein production following CNOT1 depletion which we observe.

*b.	Does normalization of the RNA-seq samples for the polysome v subpolysomes fully neutralize the global effect of CNOT1 kd and mean that the changes shown in Fig 5D are above this?*

The sub vs poly analysis only considers mRNAs that are significantly changed in each fraction (i.e. changed both in sub and poly), in our view this neutralises the effect of total mRNA changes and only brings out the differences over and above this. Given the distribution of genes with logFC>0.5, we would argue that the changes are large. Moreover, we are primarily concerned with mRNAs that change in a significant manner in opposite directions in the two fractions, so all these differences will be independent of the total mRNA level changes (which are of course also a part of the mechanism of the Ccr4-Not complex and this was why we took the approach of restricting the analysis to translational effects).

*c.	As presented it is not clear which of the mRNAs in Fig 5D are miRNA targets and which changes are potentially attributable to CNOT1's other functions in general mRNA decay etc? In contrast in Fig 5E separates out miRNA and non-miRNA effects in the violin plots.*

We have now amended Fig. 5 in response to comment 8 above and have added figure S11E highlighting the miRNA targets to address this comment.

*d.	As per point 8 above, what is the pattern of eIF4A1 only targets in the CNOT1 kd polysome and silac analyses (Fig 5 D and E)? Fig 5A shows eIF4A1 binds to many miRNA target mRNAs.*

First of all, we have changed the polysome figure to include the polysome graphs for eIF4A1 (Fig. 5B). We also argue that since eIF4A1 isn’t interacting with the Ccr4-Not complex, our primary focus is on DDX6 and eIF4A2. The pattern for polysome shifts for eIF4A1-bound mRNAs (in the “larger” groups looking only at 4A1 vs 4A2 binding, Fig. 2A Venn diagram), shows a very different pattern to that of the eIF4A2-bound ones. The corresponding SILAC CNOT knockdown data does not show considerable differences in protein levels either:

*12	In several places the manuscript text makes statements about things not yet shown.*

We have tried to correct these to make the paper clearer for the reader.

*a. Page 7, line 171/2. “To further investigate how the eIF4A2-bound mRNAs are being repressed…” At this point in the manuscript there is no evidence presented that these mRNAs are being repressed. So the text should be phrased: To further investigate ‘if’ the eIF4A2-bound mRNAs are being repressed…*

This has been corrected

*b.	Page 13 lines 310-11. “Thus far the data presented has suggested that mRNAs bound by 4A2 are repressed at initiation through their interaction with Ccr4-Not”. This statement is not correct. No data shows that the interaction with ccr4-Not is necessary for 4A2 repression. This would require mutations that disrupt this interaction specifically which have not been made.*

This has now been altered to say it is possibly occurring via this interaction.

*13 . In the discussion there are multiple instances where the text overstates what has been shown. This is specifically where the authors are linking their observations to miRNA mediated translation repression. Here the evidence is indirect and would require significant experimental bolstering to match the claims made in the discussion. I suggest the authors have a radical reworking of this section and produce a more balanced discussion of their findings.*

We have made significant alterations in the discussion to address the Reviewer’s concerns and now feel that it is a more balanced text.

*a. page 15. Lines 259-361. P body statement is not supported by eIF4A2 GO analysis, unlike DDX6 GO data. Also it is not clear that eIF4A2-mRNA or DDX6-mRNA interactions are important for mRNA-P-body interaction. No data to support this are given. Does knockdown of either factor affect p bodies?*

We have addressed this question in point 10 above and comments have been moved to the discussion. Knockdown of DDX6 is known to deplete cells of P bodies (Chu and Rana, 2006 PLOS Biology; and many others). Interestingly, we have observed that depletion of eIF4A2 has the effect of increasing Ago2 localisation to PBs (possibly a defect of recycling of the protein), but we have not included this data as we did not follow up the initial experiments.

*b.	lines 365-66. Have not demonstrated that 4A2 repression of translation is specifically via interaction with CNOT1. There is no direct evidence that the independent interactions observed are linked. The data analysis for 4A2 focusses on a small subset of its interacting mRNAs. 628 4A2 only, while 3967 mRNAs that bind to both 4A1 and 4A2 are apparently sidelined. This suggests that the observations here likely represent one of many roles for 4A2 and that much remains to be deciphered about how 4A1 and 4A2 may act in tandem. The same holds for 4A2 v DDX6. The text does not appear to acknowledge this.*

We agree that we have understated the effects on the overlap groups, mainly for simplicity. We felt that the effects would be clearest when looking at groups exclusively by the proteins to avoid observing a mix of mechanisms at play which would be difficult to dissect. We have added text in the discussion regarding this.

To give the reviewer an additional degree of confidence in the activity of eIF4A2 in concert with the Ccr4-Not complex, and ensure the shift in polysome association of eIF4A2-bound mRNAs with siCNOT1 shown in Fig. S10 is acting through eIF4A2, we conducted gradients with depletion of both eIF4A2 and DDX6 and we still see these eIF4A2 target mRNAs shift toward polysomes.

We performed a double knockdown of eIF4A2 and DDX6 to ensure there was no redundancy between the two proteins in their function as part of the Ccr4-Not complex, as we know this can occur (Meijer HA, 2019 NAR). We also draw the reviewer’s attention to Fig. S9F where we specifically did look at mRNAs simply more bound by DDX6 or more bound by 4A2, thus naturally including the overlapping mRNAs and saw that it was specifically the miRNA target 4A2-bound mRNAs that were showing increased protein production following CNOT1 knockdown. The corresponding polysome sequencing plots are:

*14	Conclusions section page 16, line 384. No text here.*

This has now been corrected.

*Minor points*

*a. page 4, line 92 simply adding the words ‘luciferase reporter’ before ‘translation’ would help a reader understand the assay in Fig S1B without having to search through various pages to find the figure legends/methods.*

This has now been corrected.

*2.	Fig 1D. This table appears to simply report the numbers of peptides identified from each protein, rather than any quantitative assessment of the abundance of peptides. Have any other the other candidates been validated other than CNOT1*

Yes, please see Fig. 1E as well as other experiments from a follow-on study, Meijer et al NAR 2019, where the interactions were confirmed in a different experimental context where these complexes were further purified using gel filtration. We’ve now added a sentence referring to this paper into the manuscript.

*3.	Figure 1F/S3A. the 4A1 probing is not adding anything to this experiment, as there is so much cross-reactivity. Why is this such an issue here when it was not in Fig 1E?*

Because the experiment presented in 1F/S3A is from gel filtration fractions (S3B). This is explained in line 138. We hope the text is clear enough here.

*4.	page 6, line 143. Wording endogenous ‘complex’ should really be ‘factors’ or ‘complexes’ plural here.*

This has now been corrected.

*5.	page 7 171 ‘highly loaded with ribosomes’. These longer ORFs have the capacity to bind more ribosomes, the phrasing here could be improved.*

We have extended the sentence to make this clearer.

*6.	Font sizes on multipanel figures lack uniformity. Frequently graph axis labels and scale font sizes are too small to see. For example Fig 2B looks good, but 2C, D, E and F are all different. Similar issues plague all figures in the manuscript.*

We have done our best to rectify this. Should the manuscript be accepted for publication, we can make further changes where needed.

*7.	SFig 6 legend does not correspond to the figure shown. It has an extra description -panel C that is not found in the Figure.*

This has now been corrected.

*8.	Fig 5A. The % enrichments could be indicated either on the figure or in the text (page 11, lines 268-269. Simply stating 'much higher' is not quantitative.*

The figure has now been corrected.

*9.	Table S1 header refers to Figure 3A, but the data is shown in Fig 5C.*

This has now been corrected.

*10.	page 14. lines 325-326. Poor sentence please revise to make sense. line 333 Fig. S6F not S6G.*

This has now been corrected.

*11.	p14 lines 344-5 poorly worded sentence.*

This has now been corrected.

*12.	page 15 lines 351-355. It is not clear of this paragraph is referring to miRNA-mediated translational repression or anything else.*

This has now been corrected.

*13.	page 15 lines 356-8. poor run-on sentence.*

This has now been corrected.

Reviewer #2:

*1. eIF4A is a DEAD-box RNA helicase that shows cooperative binding to RNA and ATP, followed by RNA-stimulated ATP hydrolysis and subsequent RNA release. 4A-RNA interactions are thus critically sensitive to the ATP hydrolysis cycle of the protein. In this manuscript, RNA immunoprecipitations are performed with no particular manipulation of ATP, and so the eIF4A proteins likely perform ATP hydrolysis, release RNAs bound in vivo, and redistribute in a complicated and unpredictable manner. As a result, the RNA immunoprecipitation experiments cannot be interpreted to reflect the in vivo binding status of these proteins.*

We agree with the reviewer that any experiment taken by itself has its limitations. Here we show that eIF4A2 interacts with the Ccr4-Not complex and examine the mRNAs associated with it by RIP-Seq. We have followed in the footsteps of others (Costello et al., Genome Biology, 2015) in our experimental approach and limited the IP time as well as lysate dilution as much as possible to minimise changes in the binding from that of the endogenous situation. Of course, during the IP procedure mRNAs may exchange their association, and thus to test the hypothesis that eIF4A2 interacts with Ccr4-Not complex and to verify that the RIP-Seq identified mRNAs bound by eIF4A2 were affected by this complex, we depleted CNOT1 and observed that the eIF4A2 bound mRNAs become more polysomal. In addition, we see a similar effect following depletion of both eIF4A2 and DDX6 – see major point 13B in the response to Reviewer #1. RIP-Seq is a well-known and widely used method for analysing RNA association with protein factors, including helicases, like eIF4A and DDX6 (Costello et al., Genome Biology, 2015; Rissland et al., Genome Biology, 2017; Iwasaki et al., Nature, 2016). However, to further validate and confirm association in vivo we have UV cross-linked the RNA before lysis and conducted IPs of both eIF4A1 and eIF4A2 (with more stringent washes at 600 mM NaCl) and looked at mRNAs enriched or depleted in both in our RIP-seq data. These data confirm specific association of the mRNAs in the endogenous setting and show that this is not affected greatly by cross-linking. Additionally, new data asked for by reviewer 1 has now been included which shows that RocA redistributes eIF4A2 bound mRNAs to eIF4A1 confirming these are specific interactions (Fig. S8).

*2. The analysis of binding in vitro is carried out in the presence of non-hydrolyzable AMP-PNP. Under these conditions, eIF4A dissociates very slowly from RNA. This raises critical concerns about the competition experiments reported in Figure 4 — these probably don't reflect equilibrium measurements, and do not support the interpretations drawn in the paper.*

These experiments were set up to examine the selectivity of the eIF4As for different oligos in a competitive setting i.e. with increased concentration of (CAA)_6_CA RNA. Both oligos were added at the same time in the presence of AMP-PNP, +/- Silvestrol and the association of FAM oligo with the eIF4As determined. We have changed the wording of the text, and figure to clarify this. As the reviewer states, if we were to use ATP in these reactions, the ATPase activity would lead to accelerated release of the RNA and this would complicate the kinetics of this reaction. In our experimental setup, the apparent affinity is mainly determined by the association rate of the proteins to the RNA oligos reflecting the binding selectivity, as stated in the main text. However, the off-rates for these oligos are all similar in magnitude, therefore again suggesting the major difference is in the selection of these oligos. Additionally, to fully addresses this we have done the experiments in the presence of ATP and observe a similar relationship, in that eIF4A2 shows selective binding to AG-RNA while eIF4A1 is not selective in a competitive environment (Fig. S7A).

*Silvestrol and related rocaglate drugs are notable because they induce ATP-independent "clamping" of eIF4A proteins onto RNA. The present work analyzes these interactions only in the presence of AMP-PNP, however. Because of the fairly rapid ATPase cycle of eIF4A in vivo, the high RNA affinities reported in the ATP-bound state are irrelevant for translational inhibition. Instead, high-affinity, ATP-independent interactions are the key to silvestrol-based repression.*

The reviewer is correct that Silvestrol induces binding of eIF4A1 to RNA in the absence of ATP. However, for silvestrol this has been shown to occur on GA-containing oligos only but not on CAA oligos (Iwasaki et al., Nature, 2016), which we have confirmed. In addition, in RNA release experiments, we also find that silvestrol clamps eIF4A1 onto AG-RNA only but not CAA-RNA in the presence of ATP:

Thus our data confirms Iwasaki and colleagues’ findings and take it a step further showing the clamping on to GA specifically is likely the mechanism of translational inhibition by silvestrol. Thus, to be able to assess the association of a range of oligos, we set up these experiments in the presence of non-hydrolysable AMP-PNP to compensate for the sequence-specific effects of silvestrol in order to allow us to compare the different oligos. However, we now also tested eIF4A1’s RNA-binding capacity in the absence of nucleotide – see below. In the absence of nucleotide eIF4A1 shows higher affinity to AG-RNA vs CA-RNA, as opposed to the conditions with AMPPNP (K_D_ (-AMPPNP) AG-RNA = 3.3 µM). As eIF4A1 does not show selective binding in a competition environment both with ATP and AMPPNP, this directly suggests that nucleotide binding of eIF4A1 functions against selective RNA-binding and thus translational inhibition.

Reviewer #3:

*Major concerns:*

*1.	As the authors are well aware, IP can pull down many physiologically irrelevant proteins. To better discuss physiological association, I strongly recommend you to treat data semi-quantitatively, using emPAI instead of SAF. This is shown to linearly correlate with the molar abundance of the component (1,2). In the translation field, the Asano group has adopted this to discuss the inclusion of eIF2, eIF2B and eIF3 in the IP complex with 5MP (3). Given the wide variation in the emPAI values (or even peptide counts) gained by the same amount of proteins, the average of emPAI value for CCR4/Not components vs eIF4A2 would make a good (allowable) estimation of "substoichiometric" complex formation.*

We completely agree with the Reviewer and apologise for not having included this information previously. The table in Fig 1.D has now been changed to emPAI values for both experiments (1 includes 2 technical replicates). We would also like to point out we used high stringency in our analysis.

*2.	Related to IP Mass, how did you differentiate eIF4A1 vs eIF4A2? Did you exclude fragments derived from common parts of the proteins? Also see minor concerns point 1.*

Only peptides that were unique to either protein were counted. We have added text to the figure legend to make this clear. Also, please note that since the IPs were actually performed on the eIF4A proteins and we know the antibodies are specific to each paralog (as has been documented in multiple publications – for eIF4A2 a few references are Galicia-Vazquez G 2012 RNA, Meijer HA Science 2013, Ahmed CS 2018 NAR, and multiple others), there was no concern about cross-reactivity within these IPs.

*3.	Since you propose that "eIF4A2-bound message have an increased prevalence of translation initiation from upstream translation initiation sites" it is relevant to investigate whether it is due to increase in non-AUG initiation. To do so, sift the GTI-seq data according to the kind of start codons - AUG, CUG or GUG (the latter two for the most pronounced non-AUG codons) and examine whether eIF4A2 bound mRNAs are enriched for non-AUG initiation or not. Perturbation in initiation factor functions are known to cause regulation in non-AUG initiation (4,5).*

We thank the reviewer for this important question. We have now conducted this analysis and find that non-AUG initiation is occurring (Fig. S5BC). When we split the groups of mRNAs by the impact on downstream translation initiation (as characterised by the GTI-seq data) we see that mRNAs with completely reduced initiation from the main annotated start codon have a greater likelihood of having upstream translation initiation from an AUG codon. Whereas mRNAs translating from both the annotated start codon and from upstream sites tend to have upstream sites starting with non-AUG codons.

*4.	Ribosome profiling data manipulation may be improved by including reanalysis of existing data from the same cells. The more mapped reads, the more dependable the ribosome density data. GWIPS and TRIPS-vis are the example of useful resources (6,7).*

We agree with the reviewer that ribosome profiling data can sometimes be underpowered. Therefore, we conducted a review of existing datasets. Having examined all human ribosome profiling datasets available of TRIPS-viz (Kiniry SJ, 2019 NAR), all of which have been published, only 5 out of 40 have a minimum of three biological replicates for the Ribo-Seq and only 30 out of 40 also have paired total mRNA samples. Looking at the 5 datasets in TRIPS-viz that do have three biological replicates from Xu 2016, Zur 2016, Cenik 2015, Gameiro 2018 and Reid 2018, the read composition is **50-90% rRNA** and the number of mapped reads is **1-10 million** in the control samples from these datasets. In contrast, our ribosome profiling has three biological replicates with corresponding total mRNA sequencing that contain **< 5% rRNA**. We agree with the reviewer that the more mapped reads, the more dependable the data, and therefore we have now included information on the number of mapped reads in the materials and methods section to make it clear that we have excellent quality ribosome density data. In our ribosome profiling dataset the number of mapped reads after read deduplication using UMIs and selection for reads which are 28-30 nt in length for each of our replicates is **23, 33 and 41 million** showing we have high quality ribosome profiling data.

*Minor concerns:*

*1.	Mass spec: Fig. 1D and Table S3 do not match. Under eIF4A1 IP, eIF4A2 is listed as 0 in Fig. 1D, but as 19 in Table S3. I could not find CNOT7 in Table S3, either.*

We do apologise for this. As discussed above, this has now been amended in the sense that Fig 1D now reports emPAI values and Table S3 has been removed.

*2.	Fig. 2D "This suggests eIF4A2 has a role in reducing translation initiation at the main AUG start codon at least partly on account of the presence of active uORFs in the 5'UTRs of target mRNAs." Data is suggested to partition into uORFs initiated by different kind of start codons, such as AUG, CUG and GUG.*

We have done this analysis, as discussed in point 3 above.

*1.	Ref 2-4 represent only a small portion of works on eIF4A. Here I suggest another paper, Watababe et al 2010, from yeast work characterizing the role of eIF4A in translation (8). Since the work from yeast is mentioned later, this work and the review, Hinnebusch et al 2007 (9), would be relevant to cite.*

We realise that there are many very important publications on eIF4A that we could not cite due to space constraints, but we have followed the reviewer’s suggestion and added these two important publications to the references. We thank the reviewer for the helpful suggestion.

**Second round of review**

**Reviewer 1**

Wilczynska et al GBIO-D-1901133_R1.

In this revised manuscript the authors have done a largely excellent job at addressing my previous concerns about the presentation of the work, including taking up suggestions for supplementary analyses that have in my view strengthened the main findings of the paper which is that highly related RNA helicases eIF4A1 and 4A2 target distinct mRNAs and that by applying a 4A1 inhibitor the specificity of eIF4A1 can be altered to mimic that of 4A2. The data further supports a model where eIF4A2 can repress translation initiation and that one mechanism by which this occurs may be via the micro-RNA repression pathway.

I do have some outstanding points that the authors should consider:

Original point 3.

Thank you for adding some of the missing information to figure S4A. However, for 4 of the 6 mRNAs only data for 4A1 or 4A2 are shown when it is important to show both sets of data for all mRNAs.

Original point 9.

The revised Figure 5C is an improvement on the original version. However, using a P value of 0.2 seems unjustified. The authors can make the same point by using a more typical P value cut off of 0.05 which will be less likely to draw criticism from readers.

errors/omissions/phrasing

1. P12, The new paragraph ‘To further explore the in vitro results in cells’…. This phrasing could be improved to make it clearer.

2. In Fig. S10 it would likely be helpful to readers to indicate the approximate positions of 40S/60S/80S and polysomes for each of the plots shown.

3. iBAC is not defined

4. P29 bottom, Says Fig 2C, then later Figure 2B

**Reviewer 2**

The revisions have not addressed my concerns with the original submission. In particular, the claim that eIF4A2 acts like silvestrol-inhibited eIF4A1 is not supported by the data. The notable effect of these flavagline drugs is to stabilize eIF4A1 bound to RNA in the presence of ADP or the absence of any nucleotide. This effect is not tested in the current paper, and finding it would be inconsistent with published literature.

In Figure S7A&B, the eIF4A2 affinity difference between GA and CAA RNA under "control" conditions is quite modest (~5x). Sequence-independent binding enhancement by silvestrol runs contrary to publications from other groups. The exchange experiment in Figure S7C is likewise consistent with a modest quantitative change in the off-rate for eIF4A2 that would presumably translate into the affinity change.

**Authors Response**

**Point-by-point responses to the reviewers’ comments:**

Reviewer #1: Wilczynska et al GBIO-D-1901133_R1.

I do have some outstanding points that the authors should consider:

Original point 3.

Thank you for adding some of the missing information to figure S4A. However, for 4 of the 6 mRNAs only data for 4A1 or 4A2 are shown when it is important to show both sets of data for all mRNAs.

*This has now been corrected.*

Original point 9.

The revised Figure 5C is an improvement on the original version. However, using a P value of 0.2 seems unjustified. The authors can make the same point by using a more typical P value cut off of 0.05 which will be less likely to draw criticism from readers.

*We have now corrected this to clearly show families with FDR< 0.05, but have also shown 0.05<FDR<0.2 to show that this slightly less stringent statistics, the trend still holds (note these are adjusted p-values, not p-values as stated by the reviewer).*

errors/omissions/phrasing

1. P12, The new paragraph 'To further explore the in vitro results in cells'…. This phrasing could be improved to make it clearer.

*This has been corrected*

2. In Fig. S10 it would likely be helpful to readers to indicate the approximate positions of 40S/60S/80S and polysomes for each of the plots shown.

*We have indicated the fractions which are polysmal and subpolysomal on the graphs*

3. iBAC is not defined

*Now defined in figure legend, with reference to original publication*

4. P29 bottom, Says Fig 2C, then later Figure 2B

*Corrected.*

Reviewer #2: The revisions have not addressed my concerns with the original submission. In particular, the claim that eIF4A2 acts like silvestrol-inhibited eIF4A1 is not supported by the data. The notable effect of these flavagline drugs is to stabilize eIF4A1 bound to RNA in the presence of ADP or the absence of any nucleotide. This effect is not tested in the current paper, and finding it would be inconsistent with published literature.

*We agree with the reviewer that we have not conducted all of the experiments required to unequivocally prove that eIF4A2 functions like eIF4A1 inhibited by the flavagline drugs. In fact, our data included in this manuscript examining the off-rates (Fig. S7C) suggest a different mechanism: while the selectivity of eIF4A2 for AG-RNA with ATP and AMPPNP (Fig 4C, Fig S7D) is the same as silvestrol-inhibited eIF4A1, their off-rates for this oligo are distinct under these conditions. Therefore, we do not claim that eIF4A2 acts identically to eIF4A1 inhibited by these drugs. To further clarify this, we have modified the text in the discussion to elaborate this point and carefully checked all text to make sure we have not overstated this. To the discussion we have added “We observe that the selectivity of eIF4A2 for AG-RNA is similar to that of silvestrol inhibited eIF4A1 in the presence of either AMPPNP or ATP (Fig 4C, Additional file 1: Fig. S7D). While this similarly is striking, the underlying molecular mechanism may be different; in fact, our data show that the off-rate for AG-RNA is different between eIF4A2 and silvestrol inhibited eIF4A1 (Additional file 1: Fig.S7C).”*

*To the best of our knowledge, no data is currently published examining off rates for either eIF4A1 or eIF4A2 in the absence of nucleotides. In addition, no one has focused on characterising eIF4A2 in comparison with eIF4A1. Our data are completely consistent with the most thorough examination of eIF4A1 affinity and selectivity (Iwasaki et al., Nature, 2016).*

In Figure S7A&B, the eIF4A2 affinity difference between GA and CAA RNA under "control" conditions is quite modest (~5x). Sequence-independent binding enhancement by silvestrol runs contrary to publications from other groups. The exchange experiment in Figure S7C is likewise consistent with a modest quantitative change in the off-rate for eIF4A2 that would presumably translate into the affinity change.

*We agree with the reviewer that the difference in affinities is modest and that it is completely consistent in our results shown in Fig 4AB, S7ABC. While it is modest, the different binding selectivity of eIF4A2 compared to eIF4A1 is explained by these differences. We have added a sentence to convey this point – highlighted in context: “This observation is consistent with the difference in affinity and kinetic stability of eIF4A2 with the CA-RNA (Fig 4B and Additional File 1: Fig. S7C). Thus, uninhibited eIF4A2 shows selectivity for purine sequences that is at a similar levels to silvestrol-inhibited eIF4A1 (Fig. 4C).”*

*Regarding the reviewer’s statement “Sequence-independent binding enhancement by silvestrol runs contrary to publications from other groups”: this might be based on confusing nomenclature around the use of affinity versus stability. Stability of eIF4A1 is increased only onto AG-RNA following inhibition with silvestrol while the affinity of all RNAs tested increases regardless of the sequence. Our data are completely consistent with Iwasaki et al., Nature, 2016 who show increased affinity to a number of RNA sequences tested, including (CAA)6CA and (GUGCU)4-RNA (Extended data Figure 8B). We have added text to clarify this point, highlighted here in context: “Addition of silvestrol increases binding of both proteins to RNA, regardless of sequence (Fig. 4AB, S7A), as has been reported for eIF4A1 previously37.”*
